# Supplementary material for: Single-cell and spatial transcriptomics reveals the spatiotemporal trajectory of the small peptide TAP4 in delaying postharvest fruit senescence
Source: Plant Commun. 2026 Apr 7;7(9):101846. doi: 10.1016/j.xplc.2026.101846 (PMC13589556; doi:10.1016/j.xplc.2026.101846)
Supplement: Supplementary file 1 [file mmc1.pdf]

Plant Communications, Volume

Supplemental information

Single-cell and spatial transcriptomics reveals the spatiotemporal trajectory of the small peptide TAP4 in delaying postharvest fruit senescence

Xin Li, Yajing Tian, Xu Duan, Mingyue Lv, Xingfeng Shao, Yingying Wei, Patrick Mason, Ruining Guan, Qiyue Wang, Xinyue Pang, Guoqiang Zhang, and Robert J. Henry

Title Page

Single-cell and Spatial Transcriptomics Reveals the Spatiotemporal Trajectory of the Small Peptide TAP4 in Delaying Postharvest Fruit Senescence

Xin Li[1,2,3], Yajing Tian[1], Xu Duan[1], Mingyue Lv[1], Xingfeng Shao[4], Yingying Wei[4], Patrick Mason[2], Ruining Guan[5], Qiyue Wang[5], Xinyue Pang[2,6,*], Guoqiang Zhang[7,8,*], Robert J Henry[2,9*]

[1] College of Food and Bioengineering, Henan University of Science and Technology, Luoyang 471023, China.

[2] Queensland Alliance for Agriculture & Food Innovation, Queensland Biosciences Precinct, The University of Queensland, St Lucia QLD 4072, Australia.

[3] The National and Local Joint Engineering Laboratory of High Efficiency and Superior-Quality Cultivation and Fruit Deep Processing Technology of Characteristic Fruit Trees in South Xinjiang, Tarim University, Alar, 843300, China.

[4] Zhejiang-Malaysia Joint Research Laboratory for Agricultural Product Processing and Nutrition, College of Food Science and Engineering, Ningbo University, Ningbo 315800, China.

[5] School of Pharmacy, Lanzhou University, Lanzhou 730000, China.

[6] College of Medical Technology and Engineering, Henan University of Science and Technology, Luoyang 471023, China.

[7] Wuhu Green Food Industrial Research Institute Co., Ltd, Wuhu 241000, China.

[8] College of Biological and Food Engineering, Anhui Polytechnic University, Wuhu 241000, China.

[9] VinUni Big Data Research Institute, VinUniversity, Hanoi, Vietnam

*Corresponding author. Email: pangxy78@haust.edu.cn (X.Y. Pang); guoqiang2008@163.com (G.Q. Zhang); robert.henry@uq.edu.au (R.J. Henry).

Supporting information

The following materials are available in the online version of this article.

Figure S1. Analysis of the activity of the trypsin autolysis peptides.

Figure S2. Quality control of single-cell data (A) and co-expression patterns of marker genes (B) in the three samples.

Figure 3. RCTD and CARD methods for correlation analysis between stRNA-seq and scRNA-seq.

Figure S4. Expression of marker genes for the five components across three samples and five components.

Figure S5. Pseudotime analysis of cells of CK, Post, and TAP4 samples.

Figure S6. Metabolomics analysis of the pericarp of *H. undatus.*

Figure S7. Effects of silencing key genes on fruit phenotype and related gene expression in *H. undatus*.

Figure S8. Effects of TAP4 treatment on the postharvest microbial diversity of *H. undatus* fruit.

Supplemental Data 1. Activities of trypsin and autolytic peptide TAP4.

Sheet1 Superoxide scavenging activities of trypsin.

Sheet2 Effect of TAP4 on stoma in *H. undatus* fruit during postharvest storage.

Sheet3 Effect of TAP4 on cell size of *H. undatus* fruit during postharvest storage.

Sheet4 Effect of TAP4 on fluorescence intensity detected by RNA-FISH in *H. undatus* fruit during postharvest storage.

Sheet5 Effect of TAP4 on ROS detected by H_2DCFDA in *H. undatus* fruit during postharvest storage.

Sheet6 Effect of TAP4 on DAB staining in *H. undatus* fruit during postharvest storage.

Sheet7 Effect of TAP4 on lesion area of *H. undatus* fruit during postharvest storage.

Sheet8 Effect of TAP4 on callose deposition in *H. undatus* fruit during postharvest storage.

Supplemental Data 2. Information of trypsin autolytic peptides.

Sheet1 Identification of trypsin autolytic peptides by mass spectrometry.

Sheet2 The proportion of various amino acids in the seven key polypeptides.

Supplemental Data 3. Summary of the transcriptomic cell data in *H. undatus* pericarp samples.

Sheet1 Quality control information for the single-cell transcriptomic data.

Sheet2 Quality control information for the spatial transcriptomic data.

Sheet3 Expression of all 27735 genes in CK, Post, and TAP4 samples.

Sheet4 ROGUE value of 9 subcluster cells of EX, ME, and ENF cells.

Supplemental Data 4. Cluster information for 26 cell clusters of *H. undatus* pericarp cells.

Sheet 1 A comparative analysis statistical table of cell clusters presented by sample.

Sheet 2 A comparative analysis statistical table of cell types presented by sample.

Supplemental Data 5. Cluster-enriched gene list and gene descriptions for 26 cell clusters.

Supplemental Data 6. Correlation between 26 cell clusters.

Supplemental Data 7. Expression of pseudotime related immune genes.

Sheet 1 Regulatory patterns of 147 genes across 26 cell clusters.

Sheet 2 Expression levels of 147 genes in the three components of the three samples.

Supplemental Data 8. GRN parameters of pseudotime related genes by Cytoscape.

Sheet1 Parameters of nodes in the GRN.

Sheet2 Top20 nodes in PRGs network ranked by 8 effective methods of cytoHubba.

Sheet3 Information of all top20 nodes of GRN.

Sheet4 Expression of these top nodes in EX, ME, and ENF components in three samples.

Supplemental Data 9. Information of metabolites regulated by TAP4 in *H. undatus* analyzed by the SWATH method for mass spectrometry detection.

Sheet1 Information of top 26 metabolites regulated by TAP4.

Sheet2 Information of 3 betacyanins.

Sheet3 Information of all 3,015 metabolites detected in *H. undatus*.

Supplemental Data 10. Information of co-expression network among transcript factors, genes, and metabolites.

Supplemental Data 11. Information of hub genes shown in the heatmap of pseudotime analysis.

Sheet1 Information of genes in figure 4F.

Sheet2 Information of genes in figure 4G.

Supplemental Data 12. Key resources used in the current work.

Supplemental Data 13. Primer sequences used in this work.

Sheet1 Primers used in VIGS.

Sheet2 Primers used in RT-qPCR.

Sheet3 Primers used in RNA-FISH.

Supplementary Figures:

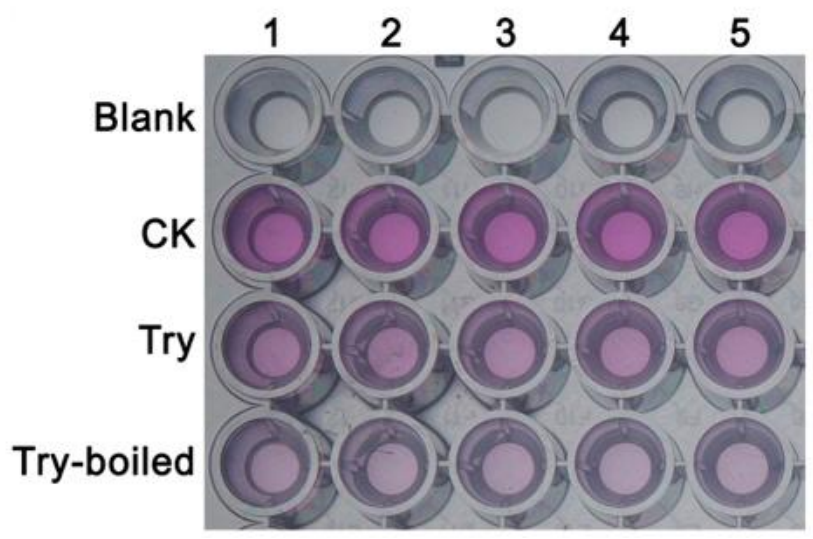

Figure S1: Analysis of the activity of the trypsin autolysis peptides. Color of the wells in the 96-well plate indicates the changes in superoxide anion levels after trypsin inactivation by boiling. The five columns represent five parallel samples for each group.

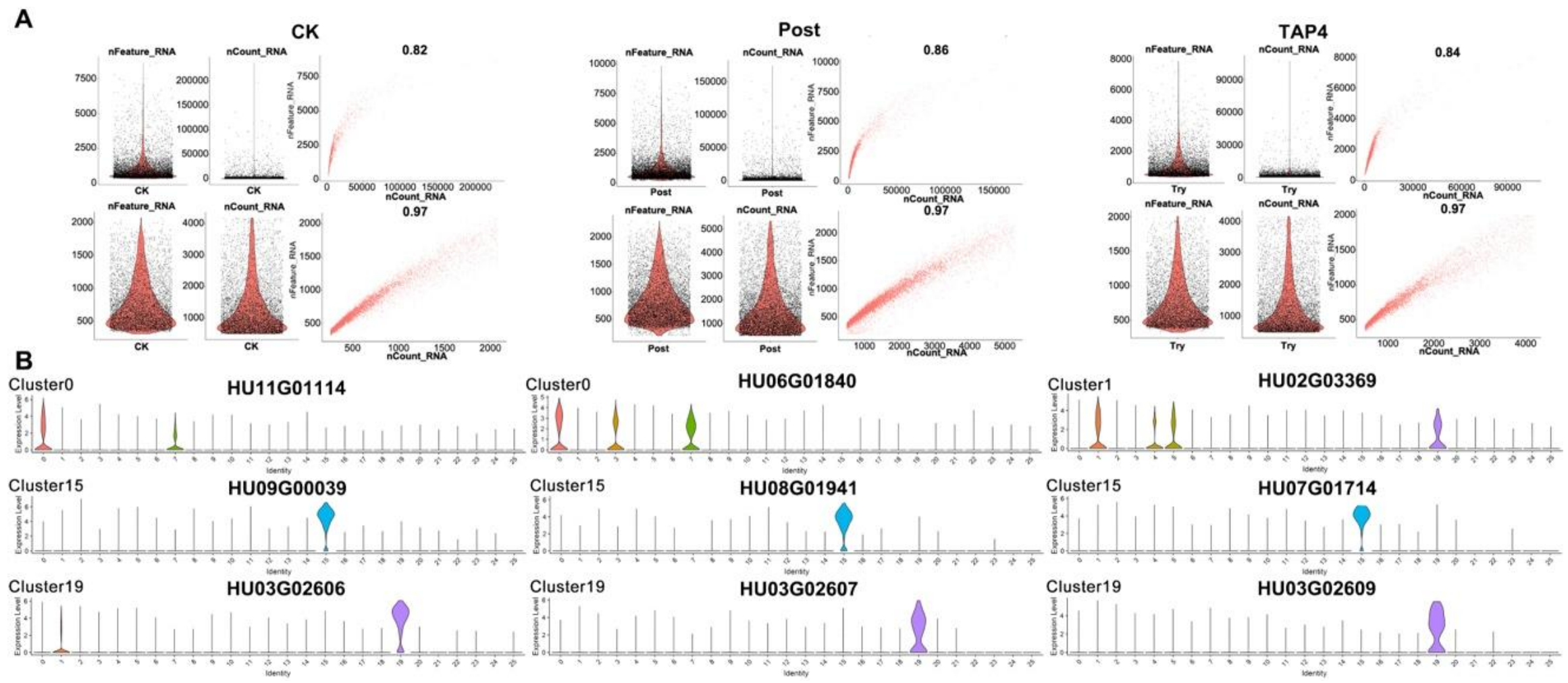

Figure S2: Quality control of single-cell data (A) and co-expression patterns of marker genes (B) in the three samples.

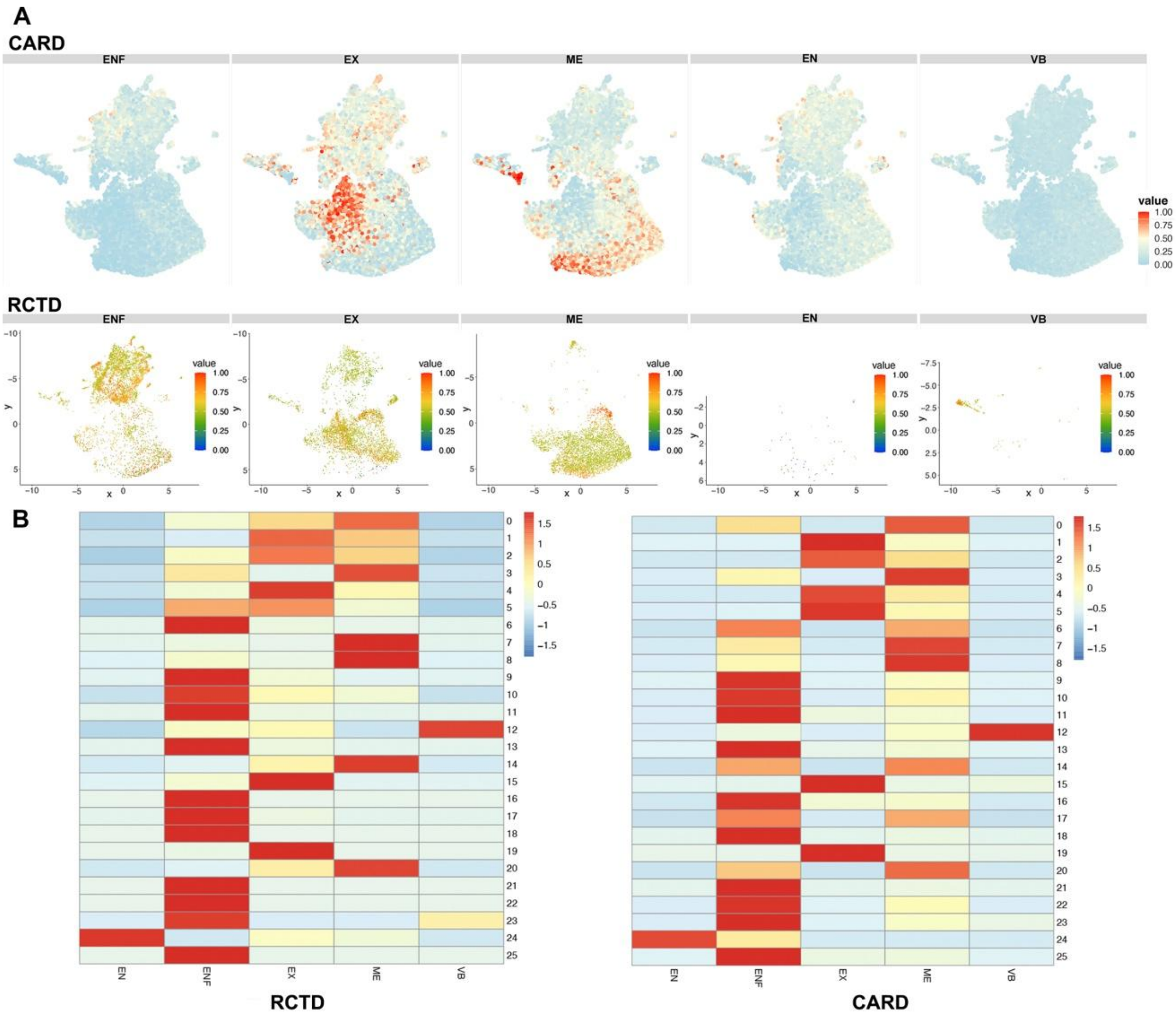

Figure S3: RCTD and CARD methods for correlation analysis between stRNA-seq and scRNA-seq. Heat dot plots in the UMAP (A) and the color bands in the heatmap (B) illustrate five components, respectively. The color gradient from red to blue indicates correlations ranging from high to low.

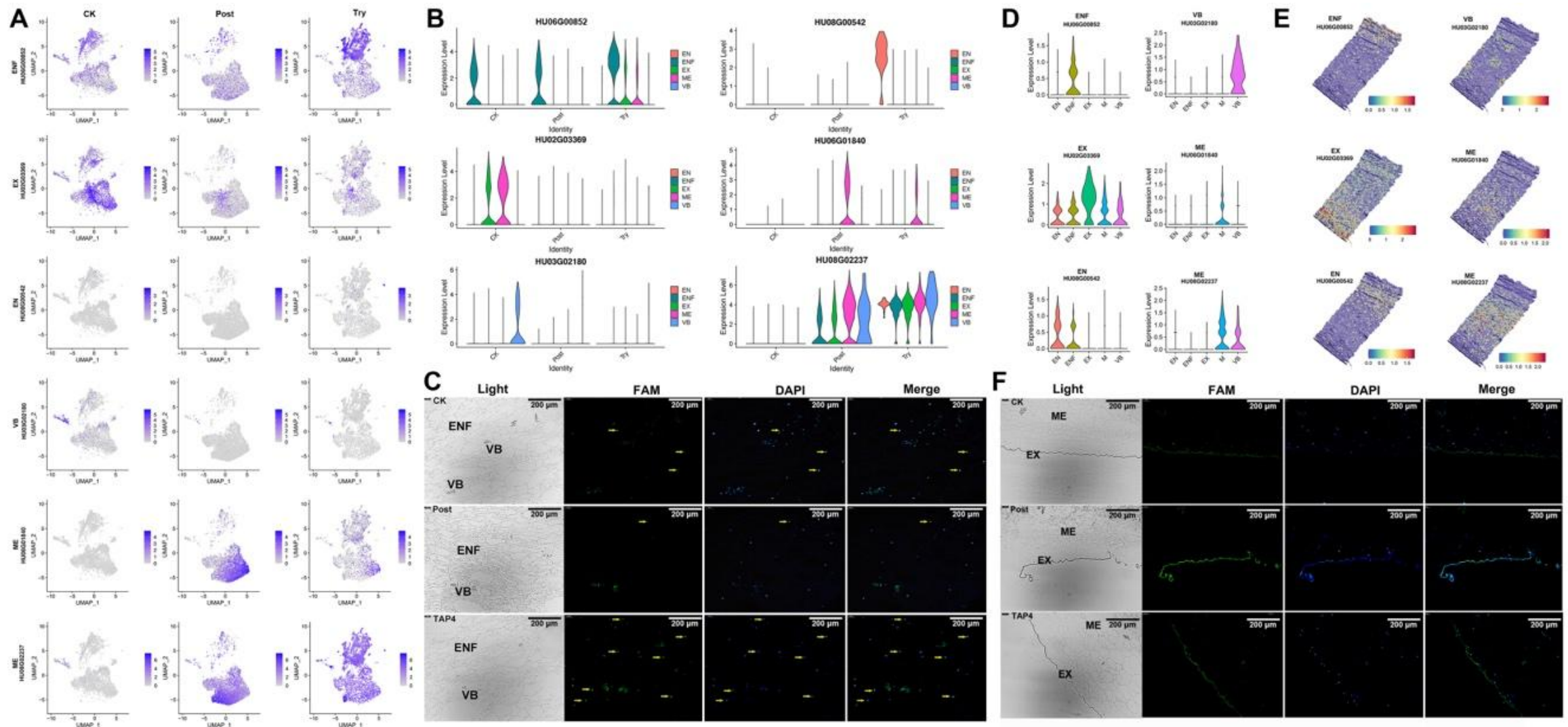

Figure S4: Expression of marker genes for the five components across three samples and five components

(A) The UMAP plots displayed the expression of each gene in the three samples.

(B) The violin plots illustrated the expression of each gene across the five components in the three samples. The three violin plots, from left to right, showed gene expression across the five components and three samples, respectively.

(C and F) RNA-FISH revealed the expression of the marker gene HU06G00852 and HU02G03369 in the endocarp and exocarp of the three samples, respectively.

(D-E) Spatial transcriptomic mapping (D) showing the spatial localization of marker gene expression, along with the corresponding violin plot (E).

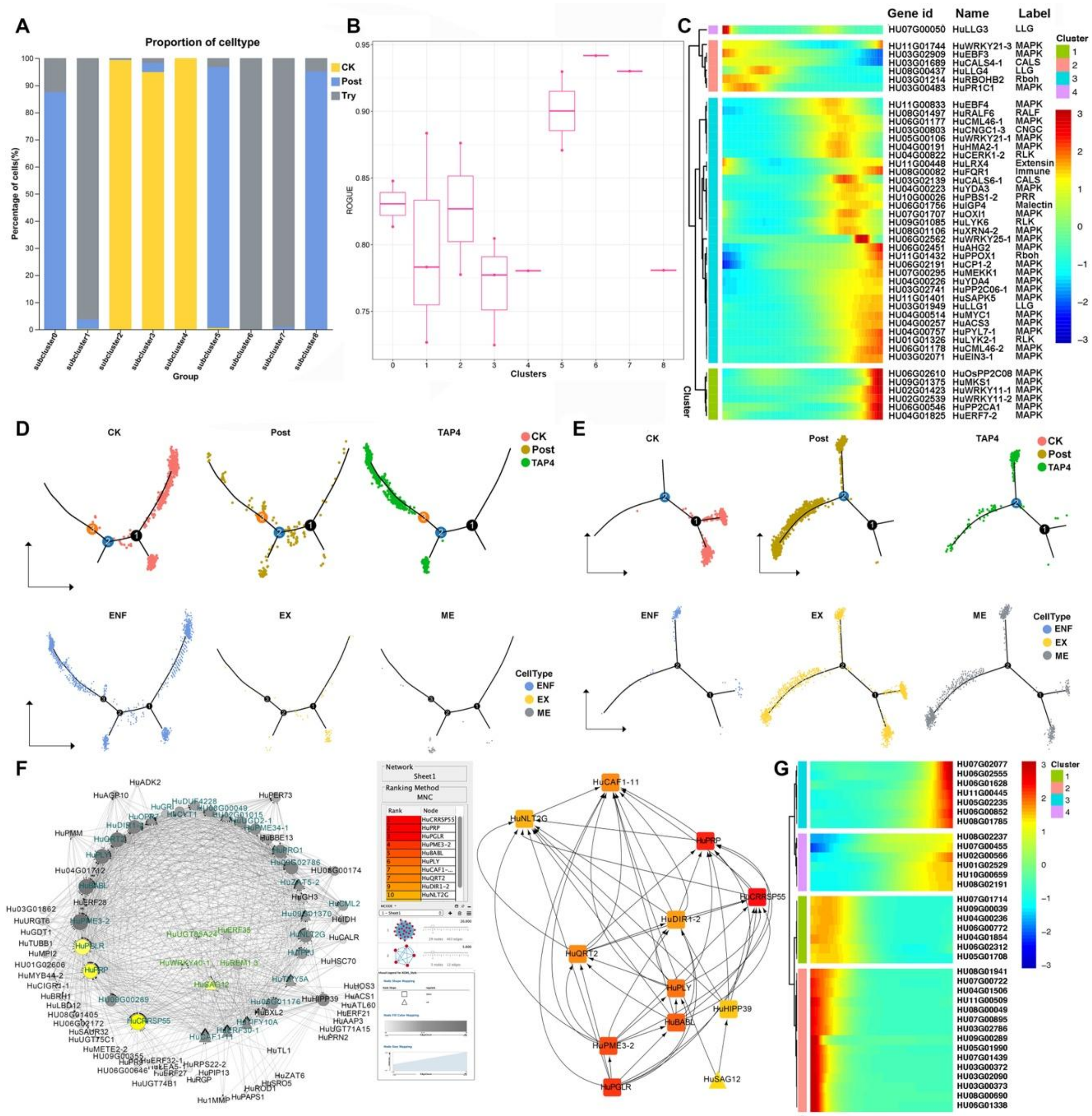

Figure S5: Pseudotime analysis of cells of CK, Post, and TAP4 samples

(A) Bar charts illustrating the statistical proportions of CK, Post, and TAP sample cells within each subcluster of EX, ME, and ENF cells.

(B) ROGUE evaluated the cluster purity of the nine subpopulations. Higher ROGUE values indicate better cell cluster quality. A ROGUE value of 1 represents a completely pure cell population, whereas values approaching 0 indicate highly heterogeneous mixed populations.

(C) Heatmap of expression of immune related genes along the pseudotime progression.

(D-E) Cells from the three samples (up) and three components (bottom) were mapped onto the pseudotime trajectory of cells high related to up- (D) and down-regulated (E) genes by TAP4.

(F) Overall diagram of the GRN (left) and the hierarchical layout of the top 12 nodes calculated by CytoHubba (right) of top 100 genes related to pseudotime from the MXF_1595 cells. The module parameters obtained from the MCODE plugin, the top nodes identified by the CytoHubba plugin, and the network drawing annotations are displayed in the center.

(G) Heatmap of expression of hub genes along the pseudotime progression.

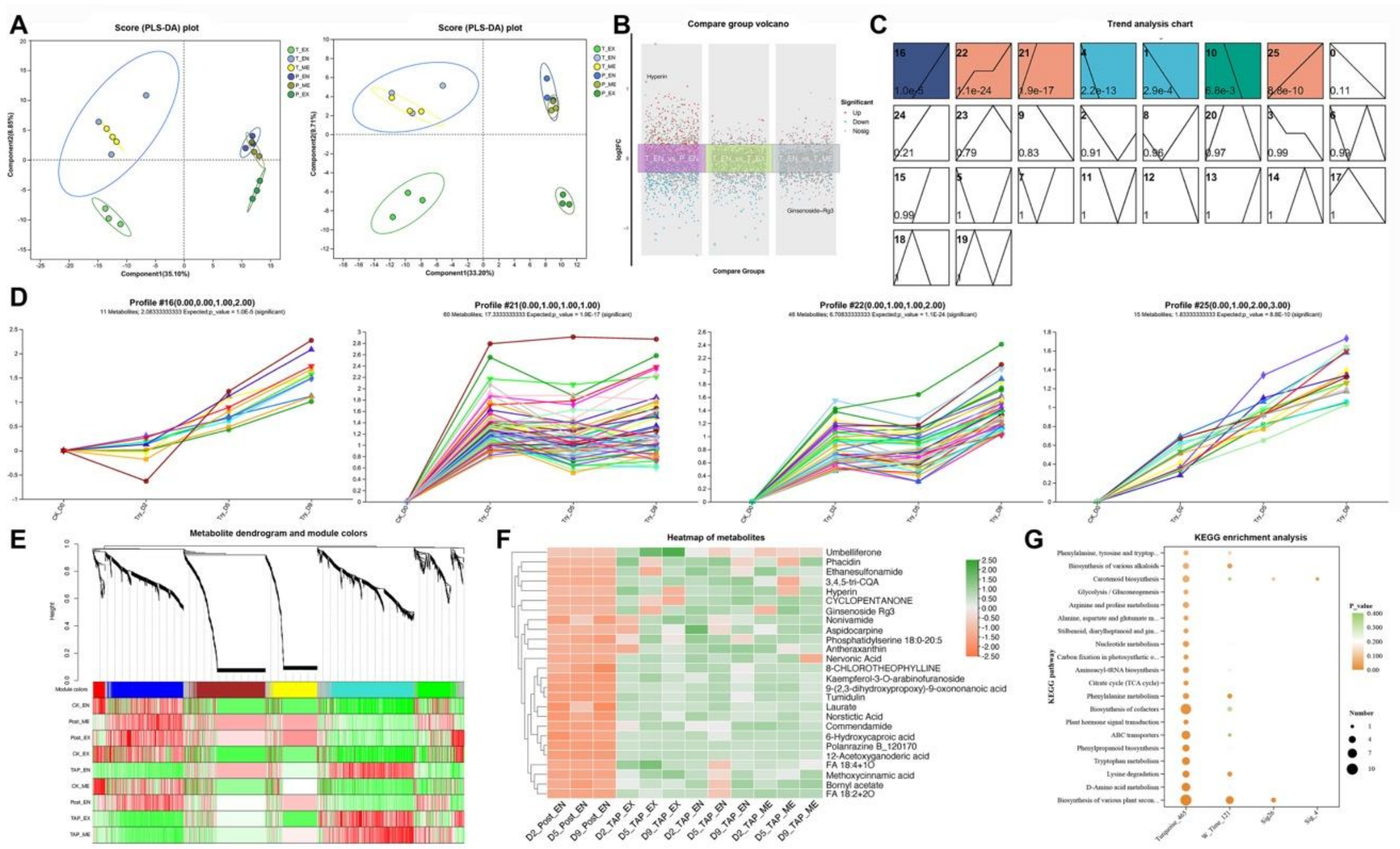

Figure S6: Metabolomics analysis of the pericarp of *H. undatus*

(A) PLS-DA analysis of metabolites identified in three components of Post samples (P_EX, P_EN, and P_ME) and TAP samples (T_EX, T_EN, and T_ME) under positive and negative ion modes, respectively.

(B) A differential pairwise comparison volcano plot displayed the distribution of characteristic metabolites with significant differences among the three groups.

(C) Temporal expression trend analysis of metabolites in each component using the STEM algorithm. Each profile represented a set of metabolites with similar trends, shown as a rectangle. The line within each rectangle depicted the temporal expression trend, while the bottom-left corner displayed the corresponding significance level (p value). Profiles with colored trends indicated significant temporal patterns, where profiles of the same color were grouped into a single cluster based on similar trends. Profiles without color indicated non-significant temporal patterns.

(D) K-means clustering analysis of the temporal expression trends of all metabolites within each profile from panel 6c. The lines represented the changes in expression levels over time.

(E) Cluster results and component heatmap of data samples based on WGCNA analysis.

(F) Hierarchical clustering heatmap showed the abundance of the top 26 metabolites, screened using a Venn diagram in Figure 5B, at three time points during storage.

(G) KEGG enrichment analysis showed the pathways of top 26 metabolites.

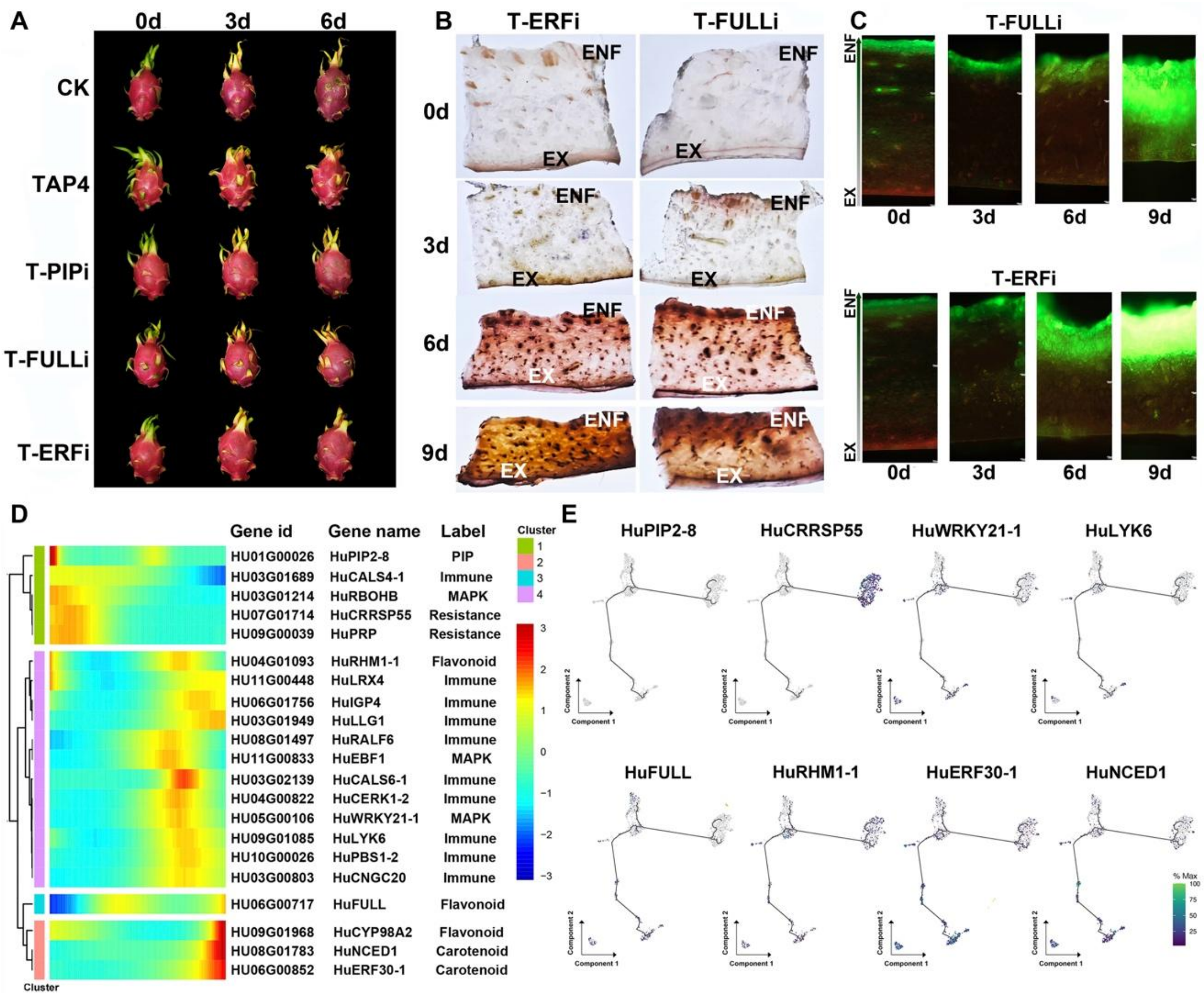

Figure S7: Effects of silencing key genes on fruit phenotype and related gene expression in *H. undatus*

(A-C) Changes in fruit phenotype (A), DAB staining of H_2O_2, and ROS localization shown by H2DCFDA probe (C) after silencing the hub genes

(D) Pseudotime heatmap showing the expression patterns of key genes.

(E) Expression of key genes mapped onto the pseudotime trajectory.

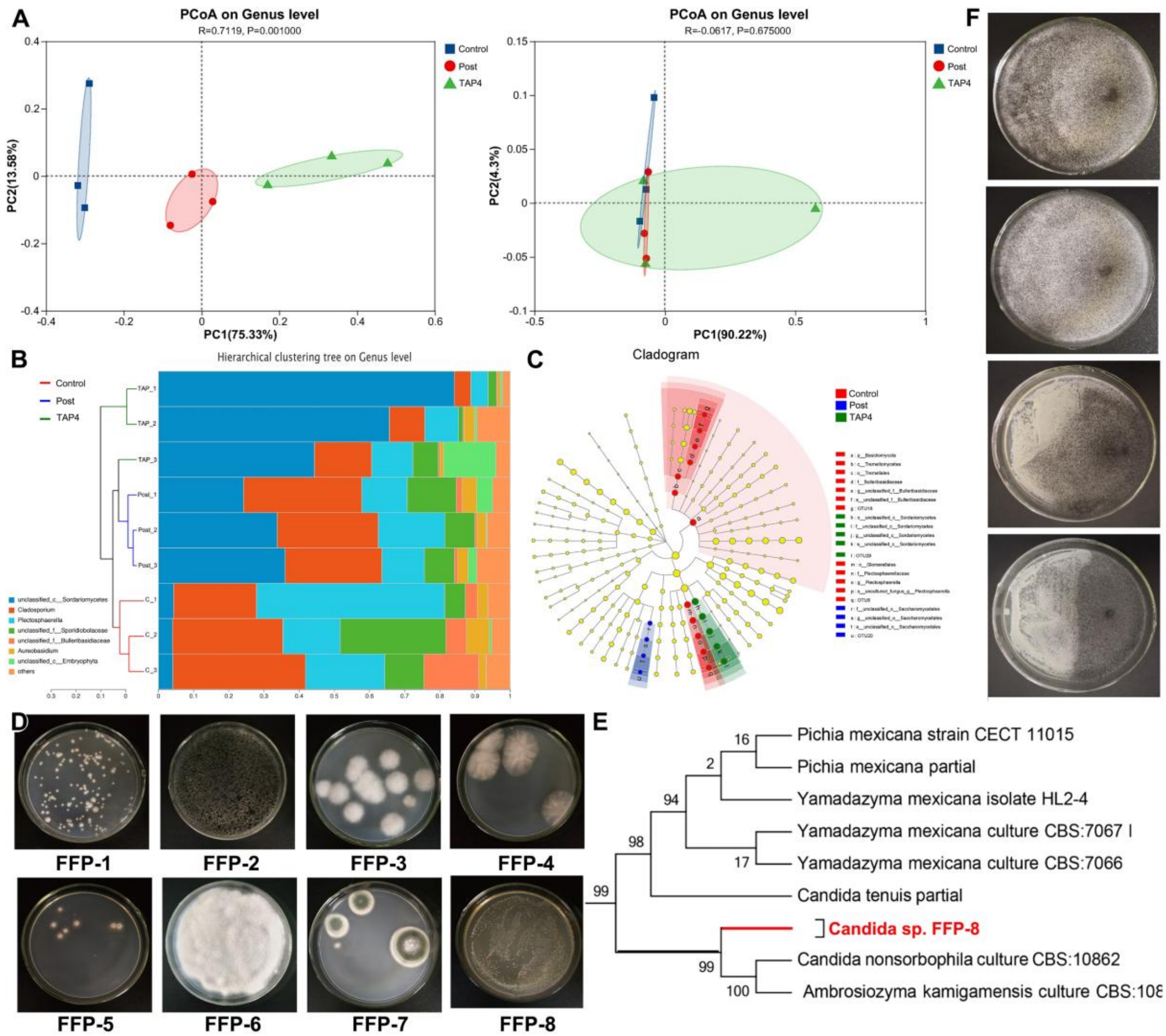

Figure S8: Effects of TAP4 treatment on the postharvest microbial diversity of *H. undatus* fruit

(A) PCoA analysis of fungal (left) and bacterial (right) community composition at the genus level.

(B) Beta diversity analysis of community composition across samples.

(C) LefSe multi-level taxonomic differential analysis from Kingdom to OUT level.

(D) Eight fungal strains (FFP1-8) isolated and purified from fruit lesions.

(E) Phylogenetic tree showing the taxonomic placement of strain FFP-8.

(F) Plate confrontation assays between yeast and mould. From top to bottom: Mould alone, TAP4 + Mould, Candidias + Mould, TAP4 + Candidias + Mould.
