# Supplementary material for: Single-cell and spatial transcriptomics reveals the spatiotemporal trajectory of the small peptide TAP4 in delaying postharvest fruit senescence
Source: Plant Commun. 2026 Apr 7;7(9):101846. doi: 10.1016/j.xplc.2026.101846 (PMC13589556; doi:10.1016/j.xplc.2026.101846)
Supplement: Supplementary file 2 [file mmc15.pdf]

Plant Communications
Research article

Single-cell and spatial transcriptomics reveals the spatiotemporal trajectory of the small peptide TAP4 in delaying postharvest fruit senescence

Xin Li[1,2,3], Yajing Tian[1], Xu Duan[1], Mingyue Lv[1], Xingfeng Shao[4], Yingying Wei[4], Patrick Mason[2], Ruining Guan[5], Qiyue Wang[5], Xinyue Pang[2,6,*], Guoqiang Zhang[7,8,*] and Robert J. Henry[2,9,*]

[1]College of Food and Bioengineering, Henan University of Science and Technology, Luoyang 471023, China

[2]Queensland Alliance for Agriculture & Food Innovation, Queensland Biosciences Precinct, The University of Queensland, St Lucia, Brisbane QLD 4072, Australia

[3]The National and Local Joint Engineering Laboratory of High Efficiency and Superior-Quality Cultivation and Fruit Deep Processing Technology of Characteristic Fruit Trees in South Xinjiang, Tarim University, Alar 843300, China

[4]Zhejiang-Malaysia Joint Research Laboratory for Agricultural Product Processing and Nutrition, College of Food Science and Engineering, Ningbo University, Ningbo 315800, China

[5]School of Pharmacy, Lanzhou University, Lanzhou 730000, China

[6]College of Medical Technology and Engineering, Henan University of Science and Technology, Luoyang 471023, China

[7]Wuhu Green Food Industrial Research Institute Co., Ltd, Wuhu 241000, China

[8]College of Biological and Food Engineering, Anhui Polytechnic University, Wuhu 241000, China

[9]VinUni Big Data Research Institute, VinUniversity, Hanoi, Vietnam

***Correspondence: Xinyue Pang (pangxy78@haust.edu.cn), Guoqiang Zhang (guoqiang2008@163.com), Robert J. Henry (robert.henry@uq.edu.au)**

https://doi.org/10.1016/j.xplc.2026.101846

ABSTRACT

Trypsin (Try) has been reported to possess specific superoxide anion–scavenging activity and to exert pronounced anti-senescence effects in *Hylocereus undatus* fruit; however, the mechanisms underlying its anti-senescence function remain unclear. This study identified TAP4 as the most bioactive peptide among Try autolysis peptides (TAPs). Single-cell transcriptomic analysis revealed that TAP4 alters pericarp cell differentiation trajectories during senescence, particularly in endocarp (EN) cells. It activates the transcription factor (TF) *HuWRKY21-1*, specifically inducing immune responses in inner pericarp cells and upregulating 44 immune-related genes, including *HuIGP4* and *HuLYK6*. Downstream factors, such as HuFULL and ethylene response factors (ERFs), promote the biosynthesis of antioxidants, such as antheraxanthin and hyperin. Functional validation via virus-induced gene silencing (VIGS), callose deposition, disease resistance assays, and quantitative reverse-transcription PCR confirmed the roles of key TFs, including *HuWRKY21-1*, *HuFULL*, and *HuERF30-1*, in immune activation and resistance reconstruction. This work provides evidence for the potential of the EN as a defensive tissue in fruit and opens new avenues for understanding the mechanisms underlying the establishment of plant resistance.

Key words: autolysis peptide, cellular heterogeneity, immune response, omics, pericarp, single-cell RNA-seq

Li X., Tian Y., Duan X., Lv M., Shao X., Wei Y., Mason P., Guan R., Wang Q., Pang X., Zhang G., and Henry R.J. (2026). Single-cell and spatial transcriptomics reveals the spatiotemporal trajectory of the small peptide TAP4 in delaying postharvest fruit senescence. Plant Comm. **7**, 101846.

INTRODUCTION

Senescence has long been one of the core issues in the life sciences. In both animal and plant cells, the induction of cellular resistance is an effective strategy for combating senescence (Terekhova et al., 2023). Plant immune inducers can activate the plant immune system, thereby enhancing abiotic stress tolerance and disease resistance. As a result, they have been widely applied in disease and pest control, stress tolerance improvement, and the promotion of plant growth (Guo and Cheng, 2022; He et al., 2024; Zhu et al., 2024).

The plant innate immune system is mainly divided into two types: pattern-triggered immunity (PTI) and effector-triggered immunity (ETI) (Jones and Dangl, 2006; Ngou et al., 2021). Although these two mechanisms perceive signals differently, their signaling pathways are intricately intertwined, forming an interactive defense system (Ngou et al., 2021; Ortiz-Morea et al., 2022).

 © 2026 The Authors. Published by Elsevier Inc. on behalf of CAS Center for Excellence in Molecular Plant Sciences, Chinese Academy of Sciences, and Chinese Society for Plant Biology.
This is an open access article under the CC BY license (http://creativecommons.org/licenses/by/4.0/).

During both PTI and ETI, reactive oxygen species (ROS) are produced by NADPH oxidases encoded by *respiratory burst oxidase homolog* (*RBOH*) genes, serving as a common and important immune response (Ngou et al., 2021). ROS, which have both physiological and signaling functions, play an essential role in regulating senescence (Guo and Cheng, 2022; Li et al., 2024c). Overexpression of key genes in the cellular antioxidant system enhances antioxidant capacity, reduces endogenous ROS levels, and effectively inhibits senescence (Nambeesan et al., 2010; Wang et al., 2024a). Comparative analyses of different tomato varieties have shown that those with stronger antioxidant capacity exhibit longer postharvest shelf life (Zhang et al., 2013).

Trypsin (Try) is a serine protease, and our research group has revealed its novel activity in scavenging superoxide anions (Li et al., 2017; 2018). Based on this activity, Try's remarkable resistance-inducing effects, independent of its proteolytic activity, have been confirmed in various fruits (Li et al., 2021a; Wang et al., 2023a; 2023b). Experimental evidence has shown that Try can induce a series of immune responses in *Hylocereus undatus* (*H. undatus)* fruit, including stomatal closure, callose accumulation, and ROS bursts. In addition, Try enhances fruit resistance, including tolerance to abiotic stresses and disease resistance (Li et al., 2021b; Pang et al., 2021). The role of Try in strengthening the cell wall of *H. undatus* fruit has also been reported (Zheng et al., 2021). These findings suggest that manipulating the novel immune inducer Try could serve as an important strategy for enhancing fruit resistance and delaying fruit senescence (Mathiazhagan et al., 2021; Zheng et al., 2021). Given its pronounced autolytic behavior, the resulting autolyzed peptides may underlie Try's anti-senescence activity; however, this hypothesis requires experimental validation. Although autolyzed peptides of Try (TAPs) have been previously reported (Maroux and Desnuelle, 1969; Szenthe et al., 2005; Heissel et al., 2019), the specific peptide sequences responsible for scavenging superoxide anions and their mechanisms of action remain to be elucidated.

Peptides, as fragments of proteins, perform various biological functions. They act as signaling molecules and interfere with protein–protein interactions, playing indispensable roles in biological processes across all domains of life (Apostolopoulos et al., 2021). Peptides have emerged as fascinating molecules with nearly unlimited potential. Bioactive peptides have long been recognized and identified in both plant and animal sources. Numerous *in vivo* and *in vitro* studies have demonstrated their broad biological activities in immunomodulation, anti-cancer, and anti-senescence processes (Khavinson et al., 2021). Various peptides function endogenously in plants as defensive compounds, with cysteine-rich peptides playing significant roles as antimicrobial peptides and defensins (Chekan et al., 2024). Many plant peptides are integral to innate immune responses, protecting against fungal and bacterial pathogens as well as insect invasion (Jennings et al., 2001; Mir Drikvand et al., 2024).

With advances in plant immune mechanism research, various plant inducers, including proteins such as PeaT1 and Hrip1, as well as oligosaccharides, have been reported, and their molecular mechanisms have been extensively studied (Klarzynski et al., 2000; Liu et al., 2012; Guo and Cheng, 2022; He et al., 2024). Unlike animal cells, plants lack a circulatory immune system. It has long been believed that plant cells rely on the ability of each cell to initiate innate immune responses against potential pathogenic microorganisms (Couto and Zipfel, 2016; Ngou et al., 2021). However, with the development of single-cell technologies, increasing cellular heterogeneity has been revealed. The application of single-cell transcriptomics has been widespread in animal cells and biomedical research (Van de Sande et al., 2023; Huang et al., 2024). Single-cell studies have revealed the existence of PRIMER cells in plant leaves, which contribute to plant immune responses (Nobori et al., 2025). However, PRIMER cells mediate intrinsic plant immune responses. It remains unclear whether immune responses in fruit are similarly executed by specialized cell populations, as in leaves, or whether induced resistance (elicited by resistance inducers) and constitutive immunity are governed by different mechanisms.

Previous work by our group utilized single-cell transcriptomics technology to reveal the mechanisms of postharvest senescence in fruit (Li et al., 2024a). Resistance processes occurring in the exocarp (EX) were also explored throughout the entire senescence process, including the formation of biotic and abiotic stress resistance (Pang et al., 2024). Although the resistance-inducing and anti-senescence effects of Try have been recognized, the specific cell types responsible for the function of TAPs at the cellular level remain an intriguing question. Although the mechanisms of cellular senescence have been extensively studied (Höhn et al., 2017; Li et al., 2024b; 2024c), those underlying anti-senescence at the single-cell level have yet to be elucidated. Plant immune responses have been extensively studied; however, identifying which cells are responsible for these immune reactions and whether functional heterogeneity exists among different cell types remain novel and important topics. It is therefore crucial to explore the mechanisms by which immune inducers trigger plant resistance and exert anti-senescence effects from a single-cell perspective.

In this study, we first conducted sequence identification, activity assays, and artificial synthesis of the key peptide segment TAP4 derived from TAPs. After phenotypic characterization, we employed single-cell RNA sequencing and spatial transcriptomics RNA sequencing (scRNA-seq and stRNA-seq, respectively) to analyze the pericarp tissues of *H. undatus* subjected to TAP4 treatment. By leveraging four different algorithms, we integrated the datasets to identify cell clusters within the scRNA-seq data. This enabled us to characterize heterogeneous cell populations and uncover the regulatory effects of TAP4 on specific cell types. *In situ* hybridization was used to validate the spatial expression of two hub genes. Furthermore, we performed subpopulation analyses and pseudotime trajectory mapping for the three most affected cell types (EX, mesocarp [ME], and EN fiber [ENF]) and then explored metabolic variations across tissue components. These analyses revealed dynamic changes in pseudotime-associated gene expression, shedding light on the mechanisms underlying the anti-senescence properties of TAP4. Finally, we used the SCODE algorithm to construct a regulatory network based on single-cell pseudotime data and identified pivotal genes within the network using CytoHubba plugins. The functional roles of these key genes in the anti-senescence mechanism of TAP4 were confirmed through virus-induced gene silencing (VIGS) and quantitative reverse transcription PCR (RT–qPCR).

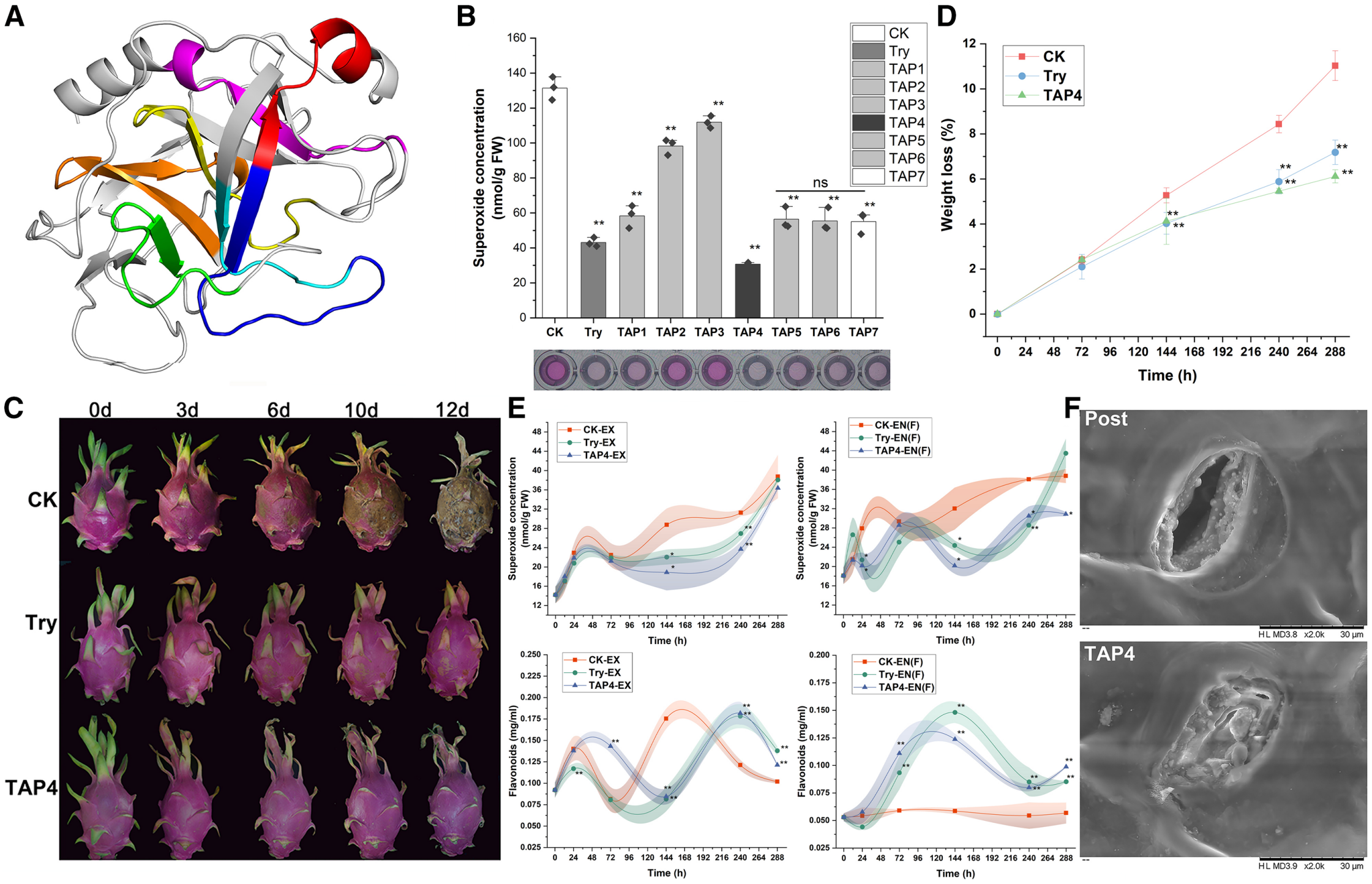

Figure 1. Functional analysis of Try autoproteolytic peptides.

(A) Spatial locations of the autoproteolytic peptide segments of trypsin (Try). Different colors represent the seven peptide segments: TAP1, green; TAP2, orange; TAP3, light blue; TAP4, blue; TAP5, red; TAP6, yellow; and TAP7, purple.

(B) Superoxide anion-scavenging activity of Try and the seven autoproteolytic peptides. Top: bar graph; bottom: photographs of 96-well plates after superoxide determination for each sample.

(C–E) Phenotypic changes **(C)**, weight loss rate curves **(D)**, and changes in superoxide anion and flavonoid levels **(E)** following treatment of *H. undatus* with Try and TAP4. Fruit without Try or TAP4 was used as the control group. In **(E)**, the top panel shows superoxide anion change curves (left to right: exocarp and endocarp [fiber]), and the bottom panel shows flavonoid change curves (left to right: exocarp and endocarp [fiber]). Data in **(B)**, **(D)**, and **(E)** are presented as mean ± standard deviation (SD). Each result was obtained from three biological replicates. Statistical significance was determined using *t* tests, and * indicates a significant difference between treatments ($p < 0.05$).

(F) Scanning electron microscopy images showing TAP4-induced stomatal closure and callose accumulation within stomata.

The expression patterns of upstream immune-related genes induced by TAP4 were also investigated.

This study explored the mechanism by which the antioxidant peptide TAP4 activates fruit immune responses to induce resistance and delay fruit senescence, with a particular focus on the differentiation trajectory of EN cells during plant resistance reconstruction. Our research demonstrated that, in addition to traditional resistance formation in the EX, deeper EN cells also serve as a potential defense line in plants. This study provides new insights into the mechanisms underlying plant immune responses and holds significant application potential for combating senescence.

RESULTS

Anti-senescence activity of trypsin autolytic peptide TAP4

Try exhibited significant superoxide anion scavenging activity, which increased by 56% upon boiling (Supplemental Figure 1 and Supplemental Data 1). Considering the autolysis of Try, we investigated why thermal denaturation did not reduce its superoxide anion scavenging activity. TAPs were extracted, reduced, alkylated, and purified, and their sequences were identified using liquid chromatography–tandem mass spectrometry (LC–MS/MS) (Supplemental Data 2). By aligning these sequences with Try and evaluating the −10LgP and area values, the top seven peptides were selected (Figure 1A). The identified sequences contained diverse amino acids, including hydrophobic, acidic, and basic residues, as well as serine and cysteine, as summarized in Supplemental Data 2. The seven peptides were chemically synthesized (purity > 95%). The superoxide anion scavenging activity of these peptides was measured, revealing that TAP4 exhibited higher activity than Try, whereas TAP2 and TAP3 showed almost no activity, and the remaining peptides displayed weaker activity (Figure 1B). Therefore, TAP4 was selected for subsequent experiments.

During storage of *H. undatus* fruit, the control group gradually exhibited quality deterioration, including color fading, scale desiccation, and lesion formation. In contrast, fruit treated with Try maintained good quality, with a vivid pericarp color, a smooth surface, and minimal lesions. Similarly, fruit treated with TAP4 showed a comparable preservation effect, maintaining excellent condition throughout the storage period (Figure 1C). Weight loss curves indicated that both Try and TAP4 treatments significantly reduced fruit weight loss during storage (Figure 1D). Consistent

with their strong superoxide anion scavenging activities, both Try and TAP4 significantly reduced superoxide anion concentrations in the EX and EN during storage (Figure 1E, upper). Flavonoid biosynthesis serves as a critical indicator of postharvest fruit quality, as it helps maintain quality by delaying cellular membrane lipid peroxidation and cell wall degradation through its antioxidant activity during fruit senescence. The effects of Try and TAP4 on flavonoid biosynthesis in the pericarp layers differed. In the EX, Try delayed the second peak of flavonoid biosynthesis, whereas TAP4 not only delayed the second peak but also markedly enhanced the first peak in the flavonoid biosynthesis curve (Figure 1E, lower left). In the EN, both Try and TAP4 significantly induced flavonoid biosynthesis, with TAP4 showing a more pronounced induction at the early stage (24 h) (Figure 1E, lower right). Meanwhile, scanning electron microscopy revealed that TAP4 treatment induced widespread stomatal closure (Figure 1F). The proportion of closed stomata increased from 68.4% in the post samples to 83.6% in the TAP samples. Callose deposition increased significantly as early as day 3 of storage. In the ME, callose accumulation increased from 962.1 ± 34.1 in the post group to 5173.6 ± 20.1 in the TAP group. In the EN, the level of callose increased from 3688.1 ± 126.6 in the post group to 10,497.7 ± 223.6 in the TAP group. Notably, callose accumulation was observed within stomata (Supplemental Data 1). The expression level of the callose synthase-encoding gene *HuCALS6-1* (HU03G02139) in TAP-treated samples (0.02066) was higher in the EX than in post-treatment samples (0.001804) (Supplemental Data 3).

Generation of a single-cell atlas for *H. undatus* pericarp

To systematically uncover the gene expression patterns involved in TAP4-mediated delay of *H. undatus* senescence, we isolated pericarp cells from mature (CK group), senescent (post group), and TAP4-treated (TAP4 group) fruit and conducted single-cell sequencing (Figure 2A). More than 5000 cells were captured from each group. After filtering out doublets, 5656, 7670, and 5571 cells from the CK, post, and TAP4 groups, respectively, were included in the analysis. Of the approximately four million reads from the pericarp samples, 95.4%, 91.8%, and 78.3% were mapped to the *H. undatus* genome. The expression of a median of 708, 777, and 693 genes was detected in the CK, post, and TAP4 samples, respectively (Supplemental Data 3).

To create a comprehensive single-cell atlas of TAP4-mediated anti-senescence effects in the pericarp, data from the three groups were integrated for cell clustering and annotation. A total of 27,735 genes were identified from the single-cell transcriptomic data and grouped into 26 distinct cell clusters. Uniform manifold approximation and projection (UMAP) was employed to visualize and explore these clusters (Figure 2B and Supplemental Data 4).

To annotate each cell cluster, we identified genes enriched in each cluster, defined as those with significantly higher expression in each cluster compared with all other clusters. A total of 5343 upregulated genes were identified (Supplemental Data 5). From this set, the top 3 most specifically expressed genes in each cluster, yielding 70 marker genes, were selected for further analysis (Figure 2C).

Gene expression specificity varied among clusters, even among marker genes. Some marker genes showed highly cluster-specific expression. For example, the marker genes HU09G00039, HU08G01941, and HU07G01714 in cluster 15, as well as HU03G02607, HU03G02609, and HU03G02606 in cluster 19, exhibited strong cluster-specific expression. On the other hand, some marker genes were highly expressed across multiple clusters. For instance, HU11G01114 and HU09G01127 from cluster 0 also showed high expression in cluster 7, whereas HU06G01840 was highly expressed in clusters 0, 7, and 3. Similarly, HU02G03369 from cluster 1 was highly expressed in clusters 4, 5, and 19 (Figure 2C). Violin plots more clearly illustrated the expression specificity of these genes across clusters (Supplemental Figure 2). The widespread co-expression of even highly specific marker genes across multiple cell clusters suggests that these clusters likely belong to the same cell type, providing supporting evidence for subsequent cell type annotation.

Correlation analysis revealed high correlations among clusters 3, 7, 8, and 14, with particularly strong correlations between clusters 3 and 7 and between clusters 3 and 8, all with correlation coefficients greater than 0.92 (Figure 2D). Additionally, high levels of correlation were observed among clusters 6, 9, 10, 11, 13, 17, and 18. Clusters 24 and 25 exhibited low correlations with other clusters, indicating a high degree of independence (Supplemental Data 6). These inter-cluster correlations provide additional support for refining cell type identification.

Spatial transcriptomics and cell type identification in *H. undatus* pericarp

To identify the 26 cell clusters obtained from single-cell transcriptomic data, we performed spatial transcriptomics analysis using the 10× Genomics Visium platform. Spatial transcriptomic sequencing yielded 3566 valid spots, with 98.0% valid barcodes. The median number of genes per spot was 1435. Four algorithms for single-cell identification—single cell recognition (SingleR), single cell identificator based on E-test (SciBet), regional cell type deconvolution (RCTD), and cell annotation in regional decomposition (CARD)—were used to associate the scRNA-seq and spatial transcriptomics results.

Based on the results from SingleR and SciBet, cell clusters 3, 7, 8, 14, and 20 were classified as ME, with cluster 14 potentially corresponding to EX or ENF. Clusters 1, 2, 4, 5, 15, and 19 were classified as EX by both methods, with cluster 5 potentially corresponding to ENF. Both methods also assigned clusters 12 and 24 to spatially resolved vascular bundle (VB) and EN, respectively. The remaining cell clusters were categorized as ENF (Figure 2E, upper).

The results from RCTD and CARD were largely consistent with those from SingleR and SciBet, providing further evidence for the classification of some of the ambiguous cell clusters identified earlier. Cluster 5 should be classified as EX rather than ENF, and cluster 14 should be classified as ME. Furthermore, cluster 0 should be assigned to ME (Figure 2E, lower; Figure 3). Cluster correlation analysis also supported that clusters 0 and 14 belong to the same group as clusters 3, 7, and 8, namely the ME group (Figure 2D).

Based on the results from the four algorithms, and considering the proximity of similar cell clusters on the UMAP plot, as well as gene

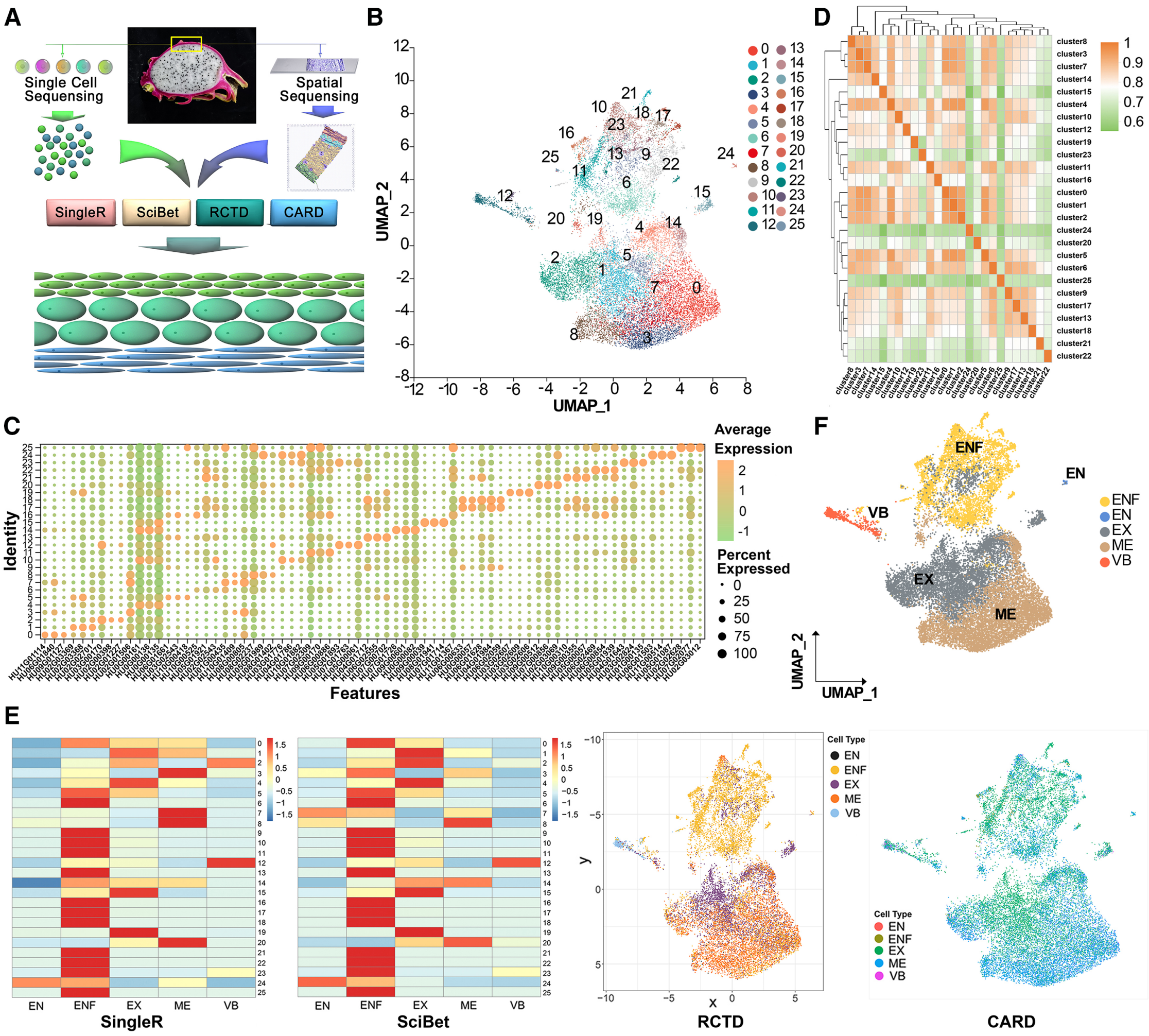

Figure 2. Generation of an *H. undatus* pericarp cell atlas.

(A) Flowchart of the experimental design for scRNA-seq and stRNA-seq in this study.

(B) UMAP visualization of 26 cell clusters in the CK, post, and TAP groups of *H. undatus* pericarp samples. Each dot represents a single cell, and colors denote the corresponding cell clusters.

(C) Expression patterns of representative cluster-specific marker genes on UMAP. Dot size indicates the proportion of cells in a cluster expressing a given gene, and the color scale represents gene expression levels, with orange indicating high expression and green indicating low expression.

(D) Correlation heatmap of the 26 clusters obtained from scRNA-seq data.

(E) Correlation analysis between scRNA-seq and stRNA-seq using four algorithms: SingleR, SciBet, RCTD, and CARD.

(F) UMAP plot showing the final classification of five cell types based on marker genes from the 26 clusters of single cells after manual annotation. ENF, endocarp fiber; EN, endocarp; EX, exocarp; ME, mesocarp; and VB, vascular bundle.

co-expression across multiple clusters shown in the violin plots (Figure 2 and Supplemental Figure 3), the 26 cell clusters identified from the scRNA-seq data were classified into five distinct cell types. Clusters 0, 3, 7, 8, 14, and 20 were categorized as ME cells. Clusters 1, 2, 4, 5, 15, and 19 were designated as EX cells. Cluster 12 was identified as VB cells, and cluster 24 as EN cells. Clusters 6, 9, 10, 11, 13, 16, 17, 18, 21, 22, 23, and 25 were classified as ENF cells (Figure 2F). Through integrated analysis of stRNA-seq and scRNA-seq data, the identification of *H. undatus* pericarp cell types was achieved, resulting in the construction of a comprehensive cell atlas of the pericarp (Figure 2F).

Impact of TAP4 on cell clusters in *H. undatus*

Statistical analysis of cell type abundance across the CK, post, and TAP4 treatment groups revealed notable alterations in pericarp cell composition following storage-induced senescence and TAP4 application (Supplemental Data 4). The proportion of cells in the EX component declined markedly (53.47% in the CK group and 29.91% in the post group) and was further reduced to 16.62% in the TAP4 group (Figure 3A). In contrast, the proportion of ME cells—the major component accounting for 34.91% of the total cells—increased from 16.64% in the CK group to 62.54% in the post group and then decreased to 20.82% in the TAP4 group (Figure 3A). Another component, ENF, which makes up 25.05% of the total cells, showed a trend opposite to that of EX. The proportion of ENF cells decreased from 20.88% in the CK group to 7.21% in the post group and increased to 71.91% in the TAP4 group (Figure 3A). These findings were further corroborated by UMAP visualization, which clearly illustrated alterations in each cell cluster (Figure 3B).

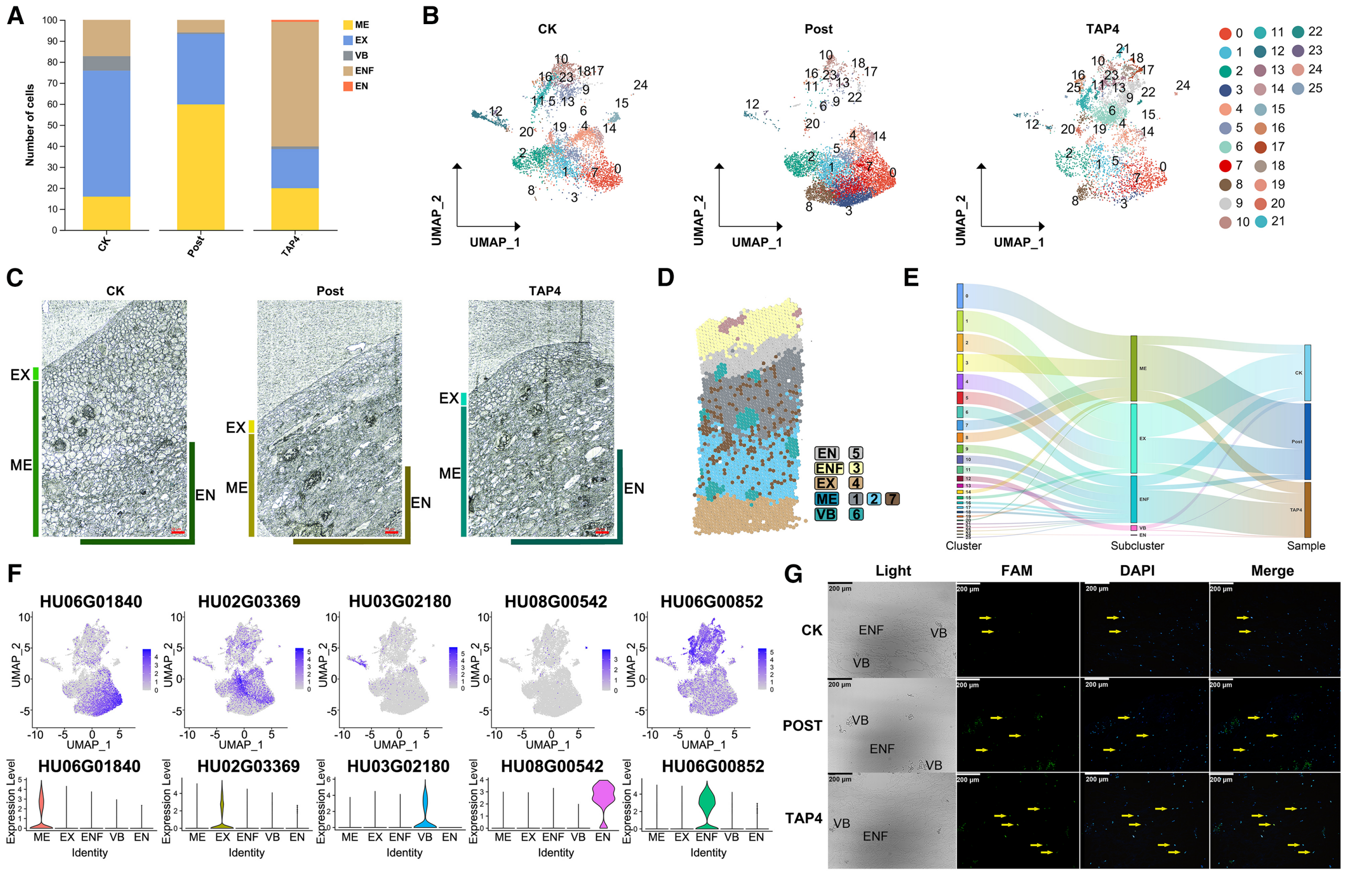

Figure 3. Analysis of key components and marker genes during the transition from maturity to senescence.
(A and B) Stacked bar charts **(A)** and UMAP plots **(B)** showing the proportions of cell types in CK, post, and TAP samples.
(C) Optical microscopy images of CK, post, and TAP samples.
(D) Spatial transcriptomics-based visualization of cell types. Clusters are named according to their spatial positions. Scale bars, 1000 μm.
(E) Sankey diagram showing the distribution of clusters into components in CK, post, and TAP samples.
(F) UMAP and violin plots showing the expression profiles of the top marker genes for each of the five components.
(G) RNA-FISH showing the predominant localization of *HuSAG12* in the ENF component. *HuSAG12* probes were labeled with FAM (green), and nuclei were stained with DAPI (blue). Scale bars, 100×10^{-6} μm.

Microscopic images revealed changes in cell types within the pericarp of *H*. *undatus* across the CK, post, and TAP4 groups. In the CK samples, three to four well-organized layers of EX cells were neatly arranged. Moving inward, the ME region consisted of enlarged parenchyma cells with tight intercellular spaces. Further inward, cells in the EN layer were larger in volume and arranged in an orderly manner (Figure 3C, leftmost panel). In the post samples, the cuticle layer became thinner and exhibited prominent folds (Figure 3C, middle panel). The adjacent EX cell layers exhibited increased intercellular spaces, and the arrangement of EX cells was significantly looser than in the CK samples. The ME cell layer became markedly thinner, with only a small region (approximately two to three cell layers) of parenchyma cells retaining their enlarged morphology. The remaining ME cells displayed pronounced dehydration, along with evident shrinkage and folding. Further inward, the EN cell layer also experienced significant dehydration, with cells becoming smaller, more disorganized, and showing markedly increased intercellular spaces (Figure 3C, middle panel). These cellular changes observed in the post group were notably alleviated in the TAP4 group. The arrangement of EX cells was less disrupted, and dehydration in the ME and ENF layers was significantly mitigated. Cells maintained a morphology similar to that of the CK group (Figure 3C, rightmost panel and Supplemental Data 1). The cell types identified by spatial transcriptomics displayed spatial arrangements consistent with those observed in the microscopic images (Figure 3D).

To thoroughly illustrate the relationship between the 26 clusters identified by scRNA-seq and their distribution across the three sample groups, a Sankey diagram was generated (Figure 3E). Based on the UMAP overview of the 26 clusters across the three sample groups, the spatial expression patterns of the top genes for each of the five components are shown in Figure 3F. Specifically, Hu06G01840, Hu02G03369, Hu06G00852, Hu08G00542, and Hu03G02180 exhibited strong expression specificity in the ME, EX, ENF, EN, and VB components, respectively. Violin plots clearly demonstrate the high expression of these marker genes within each component (Figure 3F). These marker genes showed significant differences in expression patterns among the CK, post, and TAP4 samples. The ME marker gene Hu06G01840 displayed extremely low expression in CK samples, was significantly upregulated in post samples, and was markedly reduced in TAP4 samples compared with

post samples (Supplemental Figures 4A and 4B). The expression pattern of the VB marker gene Hu03G02180 was similar to that of Hu06G01840 in the ME. The EX marker gene Hu02G03369 showed the highest expression in CK samples, was downregulated in post samples, and partially recovered in TAP4 samples (Supplemental Figure 4F). The ENF marker gene Hu06G00852 showed significantly higher expression in TAP4 samples than in CK and post samples. Similarly, the expression pattern of the EN marker gene Hu08G00542 was comparable to that of the ENF marker gene Hu06G00852 (Supplemental Figures 4A, 4B, 4D, and 4E).

These findings were further validated by RNA fluorescence *in situ* hybridization (RNA-FISH) experiments. *Senescence-associated gene 12 (SAG12*) is widely recognized as a senescence marker gene (James et al., 2018; Myat et al., 2022). Our previous results demonstrated that *HuSAG12* (Hu08G02237) is expressed exclusively in the ME cells of both CK and post samples, identifying it as a marker gene for the ME (Li et al., 2024a). Our RNA-FISH results showed that *HuSAG12* was almost undetectable in the ENF cells of CK and post samples, consistent with earlier findings. However, unlike in the normally senescent post samples, *HuSAG12* exhibited high expression not only in the ME but also in the ENF cells of TAP4 samples (Figure 3G and Supplemental Data 1).

Similarly, the ENF marker gene *HuERF30-1* (Hu06G00852) showed significantly increased expression in TAP4 samples, consistent with the single-cell sequencing results (Supplemental Figure 4C). In the scRNA-seq data, the relative expression values of *HuSAG12* in the CK, post, and TAP4 samples were 0.0395, 2.395, and 3.723, respectively. For *HuERF30-1*, the corresponding values were 0.572, 0.372, and 1.880. The combined results from RNA-FISH and single-cell transcriptomics confirm the specific spatial expression patterns of these two key genes in ME and ENF, as well as their dynamic expression changes across different samples (Figure 3 and Supplemental Figure 4).

Effects of TAP4 on the differentiation trajectory of senescence in *H. undatus*

Our previous work reported the spatiotemporal trajectory of senescence in *H. undatus* pericarp (Li et al., 2024a). However, how TAP4 exerts its anti-senescence effects remains to be elucidated. To uncover its mechanism of action in each cell type, we selected EX, ME, and ENF cells from *H. undatus* pericarp that exhibited significant changes during senescence and conducted subgroup and subsequent pseudotime analyses.

EX, ME, and ENF cells were re-clustered, and cells with distinct expression features were grouped into nine subclusters (Supplemental Figure 5A). To characterize expression profiles across the three samples, ratio of global unshifted entropy (ROGUE) was applied to evaluate the purity of these subclusters. In scRNA-seq analyses, ROGUE is an objective, quantitative metric used to assess clustering quality, identify pure cell subtypes, and construct standardized single-cell atlases (Liu et al., 2020). As shown in Supplemental Figure 5B, clusters 5, 6, and 7 exhibited ROGUE values $\geq$ 0.9, indicating high-quality cell populations. Cluster 4 contained only CK-derived cells (Supplemental Data 3), and post-derived cells in cluster 8 also showed a relatively high ROGUE value (0.86) (Supplemental Data 3). Cell clusters with ROGUE values greater than 0.85 were retained, and ultimately, 1595 cells with distinct expression features and high cluster purity were selected from the nine subclusters. A cell set, MXF_1595, was established and subjected to further subgroup analysis. Six cell subgroups were identified: subgroup 0 consisted of CK-derived cells; subgroups 1 and 2 consisted of post-derived cells; subgroups 3 and 4 consisted of TAP4-derived cells; and subgroup 5 contained cells derived evenly from both post and TAP4 samples (Figure 4A).

The differentiation trajectory of the 1595 selected cells across the three samples was inferred, with their arrangement along the trajectory represented by latent time (Figure 4B). These cells were classified into five states (Figure 4C). Cells in state 1, derived from CK samples, exhibited a low degree of differentiation. Cells in states 2, 4, and 5, primarily originating from post samples, were located near branch points 1 and 2. Some of these cells formed small branches along the differentiation trajectory, displaying a higher degree of differentiation than state 1 cells from CK samples (Figure 4C). Cells in state 3, positioned at the terminus of the primary differentiation trajectory and originating from TAP4 samples, exhibited a more open differentiation state (Figures 4B–4D).

When spatial information in the developmental trajectory was considered, these cells displayed a distinct spatial distribution pattern. Tracing the origin of each cell based on its barcode revealed that they belonged to the EX, ME, and ENF components, and mapping them onto the differentiation trajectory indicated a clear progression. The starting point of differentiation was primarily composed of EX cells, followed by ME cells in the middle and ENF cells at the end of the trajectory (Figure 4E).

The embedded heatmap of pseudotime-related genes (PRGs) demonstrated that the top 100 PRGs clustered into four gene groups (Figure 4F). Along the pseudotime axis, genes associated with phenylpropanoid biosynthesis exhibited the earliest expression, followed by the activation of stress-responsive genes. At later stages of pseudotime under TAP4 regulation, stress-responsive genes were substantially upregulated, with hormone pathways—particularly ethylene-related signaling pathways—being prominently activated (Figure 4F).

Among the PRGs, 147 genes were involved in cellular immune responses (Supplemental Data 7). Of these, 44 genes, including callose synthase–encoding genes *HuCALS6-1* (HU03G02139) and *HuCALS4-1* (HU03G01689), *HuPBS1-2* (HU10G00026, serine/threonine-protein kinase PBS1-like), *HuCERK1-2* (HU04G 00822, LysM domain receptor-like kinase 3 [RLK3] isoform X5), *HuLYK6* (HU09G01085, LysM domain RLK3), *HuIGP4* (HU06G 01756, predicted: probable LRR receptor-like serine/threonine protein kinase At1g56140), *HuLLG1* (HU03G01949, GPI-anchored protein LLG1-like), *HuRALF6* (HU08G01497, rapid alkalinization factor-like, RALF), and *HuLRX4* (HU11G00448, leucine-rich repeat extensin-like protein 4), were specifically upregulated in EN cells (Supplemental Figure 5C and Supplemental Data 7).

Additionally, 100 genes exhibiting significant changes along the pseudotime axis at the differentiation branch point were identified,

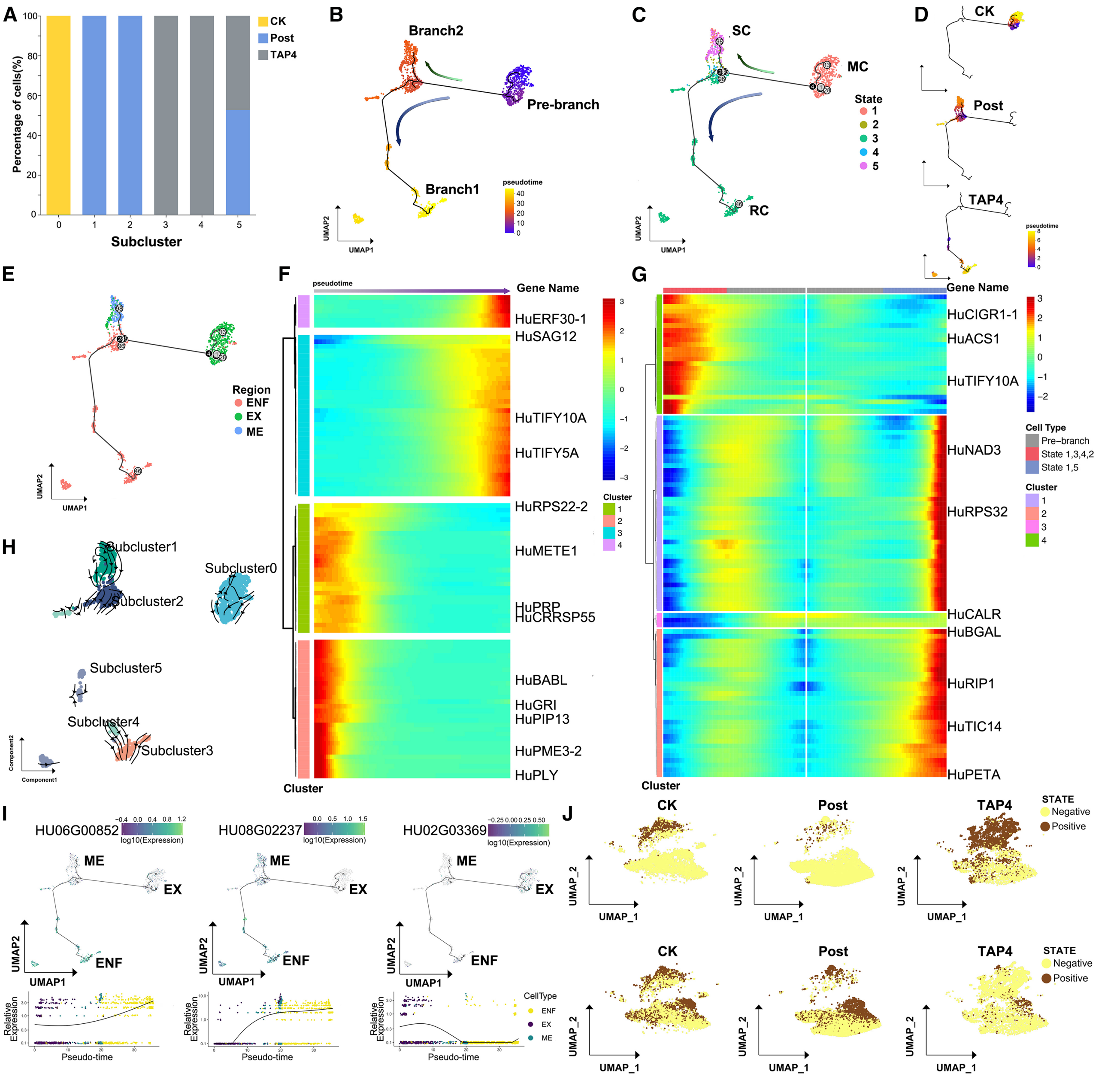

Figure 4. Pseudotime analysis of cells from CK, post, and TAP samples.

(A) Bar charts illustrating the proportions of cells from the three samples within each subcluster of MXF_1595 cells.

(B) Latent time showing the internal clock of cells. Different colors represent different differentiation times, with darker shades of red and blue indicating earlier and later times, respectively.

(C) Five cellular states mapped onto the pseudotime plot.

(D and E) Cells from the three samples **(D)** and three components **(E)** mapped onto the pseudotime trajectory.

(F) Clustering of the top 100 pseudotime-related genes along the pseudotime progression.

(G) Heatmap of the top 100 differentially expressed genes associated with branch point 1 along the pseudotime trajectory.

(H) RNA velocity analysis mapping three cellular components onto the pseudotime plot. Colors represent different cell components. Black arrows indicate potential cell trajectories, and their lengths represent the strength of these trends.

(I) Gene set scoring results showing cells highly correlated with upregulated (top) and downregulated (bottom) genes induced by TAP4 in the three samples. Each point represents a cell. "Positive" indicates cells with high correlation (brown), while "negative" indicates cells with low correlation (yellow).

(J) Visualization of expression patterns of marker genes from the three components mapped onto the pseudotime trajectory. Expression curves for each gene are shown below. Point colors represent different components, and the horizontal axis indicates time progression from left to right.

leading to the formation of two distinct differentiation trajectories. These genes were clustered and visualized, revealing dynamic changes in gene expression modules across cells along the pseudotime differentiation trajectory (Figure 4G). Based on branch point 1, the gene expression patterns of EX, ME, and ENF cells transitioning from the pre-branch stage to branches 1 and 2 were evaluated (Figure 4G). Clustering analysis revealed four expression clusters representing distinct gene differentiation patterns (Figure 4G). Oxidative phosphorylation and RNA polymerase were identified as the major Kyoto Encyclopedia of Genes and Genomes (KEGG) pathways enriched in gene clusters 1 and 2, which were associated with branch 2 and represented by post samples (Figure 4G). These clusters reflect gene expression changes associated with the transition of cells from a mature state to senescence.

Cluster 2 was enriched in genes involved in oxidative phosphorylation, which may contribute to senescence induction. At the end of the differentiation trajectory of branch 1, represented by TAP4 samples, genes in cluster 4 exhibited high expression. These included *HuCCL9* (HU02G01787), which has quercetin 3-O-glucosyltransferase activity, as well as multiple ethylene-responsive and jasmonic acid-related genes, such as *HuERF28* (HU05G01921), *HuERF30-1* (HU06G00852), *HuTIFY5A* (HU08G01785), and *HuTIFY10A* (HU03G02459) (Figure 4G and Supplemental Data 11).

RNA velocity analysis revealed that MCs (mature cells) in CK samples acted as the origin of differentiation, directing differentiation toward SCs (senescent cells) in the ME component of post samples and RCs (immune-resistant cells) in the ENF component of TAP4 samples (Figure 4H). The RNA velocity dynamics also indicated the presence of stable attractor states for various cell types. Under mature conditions, MCs represented a stable attractor state. Moreover, RCs at the end of branch 1 differentiation, induced by TAP4, exhibited an attractor state, whereas SCs in post samples transitioned into an attractor state during the later stages of storage along branch 2 (Figure 4H).

To further clarify the regulatory effects of TAP4 on cells within the ME, EX, and ENF components, TAP4-regulated differentially expressed genes (DEGs) were identified and classified into upregulated and downregulated gene sets. Based on gene set scoring results, cells highly associated with these gene sets were selected. The results clearly showed that upregulated genes, primarily induced by TAP4, exhibited high expression in ENF cells, whereas downregulated genes were highly expressed in the EX cells of CK samples and in the ME cells of senescent post samples (Figure 4I). The pseudotime trajectories of cells highly associated with upregulated and downregulated genes further supported this pattern (Supplemental Figures 5D and 5E). The expression patterns of three marker genes—*HuERF30-1* (HU06G00852), *HuSAG12* (HU08G02237), and *HuACCH4-2* (HU02G03369)—across the ME, EX, and ENF components were also mapped onto the pseudotime trajectory (Figure 4J).

Using single-cell ordinary differentiation equation (SCODE), the gene regulatory network (GRN) associated with TAP4-mediated inhibition of pericarp senescence was inferred from MXF_1595 cells (Supplemental Data 8). By integrating the top 100 dynamically expressed genes along the pseudotime trajectory, we revealed a complex network controlling pericarp senescence (Supplemental Figure 5F). Using the Cytoscape plugin "MCODE," we identified two clusters consisting of 29 and 5 nodes, respectively (Supplemental Data 8). Notably, resistance-related genes in cluster 1, such as *HuCRRSP55* (HU07G01714, cysteine-rich repeat secretory protein 55-like), *HuPRP* (HU09G00039, hypothetical protein SOVF_199290), and *HuPGLR* (HU08G00690, hypothetical protein BVRB_3g059140), were highlighted. Meanwhile, cluster 2 included the senescence marker gene *HuSAG12* and the ethylene response factor-encoding gene *HuERF35* (HU08G02191, ethylene-responsive transcription factor [TF] 4-like) (Supplemental Figure 5F).

To explore the potential regulatory mechanisms underlying the anti-senescence process, we next aimed to identify key hubs within the PRG regulatory network. Investigating interactions among PRGs provides insight into their roles in pericarp cell senescence. The CytoHubba plugin in Cytoscape was used to rank nodes using twelve topological algorithms, including maximum neighborhood component (MNC) and Closeness, to identify core nodes within the GRN (Supplemental Data 8). Among these, eight algorithms yielded nearly identical top 20 nodes, all of which exhibited distinct temporal and spatial expression specificity (Supplemental Data 8).

The third-ranked hub node, *HuPGLR* (HU08G00690, endo-polygalacturonase), was assigned to gene cluster 2, which represented the earliest expressed genes. All genes in cluster 2 were specifically upregulated in cell cluster 15 of the EX component and were exclusively expressed in CK samples (Supplemental Figure 5G and Supplemental Data 8).

The first- and second-ranked hub nodes, *HuCRRSP55* (HU07G 01714, cysteine-rich repeat secretory protein 55-like) and *HuPRP* (HU09G00039, hypothetical protein SOVF_199290), were assigned to cluster 1, which was characterized by early expression in pseudotime. Their expression levels were significantly higher in CK samples than in post and TAP4 samples. Similarly, they were predominantly expressed in the EX component. Specifically, both *HuCRRSP55* and *HuPRP* were upregulated in clusters 2 and 15 of the EX. *HuCRRSP55* was also upregulated in cluster 19 of the EX (Supplemental Figure 5G and Supplemental Data 8).

Regulation of metabolites in *H. undatus* pericarp by TAP4 treatment

To validate the temporal trajectory of fruit senescence identified through pseudotime analysis, metabolite levels in the EX, ME, and ENF components were separately assessed in TAP4-treated and senescent post samples. Partial least-squares discriminant analysis (PLS–DA) revealed significant differences in metabolites within each component between the post and TAP4 groups under both cationic and anionic detection modes (Supplemental Figure 6A).

In TAP4 samples, the ENF component exhibited 356 upregulated metabolites compared with post samples. Similarly, the ME and EX components in TAP4 samples showed 51 and 91 upregulated metabolites, respectively (Supplemental Figure 6B). Among these, the flavonoid compound hyperin (quercetin-3-O-β-D-galactoside) was prominently upregulated in the ENF under TAP4 treatment (Supplemental Figure 6B).

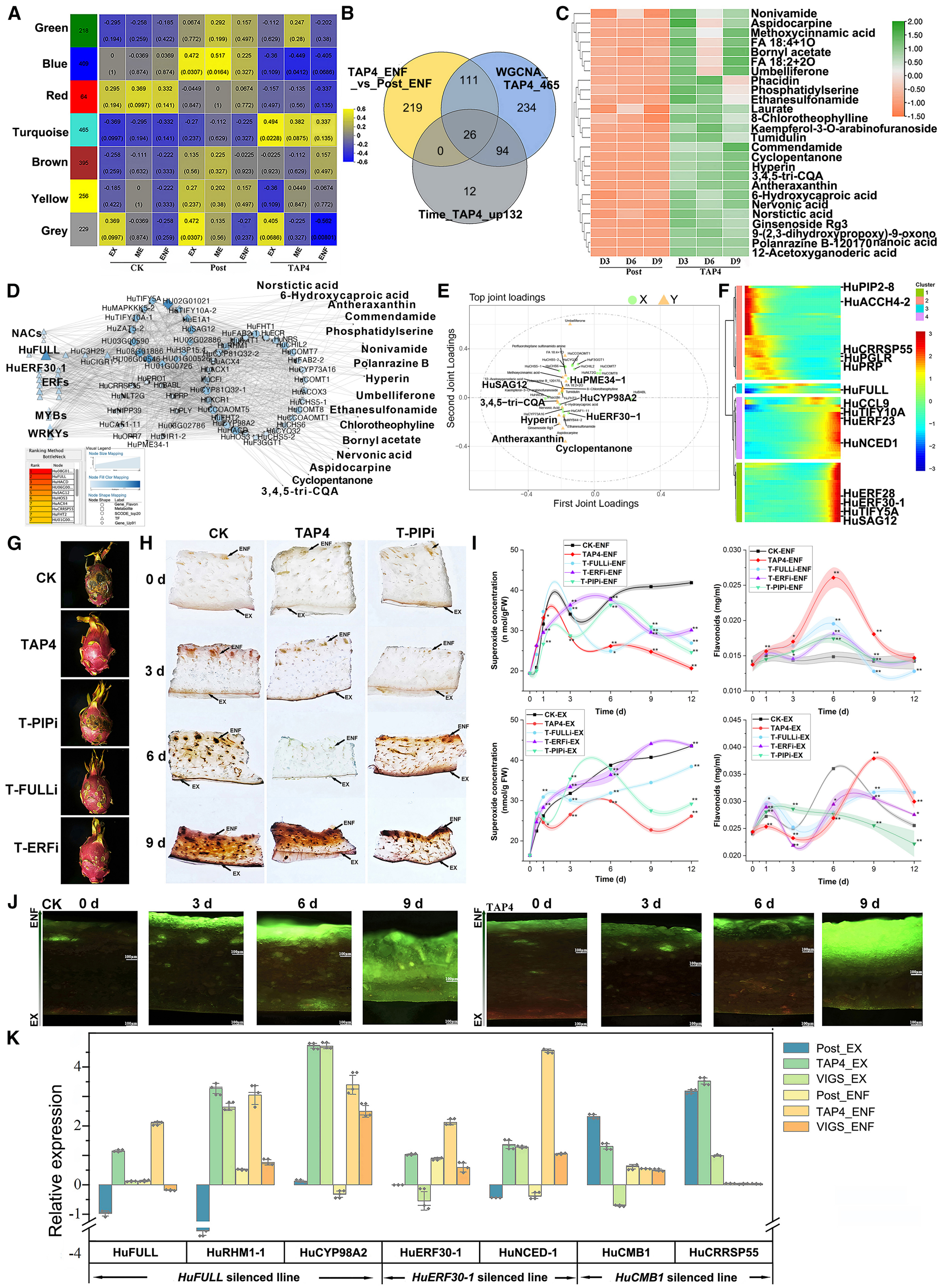

Figure 5. Identification of key metabolites and genes by integrating metabolomics and scRNA-seq.
(A) Correlation between modules and components analyzed by WGCNA.
(B) Venn diagram showing 356 upregulated metabolites in the TAP vs. post groups in the EN component, 465 TAP-regulated metabolites identified by WGCNA, and 132 metabolites identified by the STEM analysis.

(legend continued on next page)

Temporal analysis revealed that, under TAP4 induction, metabolite levels increased significantly over time and were categorized into four clusters comprising a total of 132 metabolites (Supplemental Figures 6C and 6D). Weighted gene co-expression network analysis (WGCNA) of differential metabolites across nine samples (three components—EX, ME, and ENF—from the CK, post, and TAP4 groups) identified seven modules (Supplemental Figure 6E). Among these, the turquoise module, containing 465 metabolites, was highly positively correlated with the TAP4 group (Figure 5A). By conducting a Venn analysis of differential metabolites identified through multiple approaches, 26 TAP4-regulated key metabolites were identified (Figure 5B and Supplemental Data 9). Clustering analysis further showed that these 26 metabolites could be divided into subclusters, with five metabolites—commendamide, cyclopentanone, hyperin, 3,4,5-tricaffeoylquinic acid (3,4,5-tri-CQA), and antheraxanthin—being highly expressed in the ENF component under TAP4 treatment and grouped in subcluster 2 (Figure 5C). Notably, although commendamide levels increased in EN, its levels in the ME component were higher than in EN. Therefore, the remaining four compounds—cyclopentanone, hyperin, 3,4,5-tri-CQA, and antheraxanthin—were considered key metabolites in the ENF component for subsequent analyses (Supplemental Figure 6F and Supplemental Data 9). KEGG enrichment analysis was performed on the 465 metabolites from the turquoise module identified through metabolite screening, the 132 time-series-related metabolites, the 26 key metabolites, and the final four core metabolites. The enriched pathways were visualized using bubble plots (Supplemental Figure 6G). The results showed that phenylpropanoid biosynthesis, together with its downstream flavonoid and carotenoid pathways, represented the principal pathways enriched among these TAP4-regulated metabolites.

In addition, considering that betacyanins are the major sources of pericarp pigmentation in *H*. *undatus*, we examined betacyanin-related compounds and identified three candidates. Among them, deoxycarnitine exhibited significantly higher accumulation in the EX upon TAP4 induction (Supplemental Data 9), which may partly explain the increased pericarp color brightness observed after TAP4 treatment (Figure 1C).

To elucidate the regulatory mechanisms of these metabolites, a co-expression network was constructed linking TAP4-regulated TFs, key genes, and the identified key metabolites (Figure 5D). Given that most of the key metabolites identified were flavonoids, genes involved in flavonoid biosynthesis were incorporated into the network, in addition to the key genes identified through SCODE analysis. *HuFULL* (HU06G00717) emerged as the most significant TF, along with eight AP2 family TFs, including *HuERF23* (HU05G00390) and *HuERF30-1* (HU06G00852), which play crucial roles in regulating metabolites such as hyperin. Furthermore, *HuNCED1* (HU08G01783) was identified as a key gene involved in the biosynthesis of carotenoid-related metabolites, including antheraxanthin (Supplemental Data 10). Orthogonal projections to latent structures (O2PLS) analysis integrating transcriptomic and metabolomic data highlighted hyperin and antheraxanthin as the most critical metabolites. Several ethylene response factor (ERF) genes, including *HuERF30-1*, showed strong correlations with these metabolites, indicating their regulatory roles in biosynthesis and accumulation (Figure 5E).

Expression patterns and functional roles of hub genes in the senescence process

Previous studies have illustrated that H_2O_2 likely serves as a key signaling molecule during pitaya fruit senescence (Li et al., 2024a). The heatmap of hub gene expression revealed that *HuPIP2-8* (HU01G00026), an plasma membrane intrinsic protein (PIP), also named aquaporin, facilitating H_2O_2 signal transduction into cells, was the earliest-expressed gene (Figure 5F). Subsequently, during the early stages of senescence, resistance-associated genes involved in the phenylpropanoid pathway—such as *HuCRRSP55* (HU07G01714), *HuPRP* (HU09G00039), and *HuPGLR*—were upregulated, particularly in the EX, contributing to the biosynthesis of lignin, flavonoids, and other antioxidants.

As senescence progressed, the TF *HuFULL* showed high expression during mid-senescence (Figure 5F). It was upregulated in cluster 1 of the EX and in all ME cell clusters except cluster 14 (Supplemental Data 11). During the later stages of senescence, genes specifically expressed in the ENF became active. These included *HuNCED1* (HU08G01783), a key gene involved in carotenoid biosynthesis, and numerous TFs, such as *HuERF30-1* (HU06G00852) and *HuERF28* (HU05G01921) from the ERF family (Figure 5F).

Our integrated single-cell transcriptomic and metabolomic analyses revealed that HuFULL and HuERF30-1 were key TFs

(C) Hierarchical clustering heatmap showing the abundance of the top 26 metabolites identified from the Venn diagram in Figure 4E.

(D) Co-expression network of transcription factors, key genes, and metabolites regulated by TAP4. Polanrazine B refers to polanrazine B_120170. NP-015301 refers to 9-(2,3-dihydroxypropoxy)-9-oxononanoic acid. Kaempferol refers to kaempferol-3-O-arabinofuranoside. Phosphatidylserine refers to phosphatidylserine 18:0–20:5.

(E) O2PLS loading plot of metabolites and genes involved in TAP4 regulation. Circles and triangles represent individual gene and metabolite loading values, respectively.

(F) Heatmap showing the expression of hub genes along the pseudotime trajectory.

(G–J) Changes in fruit phenotype at day 9 **(G)**, H_2O_2 localization detected by DAB staining **(H)**, flavonoid and superoxide levels in the exocarp and endocarp/fiber components **(I)**, and ROS localization detected using the H_2DCFDA probe **(J)** after silencing the hub genes. Experiments were performed with three biological replicates.

(K) RT–qPCR analysis of gene expression in RNA-silencing lines of *HuFULL*, *HuERF30-1*, and *HuCMB1*. Different VIGS samples were used to assess different genes: samples for *HuFULL*, *HuRHM1-1*, and *HuCYP98A2* were from the *HuFULL*-silenced line; samples for *HuCMB1* and *HuCRRSP55* were from the *HuCMB1*-silenced line; and samples for *HuERF30-1* and *HuNCED1* were from the *HuERF30-1*-silenced line.

Data are presented as mean ± standard deviation (SD). Each result was obtained from three biological replicates. Statistical significance was determined using *t* tests, and * indicates a significant difference between treatments ($p < 0.05$).

regulating downstream flavonoid and carotenoid biosynthesis, respectively. To validate the critical roles of *HuFULL* and other genes in resistance rebuilding, RNA-silencing lines were generated using VIGS for genes such as *HuFULL* and *HuERF30-1* (Supplemental Data 11). Compared with the control, fruit in the TAP4-treated group retained good quality; however, silencing of *HuPIP2-8*, *HuERF30-1*, and *HuFULL* significantly diminished the anti-senescence effects of TAP4. In particular, silencing of *HuPIP2-8* and *HuFULL* accelerated color darkening, desiccation, and lesion development in fruit (Figure 5G and Supplemental Figure S7A).

3,3′-Diaminobenzidine (DAB) staining for H_2O_2 showed that during the early stage of fruit senescence (3 days), H_2O_2 accumulation in the EX significantly increased in the TAP4-treated group compared with the control group (TAP vs. CK, $p < 0.05$) (Figure 5H). However, in the *PIP*-silenced group, no notable accumulation of H_2O_2 was observed in EX cells (PIPi vs. CK, $p > 0.05$). These findings suggest that TAP4 likely induced H_2O_2 influx, which was suppressed when the key aquaporin-encoding gene *HuPIP2-8* was silenced. During the mid-stage of senescence (6 days), extensive H_2O_2 deposition was observed throughout the pericarp in the control group, including the EX, ME, and ENF components. In contrast, the TAP4 group maintained extremely low H_2O_2 levels, with EX $H_{2O_2\ levels}$ even lower than those observed at day 3. In the *PIP*-silenced group, H_2O_2 accumulation resembled that of the control group, indicating a loss of TAP4-mediated anti-senescence effects (PIPi vs. CK, $p > 0.05$). These results suggest that a pronounced oxidative burst occurred during mid-senescence and that TAP4 effectively inhibited excessive H_2O_2 accumulation in fruit cells, thereby delaying senescence. At the late stage of senescence (9 days), H_2O_2 accumulation further intensified in the control group. H_2O_2 levels in the TAP4 group approached those of the control group, whereas in the *HuPIP2-8*-silenced group, H_2O_2 primarily accumulated in the ENF (Figure 5H and Supplemental Data 1). The *FULL*- and *ERF*-silenced groups exhibited similar phenotypes. On day 6, when pronounced H_2O_2 accumulation was observed in the CK group, H_2O_2 levels were even higher in *FULL*- and *ERF*-silenced fruit. By day 9, massive H_2O_2 accumulation was detected (Supplemental Figure 7B). These results indicate that silencing of FULL and ERF impairs the biosynthesis of antioxidant compounds, such as flavonoids, thereby leading to elevated H_2O_2 levels.

During the senescence process, TAP4 significantly reduced early flavonoid biosynthesis in the EX of *H*. *undatus* (day 1, $p < 0.01$) and delayed the second peak (Figure 5I). After silencing *HuPIP2-8*, the effect of TAP4 on inducing flavonoid biosynthesis in the EX was completely lost. Silencing *HuFULL* significantly reduced flavonoid biosynthesis in the EX from the mid-senescence stage compared with the TAP4 group (day 9, $p < 0.01$). However, silencing *HuERF30-1* did not significantly affect TAP4-induced flavonoid biosynthesis in the EX of *H*. *undatus*. In the ENF, there was almost no fluctuation in flavonoid biosynthesis in the CK group during fruit senescence. After TAP4 treatment, flavonoid biosynthesis began to increase on day 3, peaked on day 6, and significantly decreased on day 9. Silencing *HuPIP2-8* or the TFs *HuFULL* and *HuERF30-1* significantly impaired TAP4-induced flavonoid biosynthesis in the EN (days 6 and 9, $p < 0.01$) (Figure 5I).

TAP4 significantly reduced superoxide anion levels in both the EX and ENF of *H*. *undatus*, and this effect persisted into the later stages of senescence (day 12). Silencing *HuPIP2-8*, *HuFULL*, and *HuERF30-1* weakened TAP4-mediated scavenging of superoxide anions in the pericarp to varying degrees (Figure 5I).

Fluorescence staining using the ROS probe H_2DCFDA showed that TAP4 maintained ROS at very low levels during the mid-senescence stage (day 6) in the control group. By day 9, ROS levels in the pericarp of the TAP4 group increased significantly (Figure 5J). Following silencing of *HuFULL*, ROS levels in the EX began to increase from the mid-senescence stage and were significantly elevated in the late senescence stage compared with the TAP4 group. Silencing *HuERF30-1* not only increased ROS levels in the EX during both mid- and late senescence stages but also resulted in a more pronounced increase in ROS levels in the ENF (Supplemental Figure 7C).

The RT–qPCR results showed that in *HuFULL*-silenced lines, *HuFULL* expression was significantly downregulated, whereas the expression of flavonoid biosynthesis-related genes *HuRHM1-1* and *HuCYP98A2* was significantly reduced in the EN. In *HuERF30-1*-silenced lines, the expression of the target gene *HuERF30-1* in the EN was significantly downregulated, whereas that of *HuNCED1* was significantly decreased (Figure 5K). In *HuCMB1*-silenced lines, the expression of the target gene *HuCMB1* in the EX was significantly downregulated, while that of *HuCRRSP55* was significantly reduced (Figure 5K).

TAP4 activates immune responses in the inner pericarp of *H*. *undatus*

To elucidate the upstream regulatory mechanisms by which TAP4 activates immune responses and modulates downstream flavonoid and carotenoid biosynthesis, we screened immune-related genes and analyzed their expression patterns along pseudotime trajectories. The results showed that several immune receptors—including the pattern recognition receptor (PRR) gene *HuPBS1-2*, the RLK complex genes *HuCERK1-2* and *HuLYK6*, and the LRR-M/MLD-RLK complex genes *HuIGP4* and *HuLLG1*—as well as the cell wall extensin gene *HuLRX4* and the RALF-encoding gene *HuRALF6*, exhibited peak expression at the mid-senescence stage and were specifically upregulated in inner pericarp cells (Supplemental Figure 7D and Supplemental Data 7).

Additionally, a large number of genes associated with the mitogen-activated protein kinase (MAPK) signaling pathway were specifically upregulated in the inner pericarp during mid-senescence. Among them, *HuWRKY21-1* (HU05G00106) showed high expression from early to mid-senescence, with strong specificity in the inner pericarp (Supplemental Figure 7D and Supplemental Data 7).

Pseudotime trajectory analysis revealed that *HuPIP2-8* was highly expressed at the earliest stage, with expression detected across EX, ME, and ENF cells (Supplemental Figure 7E). The resistance-related gene *HuCRRSP55* was also activated early but showed specific expression in EX cells (Supplemental Figures 7D and 7E). Subsequently, immune-related genes, such as *HuWRKY21-1* and *HuLYK6*, were co-expressed during the

same phase, exhibiting clear inner pericarp specificity (Supplemental Figures 7D and 7E). *HuFULL* was upregulated at the mid-senescence stage, and its downstream flavonoid biosynthesis gene, *HuRHM1-1*, was also specifically expressed in the inner pericarp. In contrast, *HuERF30-1* and its downstream carotenoid biosynthesis gene, *HuNCED1*, were strongly expressed at later stages, again showing inner pericarp specificity (Supplemental Figures 7D and 7E).

Following TAP4 treatment, the lesion area was significantly reduced by 56.7% and 77.1% at day 6 (mid senescence) and day 9 (late senescence), respectively, compared with the CK group ($p < 0.01$). In *HuWRKY21-1*-silenced plants, the lesion area was 0.9-fold and 6.7-fold higher than that in the TAP4-treated group at days 6 and 9, respectively ($p < 0.01$) (Figure 6A).

The effect of TAP4 on the microbial community structure of *H. undatus* pericarp was also investigated. At the genus level, principal coordinate analysis (PCoA) revealed that TAP4 treatment induced a significant shift in the fungal community structure, whereas the bacterial community composition remained largely unchanged (Supplemental Figure 8A). Therefore, subsequent analyses focused on fungi. Beta diversity analysis indicated that, compared with the post samples, the abundance of Sordariomycetes significantly increased in TAP4-treated samples, whereas the abundance of *Cladosporium* was markedly reduced (Supplemental Figure 8B). Multilevel species difference analysis using linear discriminant analysis effect size (LEfSe) further identified Sordariomycetes as a key taxon enriched in TAP4-treated samples (Supplemental Figure 8C). Eight fungal strains were isolated and purified from *H. undatus* pericarp (Supplemental Figure 8D). In addition to molds (FFP-4, *Fusarium equiseti*), strain FFP-8 was identified as an intermediate form of *Candida* belonging to Sordariomycetes, further confirming that Sordariomycetes was a key fungal taxon enriched by TAP4 treatment (Supplemental Figure 8E). Confrontation assays on agar plates showed that TAP4 alone did not inhibit mold growth; however, it significantly enhanced the inhibitory effect of *Candida* on molds (Supplemental Figure 8F).

Fluorescence staining of callose revealed a significant increase in callose accumulation at day 3, indicative of an early immune response, with the strongest signal detected in the inner pericarp after TAP4 treatment ($p < 0.01$) (Figure 6B and Supplemental Data 1). In *HuWRKY21-1*-silenced fruit, TAP4-induced callose levels were comparable to those in the CK group ($p > 0.05$). In contrast, callose accumulation in the *HuFULL*- and *HuERF30-1*-silenced lines remained comparable to that in the TAP4-treated group ($p > 0.05$), indicating that silencing these TFs did not significantly affect early immune activation. Notably, in the *HuPIP2-8*-silenced line, although callose accumulation in the ME was comparable to that in the TAP4-treated group ($p > 0.05$), it was significantly reduced in the inner pericarp ($p < 0.01$) (Figure 6B and Supplemental Data 1).

TAP4 treatment significantly suppressed malondialdehyde (MDA) accumulation, whereas silencing *HuWRKY21-1*, *HuFULL*, *HuERF30-1*, or *HuPIP2-8* led to increased MDA levels compared with the TAP4 group (Figure 6C). Although TAP4 still induced flavonoid biosynthesis in the *HuWRKY21-1*-silenced line, the peak flavonoid level in the EX was comparable to that under TAP4 treatment, but the timing of the peak was delayed. In the inner pericarp, TAP4-induced flavonoid biosynthesis was maintained but exhibited greater fluctuations compared with the sustained elevation observed in the TAP4 group (Figures 6D and 6E).

RT–qPCR analysis confirmed that *HuWRKY21-1* expression was significantly upregulated in the inner pericarp following TAP4 treatment ($p < 0.01$) and was markedly suppressed in *HuWRKY21-1*-silenced fruit. Immune-related genes induced by TAP4, including *HuIGP4*, *HuLLG1*, *HuPBS1-2*, and *HuLYK6*, were all significantly downregulated in the silenced line ($p < 0.01$) (Figure 6F). Furthermore, the downstream TFs *HuFULL* and *HuERF30-1* were also strongly suppressed ($p < 0.01$), whereas their respective target genes, *HuRHM1-1* and *HuNCED1*, showed paradoxically higher expression levels ($p < 0.01$) (Figure 6G), suggesting potential compensatory regulation by other co-expressed TFs within the network. The *HuCERK1* promoter contains two W-box binding sites (Figure 6H). Dual-luciferase assays showed that, compared with the negative control, stronger fluorescence signals were observed in tissues co-infiltrated with *HuWRKY21*-REN and *ProHuCERK1*-LUC (Figures 6I and 6J). Consistently, the relative LUC/REN activity in *Nicotiana benthamiana* leaves co-expressing *HuWRKY21*-REN and *ProHuCERK1*-LUC was significantly increased (Figure 6K). These results indicate that *HuWRKY21* binds to the *HuCERK1* promoter.

In addition, TAP4 treatment markedly altered the structure of the fungal community on the fruit surface, enriching taxa such as Sordariomyces and enhancing their antagonistic activity against pathogenic molds. However, the current results do not allow us to distinguish whether the enhanced disease resistance of *H. undatus* was directly caused by TAP4-induced immune activation in the fruit or indirectly mediated by TAP4-driven changes in the microbial community. Whether biocontrol fungi such as Sordariomyces act synergistically with TAP4 to further enhance disease resistance in *H. undatus* remains to be elucidated.

Finally, a schematic model illustrating the molecular mechanism was constructed, showing that TAP4 activates EN-specific immune responses, in which the key TF *HuWRKY21-1* induces downstream genes *HuFULL* and *HuERFs* to regulate the flavonoid and carotenoid biosynthesis pathways, respectively (Figure 7).

DISCUSSION

The ability of Try to scavenge superoxide anions has been previously reported by our research group (Li et al., 2017; 2018), and its role in the postharvest preservation of fruit has also been confirmed in several studies (Li et al., 2021a; Pang et al., 2021; Wang et al., 2023a; 2023b). With the advancement of our research, we found that the superoxide anion scavenging activity of Try is independent of the protein's spatial structure. Given that Try undergoes pronounced autolysis (Vestling et al., 1990; Szenthe et al., 2005; Heissel et al., 2019), we hypothesized that its superoxide anion scavenging activity may originate from peptides generated during autolysis. Through peptide sequence identification, *in vitro* synthesis of seven key target peptide segments, and assessment of their superoxide anion scavenging activity, TAP4 was identified as

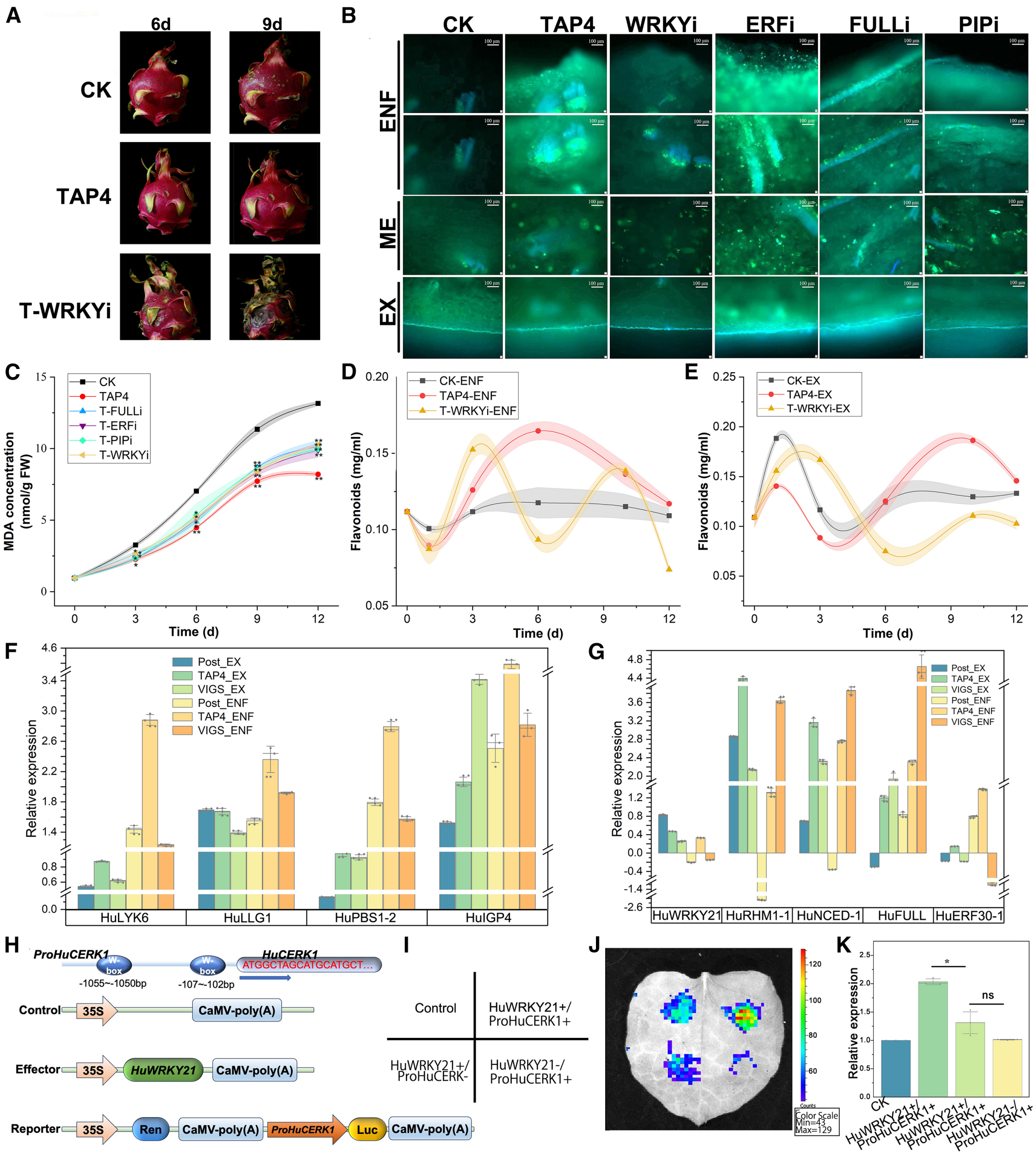

Figure 6. Functional validation of the key transcription factor *HuWRKY21-1*.
(A) Changes in fruit phenotype after silencing *HuWRKY21-1*.
(B) Differences in callose accumulation among different silenced lines. EX, exocarp; ME, mesocarp; ENF, endocarp fiber.
(C–E) Effects of silencing *HuWRKY21-1* on MDA levels **(C)**, flavonoid levels in the endocarp **(D)**, and flavonoid levels in the exocarp **(E)**.
(F and G) RT–qPCR analysis of the effects of silencing *HuWRKY21-1* on the expression of downstream genes **(F)** and immune-related genes **(G)**.
(H) Schematic diagram of vector construction for dual-luciferase assays.
(I) Diagram of infiltration sites in tobacco leaves.
(J) Fluorescence images of co-infiltrated sites.
(K) Relative LUC/REN activity in *Nicotiana benthamiana* leaves co-expressing *HuWRKY21* and *ProHuCERK1*.
Data are presented as mean ± standard deviation (SD). Each result was obtained from three biological replicates. Statistical significance was determined using *t* tests, and * indicates a significant difference between treatments ($p < 0.05$).

the most active peptide among the autolysis-derived peptides of Try (Figure 1).

After identifying the most active peptide segment among the Try autolysis-derived peptides, an interesting question arose: what is the mechanism by which it delays senescence in *H. undatus* fruit? During the normal postharvest senescence process of *H. undatus*, the EX is responsible for the formation of resistance during the early stages of senescence (Li et al., 2024a; Jia et al., 2025), whereas the ME is involved in metabolic processes at later stages (Li et al., 2024a; Pang et al., 2024). How, then, does TAP4 delay fruit senescence? To address this question,

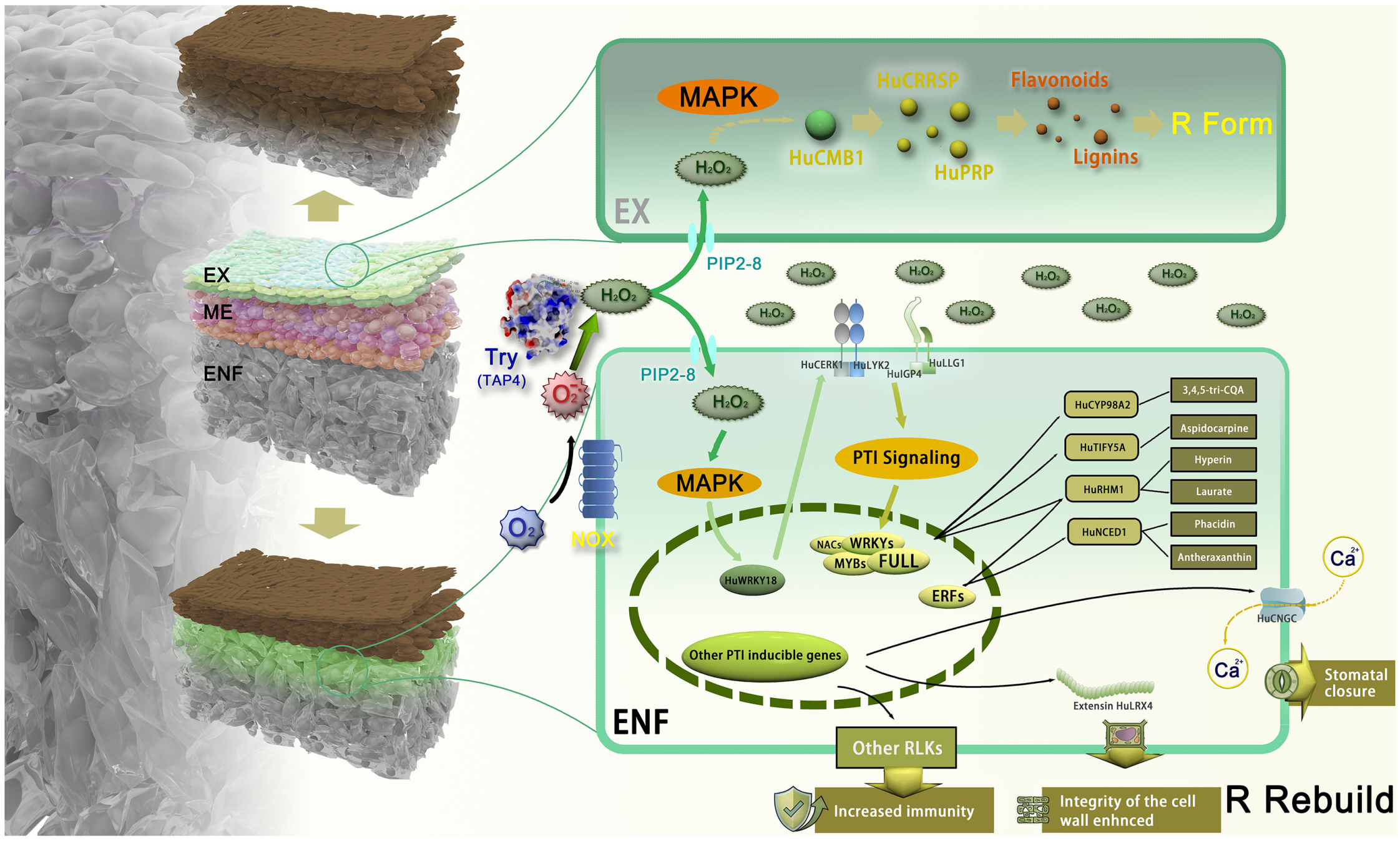

Figure 7. Hypothesized trajectory of TAP4-induced immune activation in endocarp cells during fruit senescence.
Our results showed that fruit senescence exhibited significant spatiotemporal transitions. H_2O_2, transported into cells via the aquaporin PIP2-8, served as the initial signal for senescence. In the early stages of senescence, resistance genes were significantly upregulated in exocarp cells. The key transcription factor *HuCMB1*, which responded early, activated resistance genes such as *HuCRRSP55*, thereby promoting flavonoid and lignin synthesis and forming the first stage of resistance. In the middle to late stages of fruit senescence, transcription factors such as *HuERF30-1* were highly expressed in the endocarp, where they activated genes such as *HuNCED1* and regulated the biosynthesis of antioxidants, including carotenoids. This resulted in the formation of the second stage of resistance, indicating that fruit resistance was rebuilt. EX represents the exocarp, ME the mesocarp, and ENF the endocarp fiber. NOX denotes NADPH oxidase. Try denotes trypsin, and TAP denotes the trypsin autolysis peptide. PIP2-8 refers to the aquaporin HuPIP2-8. "R form" and "R rebuild" represent resistance formation and rebuilding, respectively.

we applied single-cell transcriptomic analysis to investigate the heterogeneity of TAP4-induced transcriptional changes across different cell clusters, with the aim of elucidating the underlying mechanisms.

H. undatus (pitaya) has been reclassified as *Selenicereus undatus* (Li et al., 2024a; 2024d). However, because gene names for pitaya in databases begin with "Hu," this study retains the use of *H. undatus* for consistency and reader clarity. Upon TAP4 treatment, 26 cell clusters were identified by single-cell analysis (Figure 2B). However, due to the lack of comprehensive marker gene databases for pitaya, a primary challenge is assigning specific cell types to these clusters.

To spatially resolve the 26 cell clusters identified by scRNA-seq, we employed stRNA-seq in combination with four integration algorithms (Pang et al., 2024; Jia et al., 2025). This analysis allowed us to map the cellular composition within each cluster (Figures 2E and 2F).

Metabolite MS detection and sequential window acquisition of all theoretical fragment-ion spectra (SWATH) analyses revealed that TAP4 treatment significantly induced 26 metabolites, among which two flavonoids, a chlorogenic acid derivative, and a carotenoid were identified as core components (Figures 6A–6C). The metabolic profile, including alkaloids, terpenoids, fatty acids, and phenylpropanoids (but not lignin-related compounds), suggests that resistance in the EN is primarily reconstituted through flavonoids and carotenoids to maintain redox homeostasis. In contrast, resistance formation in the EX requires lignin-related compounds to reinforce the cell wall. Flavonoids extracted from plants have been extensively reported to exert anti-senescence effects in animal cells (Fan et al., 2022; Delenko et al., 2025). It has also been reported that promoting flavonoid accumulation can delay fruit senescence (Wang et al., 2024b). Anthocyanins protect photosynthetic tissues from excessive light and facilitate nutrient remobilization in leaves during senescence (Hoch et al., 2003). Moreover, the exogenous application of flavonoids has been widely used in the preservation of fruits and vegetables, as well as livestock, aquatic, and meat products (Wang et al., 2016; Zhang et al., 2023). Collectively, these studies indicate that flavonoid biosynthesis plays an important role in fruit anti-senescence processes.

Fruit senescence is generally regulated by TFs, which finely modulate the aging process by binding to the promoter regions of downstream target genes (Giovannoni et al., 2017; Zhang et al., 2019a; Liu et al., 2022). Among these, the Apetala 2 (AP2)/ERF family plays a key role downstream of the ethylene signaling pathway (Liu et al., 2014; Xie et al., 2016). However, in this study, *H. undatus*, a non-climacteric fruit, produces extremely low levels of ethylene during senescence (Li et al., 2024a); therefore, ERF expression may be regulated through ethylene-independent pathways.

Co-expression network analysis revealed that multiple members of the ERF family play key roles during senescence. Among them, *HuERF30-1* was induced at the mid-senescence stage and was specifically upregulated in EN cells. Silencing *HuERF30-1* led to the downregulation of *HuNCED1*, a key gene involved in carotenoid biosynthesis, and was accompanied by a significant reduction in flavonoid levels. In normal senescence samples (post), *HuNCED1* was not expressed; however, upon TAP4 treatment, both *HuERF30-1* and *HuNCED1* were activated in the EN. Although *HuNCED1* expression was markedly reduced after *HuERF30-1* silencing, residual expression remained, suggesting that other ERFs, such as HuERF28, may also participate in this regulatory process (Figure 6K). These findings indicate that the carotenoid biosynthetic pathway was inactive in the EN during normal senescence but was activated during TAP4-induced resistance reconstruction. Notably, this study demonstrates for the first time that, in a non-climacteric fruit, ERFs such as *HuERF30-1* positively regulate carotenoid accumulation. This regulatory pattern contrasts with the negative roles of ERFs in climacteric fruits such as tomato (Dong et al., 2013), but resembles the positive regulation observed in apple (Dang et al., 2021), highlighting the diversity of regulatory mechanisms underlying fruit senescence.

Another key TF, *HuFULL*, was broadly expressed during mid-senescence without tissue specificity. Silencing *HuFULL* significantly affected flavonoid biosynthesis and superoxide levels (Figure 6I). RT–qPCR analysis showed that flavonoid-related genes *HuRHM1-1* and *HuCYP98A2* were significantly downregulated in the pericarp following *HuFULL* silencing (Figure 6K), indicating that *HuFULL* contributs to resistance reconstruction in the EN by regulating these genes. However, the full functional role of *HuFULL* remains unclear and requires further investigation.

This study primarily explored the TF–gene–metabolite regulatory pathways involved in TAP4-induced fruit resistance. However, how the upstream immune response is activated remains an intriguing question. Single-cell transcriptomic analysis revealed that several PTI-related genes were specifically upregulated in the EN. These included the PRR-encoding gene *HuPBS1-2*, RLK complex genes (*HuCERK1-2* and *HuLYK6*), and LMR complex genes (*HuIGP4* and *HuLLG1*), whose expression peaks coincided with the period of resistance reconstruction (Figures 5 and 6). In addition, the cell wall extension-related gene *HuLRX4* (Neubauer et al., 2012; Hu et al., 2014) and the RALF gene *HuRALF6* were also specifically expressed in the EN (Supplemental Data 7), suggesting that they may participate in signal perception and immune activation (Escobar-Restrepo et al., 2007; Ge et al., 2017; Duan et al., 2020; Ortiz-Morea et al., 2022).

Based on the temporal patterns of gene expression, *HuPIP2-8*, which encodes an H_2O_2 transporter, responded first, followed by the activation of the EX-specific resistance gene *HuCRRSP55*, the immune-related transcription factor *HuWRKY21-1*, and the immune receptor gene *HuLYK6*. In downstream metabolic pathways, flavonoid biosynthesis, regulated by *HuFULL*, was activated earlier than the carotenoid biosynthesis pathway regulated by *HuERF30-1* (Supplemental Figures 7D and 7E). Callose deposition assays confirmed that strong signals appeared in the EN 3 days after TAP4 treatment, and this process depended on *HuWRKY21-1*. Silencing the downstream TF *HuFULL* or *HuERF30-1* did not affect callose accumulation, indicating that they act downstream of the immune response. In contrast, silencing *HuPIP2-8* blocked the transmission of the H_2O_2 signal to the EN and suppressed callose deposition, suggesting that H_2O_2 is a key signal for immune activation.

Flavonoid accumulation dynamics further supported this model. Although TAP4 still induced flavonoid biosynthesis in *HuWRKY21-1*-silenced fruit, the prolonged and stable high-level synthesis observed in the control group was lost and replaced by strong fluctuations. This indicates that *HuWRKY21-1* plays a central role in maintaining sustained induction of key metabolites such as flavonoids. However, the partial maintenance of flavonoid biosynthesis in the absence of *HuWRKY21-1* suggests the involvement of compensatory TFs.

RT–qPCR analysis further confirmed the pivotal role of *HuWRKY21-1* in TAP4-mediated activation of upstream immune responses (Figure 6). Immune receptor genes, including *HuIGP4*, *HuLLG1*, *HuPBS1-2*, and *HuLYK6*, were significantly downregulated in *HuWRKY21-1*-silenced fruit, indicating that they are under the control of *HuWRKY21-1*. Similarly, the downstream TFs *HuFULL* and *HuERF30-1* were also repressed upon *HuWRKY21-1* silencing. Interestingly, the expression of their respective target genes, *HuRHM1-1* and *HuNCED1*, which are involved in flavonoid and carotenoid biosynthesis, was significantly upregulated, suggesting that the synthesis of these key metabolites is also regulated by additional co-expressed TFs. This finding provides further evidence for the observed fluctuations in flavonoid biosynthesis in *HuWRKY21-1*-silenced lines.

Taken together, we propose the following model: during postharvest resistance reconstruction in *H*. *undatus* fruit, H_2O_2 activates the MAPK pathway and induces the expression of *WRKY* TFs, particularly *HuWRKY21-1*. This activation subsequently triggers expression of the CERK/LYK and LMR–LLG complexes, thereby initiating immune responses. RALF6 and LRX4 are likely involved in upstream signal perception. Resistance formation occurs in two distinct phases: in the early stage, *HuCMB1* regulates genes such as *HuCRRSP55* in the EX to promote phenylpropanoid synthesis; in the mid stage, *HuWRKY21-1*, *HuFULL*, and *HuERF30-1* induce the accumulation of flavonoids and carotenoids in the EN. TAP4 specifically activates immune responses in the EN to mediate resistance reconstruction, representing a mechanism distinct from the natural resistance formation observed in the EX.

The immune cells identified in the EN in this study exhibited tissue-specific differences from the primed cells reported in leaves (Nobori et al., 2025), and the modes of immune activation also differed between these organs. These results suggest that specialized immune cell populations may exist across multiple plant organs, including leaves and fruits, warranting further investigation. It has long been believed that delaying fruit ripening can enhance fruit resistance to pathogens; however, this approach can compromise the full development of fruit nutritional and flavor quality, making it difficult to achieve both quality and resistance. In this study, resistance formation and nutrient metabolism during fruit

senescence were likely mediated by distinct cell clusters. This finding provides a potential new breeding strategy: enhancing resistance by activating immune responses in the EN while maintaining quality-related metabolic processes in the EX. Collectively, these findings offer new insights for reducing post-harvest losses and extending storage life.

METHODS

Plant growth conditions

The present study utilized *H. undatus* fruits (Vietnam No. 1 cultivar) collected at 35 days after artificial pollination from the Miaoshui experimental base in Ruyang County, Luoyang City, Henan Province, China. The harvested fruits were used as mature samples for analysis and were maintained under controlled environmental conditions at 25 °C and 85% relative humidity.

Isolation, identification, and synthesis of TAPs

Try (bovine, 5×10^5 units/g) was purchased from Amresco (USA). A Try solution (2.41×10^{-4} mol/L) was prepared using double-distilled water ($ddH_{2O)}$. Nano-LC–MS/MS was employed to identify bovine TAPs (Lee et al., 2024). A 25 μL peptide mixture (1 mg) was separated using a Nano-LC system equipped with a Reprosil-Pur 120 C18 nano-capture column (100 μm × 180 mm, 3 μm particle size) (EASY-nLC 1200 System, Thermo Fisher Scientific, USA) and analyzed on an Orbitrap Exploris 240 mass spectrometer (Thermo Fisher Scientific, USA). Full MS scans were performed within the 100–1500 *m/z* range to generate raw mass spectrometry data. Peptide sequences were then analyzed using PEAKS Studio software with the following search parameters: database, Trypsin_DNA_bovine.fasta; MS1 tolerance, 20 ppm; and MS2 tolerance (fragment mass tolerance), 0.02 Da.

Peptides were synthesized using solid-phase synthesis methods (Noki et al., 2024). Modified resin was used to initiate peptide synthesis. A reaction mixture of piperidine/N,N-dimethylformamind (pip/DMF) was added to the reactor, which was then shaken for a certain period. The solvent was filtered out, and DMF was added to the system. The reactor was shaken for 1 min, and the liquid was removed; this step was repeated three times. The prepared amino acid solution was then added to the reactor along with 1 ml of differential interference contrast (DIC)/DMF solution, and the reactor was shaken for a specified duration. The resin was then washed, and the procedure was repeated by adding pip/DMF. After another wash, the corresponding amino acids were added until peptide synthesis was completed.

Try and TAP4 treatment of *H. undatus* fruit

Thirty randomly selected *H. undatus* fruits were immersed in Try or TAP4 solutions (2.41×10^{-6} mol/L) for 40 s. The control group consisted of 15 fruits treated with PBS buffer under the same conditions. All treated fruits were immediately transferred to a growth chamber (25 °C, 85% relative humidity) and stored for 12 days. Phenotypic parameters (e.g., weight loss rate, flavonoid levels, and superoxide anion levels) were measured periodically. Lesion area was quantified using ImageJ (https://imagej.net) at each time point.

Detection of callose deposition

Pericarp tissue slices (~2 mm thick) were fixed in acetic acid-ethanol-formaldehyde (AAF) solution for 24 h, washed twice with absolute ethanol (1 min each), and then transferred to 10 volumes of 100% ethanol for storage. Prior to staining, tissues were equilibrated in 50% ethanol for 30 min and briefly air-dried. Sections were incubated in aniline blue staining solution at room temperature in the dark for 1 h. After adding 25 μl of antifade mounting medium, samples were observed under a fluorescence microscope (Leica DM2500) as previously described (Zhang et al., 2018b). Callose fluorescence intensity was quantified using ImageJ.

Pathogen isolation, purification, and identification

Fungal pathogens were isolated from decaying fruit based on visible symptoms and hyphal morphology using tissue isolation and standard microbial inoculation methods. Colonies were grown on luria-bertani (LB) or potato dextrose agar (PDA) solid media, and single colonies were purified through repeated streaking (Masyahit et al., 2009; Valencia-Botín et al., 2013). For fungal identification, genomic DNA was extracted, and internal transcribed spacer (ITS) regions were amplified using universal fungal primers ITS1F (5′-CTTGGTCATTTAGAGGAAGTAA-3′) and ITS2R (5′-GCTGCGTTCTTCATCGATGC-3′). For bacterial pathogens, 16S rRNA genes were amplified using primers 799F (5′-AACMGGATTAGATACCCKG-3′) and 1193R (5′-ACGTCATCCCCACCTTCC-3′). PCR products were subjected to Sanger sequencing and analyzed using BLASTN (https://blast.ncbi.nlm.nih.gov/Blast.cgi) against the NCBI nucleotide database (Faja et al., 2019). Closely related sequences with high homology and morphological similarity were selected and aligned. Phylogenetic trees were constructed using the neighbor-joining (NJ) method in MEGA7.0 with 1000 bootstrap replicates to determine pathogen taxonomy.

Fruit disease resistance assay

Pathogenic molds isolated from decayed *H. undatus* tissues were cultured on PDA plates at 25 °C until hyphae developed. Mycelial plugs (3 mm diameter) were transferred to fresh PDA plates and incubated for 2 days to obtain actively growing mycelia. Detached *H. undatus* fruits were inoculated with a single plug beneath a bract, while control fruits were inoculated with sterile PDA plugs (He et al., 2023). Fruits were incubated at 28 °C for 6 days. Lesion areas were photographed and analyzed using ImageJ software (https://imagej.net) (Wu et al., 2024). Each group included at least 5 fruits for resistance evaluation.

Microbial diversity analysis

Total genomic DNA of microbial communities was extracted using the E.Z.N.A. Soil DNA Kit (Omega Bio-tek, Norcross, GA, USA). DNA quality and concentration were assessed prior to downstream analysis. The V3–V4 region of the 16S rRNA gene was amplified using barcode-tagged primers 338F (5′-ACTCCTACGGGAGGCAGCAG-3′) and 806R (5′-GGACTACHVGGGTWTCTAAT-3′) (Liu et al., 2016). PCR products were purified, and sequencing libraries were constructed using the NEXTFLEX Rapid DNA-Seq Kit. Sequencing was performed on the Illumina NextSeq 2000 platform (Shanghai BajorBio Biomedical Technology Co., Ltd.).

Sequencing data were processed using QIIME2 with the DADA2 plugin for denoising (Edgar et al., 2013). Operational taxonomic unit (OTU) classification was performed using a Naive Bayes classifier against the Silva 16S rRNA database (v138). Taxonomic assignment and community diversity analyses were carried out using standard QIIME2 pipelines (Barberan et al., 2012).

Protoplast isolation, scRNA-seq library preparation, and sequencing

H. undatus (Vietnam No. 1) fruits, harvested 35 days after artificial pollination (DAAP), were collected from the Miaoshui base in Ruyang County, Luoyang City, Henan Province, China. Harvested fruits were used as mature samples (the CK group). Fruits stored at room temperature were used as senescence samples (the post group), showing visible wrinkles or dehydration-induced lesions on the pericarp. Fruits treated with Try or TAP4 (2.41×10^{-6} mol/L) were used as the Try or TAP4 group. All groups were stored simultaneously. At the end of the storage period (12 days), freshly harvested mature fruits were collected and used as the CK group. All three sample groups underwent processing and high-throughput sequencing within the same batch. For each group, three fruits were collected, and three pericarp regions (approximately 1 cm^2) were sampled from each fruit. The tissues were placed in RNase-free enzyme digestion buffer and digested at room temperature for 0.5 h. Protoplasts from the pericarp of the CK, post, and TAP4 groups were isolated

according to the method described by Carqueijeiro et al. (2018), with slight modifications.

H. undatus cell suspensions were loaded onto the 10× Genomics Chromium system to generate single-cell gel beads in emulsion (GEMs). Following the protocol provided with the 10× Genomics reagent kit, scRNA-seq libraries of the pericarp were prepared. The libraries were quantified using a high-sensitivity DNA chip on the 2100 Bioanalyzer (Agilent) and a Qubit High Sensitivity DNA Assay Kit (Thermo Fisher Scientific). Sequencing was performed on the NovaSeq 6000 system (Illumina), generating 150-bp paired-end reads.

scRNA-seq data analysis

Sequencing data were processed using the Cell Ranger pipeline (v7.1.0) with default and recommended settings. The STAR algorithm was used to align FASTQ files to the *H. undatus* genome (Le et al., 2017). After calculating unique molecular identifiers (UMIs) and removing non-cell-associated barcodes, a gene–barcode matrix comprising cell barcodes and gene expression counts was generated. This output was imported into the Seurat R package (v4.1.1) for quality control and downstream analysis of scRNA-seq data (Satija et al., 2015). The FindIntegrationAnchors and IntegrateData functions were used to identify integration "anchors" across datasets. Normalized data for a subset of highly variable genes were obtained using the NormalizeData function in Seurat. The data were scaled, and principal component analysis (PCA) was performed, retaining the top 30 components. A two-dimensional map was generated using UMAP, and clustering results were visualized (Satija et al., 2015).

Dimensionality reduction and clustering of cell subclusters were conducted using the Louvain algorithm implemented in the FindClusters function in Seurat. Cell type annotation was conducted using semi-supervised category identification and assignment (SCINA) and known marker genes (Zhang et al., 2019b). To identify genes with significant differential expression across clusters, the Wilcoxon rank-sum test was used for pairwise comparisons between clusters (Zhang et al., 2021).

Single-cell trajectories were constructed and visualized in UMAP-reduced dimensional space using the built-in Monocle3 algorithms learn_graph and order_cells (Cao et al., 2019; Guan et al., 2025). Genes with significant branch-dependent expression were identified using graph_test, and the dynamic expression patterns of the top 100 PRGs were visualized using plot_genes_in_pseudotime to complete the pseudotemporal analysis (Trapnell et al., 2014; Qiu et al., 2017).

For RNA velocity analysis, the scVelo software (Si et al., 2023) was used to calculate the ratios of unspliced to spliced mRNA within cells to estimate changes in RNA abundance over time and infer the potential differentiation direction of cells. The resulting RNA velocity vectors were projected onto the pseudotemporal trajectory generated by Monocle3, enabling integration of the two approaches and assessment of their consistency.

stRNA-seq sample preparation, library construction, sequencing, and data analysis

The pericarp of mature *H. undatus* fruit was embedded in cold OCT (Sakura, 4583) and placed onto a 10× Genomics spatial gene expression slide. Frozen tissue sections (10 μm thick) were prepared using a cryostat (Leica, CM1950). Sections were fixed in pre-chilled methanol and processed according to the Visium Spatial Gene Expression User Guide (catalog no. CG000239 Rev A, 10× Genomics). Fluorescence images were captured using a TRITC filter under a 20× objective lens, and a permeabilization time of 3 min was determined to be optimal.

Spatial transcriptomics (ST) libraries were prepared following the Visium Spatial Gene Expression User Guide (CG000239; https://assets.ctfassets.net/an68im79xiti/3pyXucRaiKWcscXy3cmRHL/a1ba41c77cbf60366202805ead8f64d7/CG000239_VisiumSpatialGeneExpression_UserGuide_Rev_A.pdf). The libraries were loaded at a concentration of 300 pM and sequenced on the NovaSeq X Plus platform using the NovaSeq S4 reagent kit (200 cycles, catalog no. 20027466, Illumina). The sequencing protocol consisted of read 1 (28 cycles), i7 index read (10 cycles), i5 index read (10 cycles), and read 2 (91 cycles).

For tissue sections attached to the capture areas, fixation, histological staining, and tissue clearing were performed. After transcripts were captured by surface-bound probes, reverse transcription was carried out overnight. Following on-array cDNA synthesis, tissues were removed, and the spatial barcode–mRNA–cDNA hybrids from each capture area were released from the array, collected into tubes, and used for library preparation. The ST libraries were subsequently generated and sequenced on an Illumina platform.

Data processing, alignment, and UMI count summarization for each spot on the Visium spatial transcriptomics array were performed using Space Ranger software (v1.2.2; 10× Genomics). For unsupervised graph-based clustering of spatial features, the Louvain method was applied (Lafzi et al., 2018; McInnes et al., 2018). DEGs between distinct samples or clusters were identified using the FindMarkers function in Seurat, with likelihood ratio tests used for statistical analysis.

To identify clusters corresponding to those defined in the scRNA-seq data, four algorithms—SingleR (Stuart et al., 2019), SciBet (Li et al., 2020), RCTD (Cable et al., 2022), and CARD (Ma and Zhou, 2022)—were used to perform correlation analysis between the stRNA-seq and scRNA-seq datasets. These complementary algorithms were employed for cell type annotation to increase robustness. SingleR relies on correlation with reference datasets, SciBet uses a machine-learning classifier for low-expression cells, RCTD integrates spatial and single-cell transcriptomes, and CARD applies a Bayesian model to handle high heterogeneity. Each cell was independently annotated using SingleR (Stuart et al., 2019), implemented via the open-source R package available on GitHub (Supplemental Data 12). Training–test splitting, cross-validation, and supervised cell type annotation were performed using SciBet (Li et al., 2020), with all functions implemented in the corresponding R package (Supplemental Data 12). In heatmaps generated by SingleR and SciBet, clusters were grouped based on correlations between single-cell and spatial transcriptomic clusters. For the RCTD method, we followed the guidelines provided in the RCTD GitHub repository (Supplemental Data 12), with the doublet_mode parameter set to "full" (Cable et al., 2022). For CARD, in scRNA-seq data, B represents a $G \times K$ cell type-specific expression matrix of informative genes, where each element denotes the average expression level of informative genes in a specific cell type (Ma and Zhou, 2022). This matrix B is commonly referred to as the reference basis matrix. In spatial transcriptomics data, X represents a $G \times N$ gene expression matrix of the same set of informative genes across N spatial locations, and V denotes an $N \times K$ cell type composition matrix, where each row indicates the proportions of K cell types at each spatial location. The objective was to estimate V by integrating spatial transcriptomics data (X) with the scRNA-seq-derived reference matrix (B). To achieve this, a non-negative matrix factorization model was constructed to link these three matrices.

Metabolite detection by UPLC–MS/MS and metabolomics analysis

Metabolites in the EX, ME, and EN of *H. undatus* were analyzed at day 0 and at early (day 3), middle (day 6), and late (day 9) stages of senescence using ultra performance liquid chromatography-tandem mass spectrometry (UPLC–MS/MS) with SWATH-based data acquisition (Li et al., 2024a). At each time point, tissue layers were separated under a dissecting microscope (Olympus SZX7). Lyophilized samples were ground in liquid nitrogen, and metabolites were extracted with 70% aqueous methanol at 60°C for 3 h. The extracts were centrifuged and filtered through a 0.22 μm membrane prior to analysis. Liquid chromatography separation

was performed using a Shimadzu Nexera UPLC system (Shimadzu, Kyoto, Japan) equipped with a binary 30A pump, a SIL-30AC autosampler, and a CTO-30AC column oven. Separation was achieved on a Phenomenex Kinetix C18 column (2.1 × 100 mm, 100 Å, 1.7 μm) fitted with a C18 guard column (2.0 mm I.D. × 4.0 mm; Phenomenex Luna). The column oven was set to 50°C, and the autosampler was maintained at 8°C. Mobile phase A (MPA) consisted of 1% ACN with 0.1% formic acid (v/v), and mobile phase B (MPB) consisted of 90% ACN with 1% formic acid (v/v).

The gradient elution program was as follows: an initial isocratic step at 10% MPB for 1.0 min with the column flow directed to waste, after which the flow was redirected to the mass spectrometer. A linear gradient from 10% to 50% MPB was applied from 1 to 8 min, followed by a linear increase from 50% to 99% MPB from 8 to 10.5 min. The composition was then held at 90% MPB for 2.5 min. Finally, the column was returned to the initial conditions and equilibrated for 15 min, completing the gradient program.

Mass spectrometry analysis was conducted using an AB Sciex X500B mass spectrometer with a Turbo V ion source operating in both positive and negative ionization modes. The source conditions were set as follows: ion spray voltage, 5.5 kV in positive mode (4.5 kV in negative mode); declustering potential, 40 V; and collision energy, 5 V. The turbo spray temperature was maintained at 500°C, with nebulizer gas (Gas 1) at 50 psi, heater gas (Gas 2) at 50 psi, and curtain gas at 30 psi. Continuous recalibration was performed every hour by injecting and analyzing the appropriate X500B calibration mixture using the automatic calibration delivery system. All parameters were controlled and run using Sciex OS v3.0 software (Sciex, Concord, Ontario, Canada). SWATH acquisition mode was applied, and data were processed using MS-DIAL software.

Metabolomics data analysis was performed on a cloud-based platform, including the identification of differential metabolites and O2PLS analysis. PCA and PLS–DA were used to visualize metabolic alterations across experimental groups. The union set of differentially up- or downregulated metabolites from all time points was clustered using Short Time-series Expression Miner (STEM) v1.3.11, with a significance threshold of $p < 0.05$ (Xu et al., 2020).

Bidirectional O2PLS was employed to integrate metabolomics and transcriptomics data (Liu et al., 2021). Loading values (X_loadings for the transcriptome and Y_loadings for the metabolome) were calculated to evaluate the contribution of each variable to intergroup differences and were used for plotting.

Gene–metabolite networks were constructed using the SCODE algorithm, and the resulting networks were visualized in Cytoscape. Core genes and key metabolites were identified using the CytoHubba plugin.

VIGS of hub genes

Fragments of *HuCMB1* (178 bp), *HuPIP2-8* (226 bp), *HuFULL* (243 bp), *HuWRKY21-1* (360 bp), and *HuERF30-1* (198 bp) were amplified from *H. undatus* cDNA and subsequently cloned into the pTRV2 vector. The pTRV1, pTRV2, pTRV2-HuCMB1, pTRV2-HuPIP2-8, pTRV2-HuFULL, pTRV2-HuWRKY21-1, and pTRV2-HuERF30-1 constructs were then introduced into *Agrobacterium tumefaciens* strain GV3101 (Supplemental Data 13). The infection procedure was carried out as described by Zhang et al. (2018a). The pericarps of *H. undatus* were submerged in a bacterial suspension with an optical density at 600 nm (OD_{600}) of 0.6 and incubated at 25°C in the dark for 48 h. Five biological replicates were performed for each experiment.

Dual-luciferase assay

HuWRKY21 was cloned into the pGreenII 62-SK vector, and the *HuCERK1* promoter was cloned into the pGreenII 0800-LUC vector. The two constructs were separately transformed into *A. tumefaciens* strain GV3101 (Weidibio Co., Ltd., Shanghai, China), and the resulting cultures were co-infiltrated into *N. benthamiana* leaves. Luciferase (LUC) and renilla luciferase (REN) activities were measured using the TransDetect Double-Luciferase Reporter Assay Kit (TransGen Biotech Co., Ltd., Beijing, China). Fluorescence images were acquired using an IVIS Lumina III imaging system (Revvity, Shanghai, China). Each experiment was performed with three biological replicates. Primer sequences are listed in Supplemental Data 13.

Gene expression analysis by RT–qPCR

RT–qPCR was conducted following the protocol outlined by Li et al. (2024a, 2024b, 2024c, 2024d). The *β-actin* gene (HU11G01228) from *H. undatus* was used as the internal control. Relative gene expression levels were calculated using the $2^{-\Delta\Delta Ct}$ method. The primers used for RT–qPCR are listed in Supplemental Data 13. Three biological replicates were performed for each analysis.

ROS and flavonoid determination

ROS levels in the pericarp were determined using a previously described method (Li et al., 2024a). Fluorescence of ROS stained with H_2DCFDA was measured using a fluorescence microscope (Leica DM2500, Germany) with an excitation wavelength of 488 nm and an emission range of 515–540 nm.

Superoxide anion content was measured using the hydroxylamine oxidation method (Satija et al., 2015), with appropriate modifications. A 10 mM hydroxylamine hydrochloride solution was added to the homogenate supernatant of *H. undatus* EX and EN tissues, and the mixture was shaken for 30 min. Then, 17 mM p-aminobenzenesulfonic acid and 7 mM α-naphthylamine were added. After standing for 15 min, the absorbance was measured at 530 nm. For assessment of superoxide anion–scavenging activity of Try or TAPs, superoxide anions were generated by DMSO under alkaline conditions, and changes in superoxide anion levels following treatment with Try or TAPs were determined using the hydroxylamine oxidation method (Mittler et al., 2022). In this experiment, ddH_2O was used as the control, and the concentrations of both the Try and TAP solutions were 2.41×10^{-4} mol/l.

DAB staining for H_2O_2 detection was performed following the protocol described by Zhang et al. (2018b), with minor modifications. Pericarp tissues of *H. undatus* were sliced into ~2-mm-thick sections and incubated in DAB solution (1 mg/ml, pH 3.8) in the dark for 6 h. Staining was considered complete when reddish-brown coloration appeared in positive regions, while the remaining tissue was nearly colorless or retained its natural pigmentation.

The tissues were then decolorized in 95% ethanol at 80°C in a water bath, with the ethanol replaced every 10 min. After decolorization, samples were stored in 20% glycerol. Reddish-brown deposits were considered indicative of H_2O_2 accumulation and were imaged under a light microscope (Nikon Eclipse Ci, Japan). The relative intensity of the staining was quantified using ImageJ (https://imagej.net).

Total flavonoid content was determined using the aluminum chloride colorimetric method (Ghasemzadeh et al., 2016). The ethanol extract of flavonoids was mixed with $NaNO_2$ solution. After incubation at 25°C for 6 min, $Al(NO_3)_3$ solution was added, mixed thoroughly, and allowed to stand for 6 min. Then, NaOH solution was added, and the reaction mixture was incubated at room temperature for 10 min. Absorbance was measured at 510 nm. Rutin (purity > 98%; CAS Registry Number 153-18-4) was used as the standard to generate a calibration curve.

RNA fluorescence *in situ* hybridization (RNA-FISH)

After dehydration, tissue blocks were embedded in paraffin and sectioned into 5-μm-thick slices using a microtome (Leica RM2016, Germany).

Sections were deparaffinized twice in xylene and subsequently rehydrated through graded ethanol solutions (100%, 95%, 80%, and 70%). Samples were then fixed in 4% RNase-free paraformaldehyde (PFA). A humidified chamber was prepared using 5× SSC (pH 7.5) and formamide (1:1, v/v). Samples were treated with 30% H_2O_2 in pure methanol (1:9) for 10 min, followed by incubation with 0.25% hydrochloric acid at room temperature for 15 min. Proteinase K was applied to cover the samples, which were then incubated at 37°C in a molecular hybridization oven for 20 min. The reaction was terminated by washing with 0.1 M glycine solution for 1 min. After each step, samples were rinsed 2–3 times with diethyl pyrocarbonate (DEPC)-treated water for 1 min each. Finally, tissues were fixed again in 4% PFA for 10 min.

Probes were applied to cover the sections, followed by hybridization at 65°C for 48 h. Sections were then stained with 1 μg/ml 4′,6-diamidino-2-phenylindole (DAPI) at room temperature for 5 min. Images were acquired using a fluorescence upright microscope (Leica DM2500, Germany), and fluorescence intensity was analyzed using ImageJ. Fluorescent oligonucleotide probes for HU08G02237, Hu02G03369, and HU06G00852 were synthesized by GeFan Biotechnology Co., Ltd. (China) (Pang et al., 2024). Probe sequences are provided in Supplemental Data 13.

Statistical analysis

Statistical analyses were performed using OriginPro 2021 (version 9.8.0.200). The number of multiple comparisons was determined based on the total number of genes in the R data serizlization (RDS) dataset (n = 20,357). The p-values were calculated using a one-sided hypergeometric model. Quantification of fluorescence images for callose deposition and DAB-stained areas was performed using ImageJ (https://imagej.net).

Paired-sample t tests were used to evaluate differences in physiological and biochemical indicators, such as flavonoid content and superoxide levels, between experimental groups. Paired-sample t tests were also applied to assess differences in H_2O_2 accumulation, as indicated by DAB staining, between the TAP-treated group and the control group (TAP vs. CK), as well as between the *PIP2-8*-silenced group and the control group (PIPi vs. CK).

DATA AND CODE AVAILABILITY

The scRNA-seq and stRNA-seq data generated in this study have been deposited in the NCBI sequence read archive (SRA) database under BioProject numbers "PRJNA974579" (https://www.ncbi.nlm.nih.gov/sra/PRJNA974579) and "PRJNA1002459" (https://www.ncbi.nlm.nih.gov/sra/PRJNA1002459). These datasets are also available in the ScienceDB database (https://www.scidb.cn/en) under CSTR numbers "31 253.11.sciencedb.29907" (https://doi.org/10.57760/sciencedb.29907) and "31 253.11.sciencedb.30032" (https://doi.org/10.57760/sciencedb.30032). This study does not report original code. Additional information required to reanalyze the data reported in this work is available from the lead contact upon request.

FUNDING

This work was supported by the National Natural Science Foundation of China (No. U23A20264); the Funding Program of Wuhu Green Food Industry Research Institute Co., Ltd. (No. LSSPCYYJY-2025KF-01); the National and Local Joint Engineering Laboratory of High- Efficiency, Superior-Quality Cultivation and Fruit Deep Processing Technology of Characteristic Fruit Trees in South Xinjiang, China (No. FE202303); and the Key Research and Development Program of Henan Province (No. 261111521700).

ACKNOWLEDGMENTS

We thank the Majorbio I-Sanger Cloud Platform (www.i-sanger.com) for providing a free online platform. We also thank Suqian Qinyan Information Technology Co., Ltd. for assistance with drawing the model diagram. No conflict of interest is declared.

AUTHOR CONTRIBUTIONS

Overall conceptualization and design of the research design: X.L. and R.J.H.; methodology: X.S. and Y.W.; investigation: Y.T., M.L., and P.M.; formal analysis: P.M., R.G., and Q.W.; software: R.G. and Q.W.; data curation: Y.T. and M.L.; visualization: X.L., X.D., G.Z., and X.P.; writing—original draft: X.L., X.D., G.Z., and X.P. All authors commented on the manuscript.

SUPPLEMENTAL INFORMATION

Supplemental information is available at *Plant Communications Online*.

Received: November 21, 2025
Revised: January 28, 2026
Accepted: April 3, 2026
Published: April 7, 2026

REFERENCES

Apostolopoulos, V., Bojarska, J., Chai, T.T., Elnagdy, S., Kaczmarek, K., Matsoukas, J., New, R., Parang, K., Lopez, O.P., Parhiz, H., et al. (2021). A global review on short peptides: Frontiers and perspectives. Molecules **26**:430. https://doi.org/10.3390/molecules26020430.

Barberán, A., Bates, S.T., Casamayor, E.O., and Fierer, N. (2012). Using network analysis to explore co-occurrence patterns in soil microbial communities. ISME J. **6**:343–351. https://doi.org/10.1038/ismej.2011.119.

Cable, D.M., Murray, E., Zou, L.S., Goeva, A., Macosko, E.Z., Chen, F., and Irizarry, R.A. (2022). Robust decomposition of cell type mixtures in spatial transcriptomics. Nat. Biotechnol. **40**:517–526. https://doi.org/10.1038/s41587-021-00830-w.

Cao, J., Spielmann, M., Qiu, X., Huang, X., Ibrahim, D.M., Hill, A.J., Zhang, F., Mundlos, S., Christiansen, L., Steemers, F.J., et al. (2019). The single-cell transcriptional landscape of mammalian organogenesis. Nature **566**:496–502. https://doi.org/10.21037/tcr-2025-377.

Carqueijeiro, I., Noronha, H., Bettencourt, S., Guedes, J.G., Duarte, P., Gerós, H., and Sottomayor, M. (2018). Isolation of vacuoles from the leaves of the medicinal plant *Catharanthus roseus*. Methods Mol. Biol. **1789**:81–99. https://doi.org/10.1007/978-1-4939-7856-4.

Chekan, J.R., Mydy, L.S., Pasquale, M.A., and Kersten, R.D. (2024). Plant peptides - redefining an area of ribosomally synthesized and post-translationally modified peptides. Nat. Prod. Rep. **41**:1020–1059. https://doi.org/10.1039/d3np00042g.

Couto, D., and Zipfel, C. (2016). Regulation of pattern recognition receptor signalling in plants. Nat. Rev. Immunol. **16**:537–552. https://doi.org/10.1038/nri.2016.77.

Dang, Q., Sha, H., Nie, J., Wang, Y., Yuan, Y., and Jia, D. (2021). An apple (*Malus domestica*) AP2/ERF transcription factor modulates carotenoid accumulation. Hortic. Res. **8**:223. https://doi.org/10.1038/s41438-021-00694-w.

Delenko, J., Hyman, N., Chatterjee, P.K., Safaric Tepes, P., Shih, A.J., Xue, X., Gurney, J., Baker, A.G., Wei, C., Munoz Espin, D., et al. (2025). Targeting cellular senescence to enhance human endometrial stromal cell decidualization and inhibit their migration. Biomolecules **15**:873. https://doi.org/10.3390/biom15060873.

Dong, T., Hu, Z., Deng, L., Wang, Y., Zhu, M., Zhang, J., and Chen, G. (2013). A tomato MADS-box transcription factor, SlMADS1, acts as a negative regulator of fruit ripening. Plant Physiol. **163**:1026–1036. https://doi.org/10.1104/pp.113.224436.

Duan, Q., Liu, M.C.J., Kita, D., Jordan, S.S., Yeh, F.L.J., Yvon, R., Carpenter, H., Federico, A.N., Garcia-Valencia, L.E., Eyles, S.J., et al. (2020). FERONIA controls pectin- and nitric oxide-mediated

male-female interaction. Nature **579**:561–566. https://doi.org/10.1038/s41586-020-2106-2.

Edgar, R.C. (2013). UPARSE: highly accurate ASV sequences from microbial amplicon reads. Nat. Methods **10**:996–998. https://doi.org/10.1038/nmeth.2604.

Escobar-Restrepo, J.M., Huck, N., Kessler, S., Gagliardini, V., Gheyselinck, J., Yang, W.C., and Grossniklaus, U. (2007). The FERONIA receptor-like kinase mediates male-female interactions during pollen tube reception. Science **317**:656–660. https://doi.org/10.1126/science.1143562.

Faja, O.M., Sharad, A.A., Younis, K.M., Alwan, M.G., Mohammed, B.J., and Ahmad, A. (2019). Isolation, detection of virulence genes, antibiotic resistance genes, plasmid profile, and molecular typing among Vibrio parahaemolyticus isolated in Malaysian seawater from recreational beaches and fish. Vet. World **12**:1140–1149. https://doi.org/10.14202/vetworld.2019.1140-1149.

Fan, X., Fan, Z., Yang, Z., Huang, T., Tong, Y., Yang, D., Mao, X., and Yang, M. (2022). Flavonoids-natural gifts to promote health and longevity. Int. J. Mol. Sci. **23**:2176. https://doi.org/10.3390/ijms23042176.

Ge, Z., Bergonci, T., Zhao, Y., Zou, Y., Du, S., Liu, M.C., Luo, X., Ruan, H., García-Valencia, L.E., Zhong, S., et al. (2017). Arabidopsis pollen tube integrity and sperm release are regulated by RALF-mediated signaling. Science **358**:1596–1600. https://doi.org/10.1126/science.aao3642.

Ghasemzadeh, A., Jaafar, H.Z.E., and Rahmat, A. (2016). Variation of the phytochemical constituents and antioxidant activities of Zingiber officinale var. rubrum Theilade associated with different drying methods and polyphenol oxidase activity. Molecules **21**:780. https://doi.org/10.3390/molecules21060780.

Giovannoni, J., Nguyen, C., Ampofo, B., Zhong, S., and Fei, Z. (2017). The epigenome and transcriptional dynamics of fruit ripening. Annu. Rev. Genet. **68**:61–84. https://doi.org/10.1146/annurev-arplant-042916-040906.

Guan, Y., Luan, Y., Zhao, S., Li, M., Girolamo, F., Palmer, J.D., and Guan, Q. (2025). Single-cell RNA sequencing for characterizing the immune communication and iron metabolism roles in CD31+ glioma cells. Transl. Cancer Res. **14**:2421–2439. https://doi.org/10.21037/tcr-2025-377.

Guo, J., and Cheng, Y. (2022). Advances in fungal elicitor-triggered plant immunity. Int. J. Mol. Sci. **23**:12003. https://doi.org/10.3390/ijms231912003.

He, Z., Peng, S., Yin, Q., Huang, Y., Deng, T., Luo, Y., and He, N. (2024). Ss4368: Pathogen-associated molecular pattern for inducing plant cell death and resistance to *Phytophthora capsici*. Int. J. Mol. Sci. **25**:8674. https://doi.org/10.3390/ijms25168674.

Heissel, S., Frederiksen, S.J., Bunkenborg, J., and Højrup, P. (2019). Enhanced trypsin on a budget: Stabilization, purification and high-temperature application of inexpensive commercial trypsin for proteomics applications. PLoS One **14**:e0218374. https://doi.org/10.1371/journal.pone.0218374.

He, Y.H., Chen, S.Y., Chen, X.Y., Xu, Y.P., Liang, Y., and Cai, X.Z. (2023). RALF22 promotes plant immunity and amplifies the Pep3 immune signal. J. Integr. Plant Biol. **65**:2519–2534. https://doi.org/10.1111/jipb.13566.

Hoch, W.A., Singsaas, E.L., and McCown, B.H. (2003). Resorption protection. Anthocyanins facilitate nutrient recovery in autumn by shielding leaves from potentially damaging light levels. Plant Physiol. **133**:1296–1305. https://doi.org/10.1104/pp.103.027631.

Huang, K., Xu, Y., Feng, T., Lan, H., Ling, F., Xiang, H., and Liu, Q. (2024). The advancement and application of the single-cell transcriptome in biological and medical research. Biology **13**:451. https://doi.org/10.3390/biology13060451.

Höhn, A., Weber, D., Jung, T., Ott, C., Hugo, M., Kochlik, B., Kehm, R., König, J., Grune, T., and Castro, J.P. (2017). Happily (n)ever after: Aging in the context of oxidative stress, proteostasis loss and cellular senescence. Redox Biol. **11**:482–501. https://doi.org/10.1016/j.redox.2016.12.001.

Hu, B., Liu, B., Liu, L., Liu, C., Xu, L., and Ruan, Y. (2014). Epigenetic control of pollen ole e 1 allergen and extensin family gene expression in *Arabidopsis thaliana*. Acta Physiol. Plant. **36**:2203–2209. https://doi.org/10.1007/s11738-014-1597-6.

James, M., Poret, M., Masclaux-Daubresse, C., Marmagne, A., Coquet, L., Jouenne, T., Chan, P., Trouverie, J., and Etienne, P. (2018). SAG12, a major cysteine protease involved in nitrogen allocation during senescence for seed production in *Arabidopsis thaliana*. Plant Cell Physiol. **59**:2052–2063. https://doi.org/10.1093/pcp/pcy125.

Jia, J., Chen, E., Tian, Y., Shen, J., Pang, X., and Li, X. (2025). Fine localization and functional differentiation of exocarp cell clusters during fruit senescence revealed by single-cell and spatial transcriptomics. Postharvest Biol. Tec. **219**:113223. https://doi.org/10.1016/j.postharvbio.2024.113223.

Jennings, C., West, J., Waine, C., Craik, D., and Anderson, M. (2001). Biosynthesis and insecticidal properties of plant cyclotides: the cyclic knotted proteins from Oldenlandia affinis. Proc. Natl. Acad. Sci. USA **98**:10614–10619. https://doi.org/10.1073/pnas.191366898.

Jones, J.D.G., and Dangl, J.L. (2006). The plant immune system. Nature **444**:323–329. https://doi.org/10.1038/nature05286.

Khavinson, V.K., Popovich, I.G., Linkova, N.S., Mironova, E.S., and Ilina, A.R. (2021). Peptide regulation of gene expression: A systematic review. Molecules **26**:7053. https://doi.org/10.3390/molecules26227053.

Klarzynski, O., Plesse, B., Joubert, J.M., Yvin, J.C., Kopp, M., Kloareg, B., and Fritig, B. (2000). Linear beta-1,3 glucans are elicitors of defense responses in tobacco. Plant Physiol. **124**:1027–1038. https://doi.org/10.1104/pp.124.3.1027.

Lafzi, A., Moutinho, C., Picelli, S., and Heyn, H. (2018). Tutorial: guidelines for the experimental design of single-cell RNA sequencing studies. Nat. Protoc. **13**:2742–2757. https://doi.org/10.1038/s41596-018-0073-y.

Le, D.T., Durham, J.N., Smith, K.N., Wang, H., Bartlett, B.R., Aulakh, L.K., Lu, S., Kemberling, H., Wilt, C., Luber, B.S., et al. (2017). Mismatch repair deficiency predicts response of solid tumors to PD-1 blockade. Science **357**:409–413. https://doi.org/10.1126/science.aan6733.

Lee, C.H., Hamdan, N., Nyakuma, B.B., Wong, S.L., Wong, K.Y., Tan, H., Jamaluddin, H., and Lee, T.H. (2024). Purification identification and molecular docking studies of antioxidant and anti-inflammatory peptides from Edible Bird's Nest. Food Chem. **454**:139797. https://doi.org/10.1016/j.foodchem.2024.139797.

Li, C., Liu, B., Kang, B., Liu, Z., Liu, Y., Chen, C., Ren, X., and Zhang, Z. (2020). SciBet as a portable and fast single cell type identifier. Nat. Commun. **11**:1818. https://doi.org/10.1038/s41467-020-15523-2.

Li, H., Xu, W.X., Tan, J.C., Hong, Y.M., He, J., Zhao, B.P., Zhou, J.A., Zheng, Y.M., Lei, M., Zheng, X.Q., et al. (2024b). Single-cell multi-omics identify novel regulators required for osteoclastogenesis during aging. iScience **27**:110734. https://doi.org/10.1016/j.isci.2024.110734.

Li, P., Ma, X., Li, Z., Yao, H., Lu, G., Hu, H., and Ding, Y. (2024d). A review on the advances of dragon fruit. Trop. Plants **3**:0. https://doi.org/10.48130/tp-0024-0041.

Li, X., Li, B., Guan, S., Cai, L., and Xinyue, P. (2021a). Hub genes and sub-networks of stoma-related genes in *Hylocereus undatus* through trypsin treatment during storage revealed by transcriptomic analysis. Food Biochem **45**:e13538. https://doi.org/10.1111/jfbc.13538.

Li, X., Li, B., Gu, S., Pang, X., Mason, P., Yuan, J., Jia, J., Sun, J., Zhao, C., and Henry, R. (2024a). Single-cell and spatial RNA sequencing reveal the spatiotemporal trajectories of fruit senescence. Nat. Commun. **15**:3108. https://doi.org/10.1038/s41467-024-47329-x.

Li, X., Tang, Z., Pang, X., Zhao, C., Li, X., and Liu, Y. (2017). Trypsin slows the ageing of mice due to its novel superoxide scavenging activity. Appl. Biochem. Biotechnol. **181**:1549–1560. https://doi.org/10.1007/s12010-016-2301-7.

Li, X., Zhang, Y., Zhao, S., Li, B., Cai, L., and Pang, X. (2021b). Omics analyses indicate the routes of lignin related metabolites regulated by trypsin during storage of pitaya (*Hylocereus undatus*). Genomics **113**:3681–3695. https://doi.org/10.1016/j.ygeno.2021.08.005.

Li, X., Zhong, Y., and Zhao, C. (2018). Trypsin binding with copper ions scavenges superoxide: Molecular dynamics-based mechanism investigation. Int. J. Environ. Res. Pub. He. **15**:139. https://doi.org/10.3390/ijerph15010139.

Li, Y., Tian, X., Luo, J., Bao, T., Wang, S., and Wu, X. (2024c). Molecular mechanisms of aging and anti-aging strategies. Cell Commun. Signal. **22**:285. https://doi.org/10.1186/s12964-024-01663-1.

Liu, B., Li, C., Li, Z., Wang, D., Ren, X., and Zhang, Z. (2020). An entropy-based metric for assessing the purity of single cell populations. Nat. Commun. **11**:3155. https://doi.org/10.1038/s41467-020-16904-3.

Liu, C., Zhao, D., Ma, W., Guo, Y., Wang, A., Wang, Q., and Lee, D.J. (2016). Denitrifying sulfide removal process on high-salinity wastewaters in the presence of *Halomonas* sp. Appl. Microbiol. Biot. **100**:1421–1426. https://doi.org/10.1007/s00253-015-7039-6.

Liu, G.S., Li, H.L., Grierson, D., and Fu, D.Q. (2022). NAC transcription factor family regulation of fruit ripening and quality: A review. Cells **11**:525. https://doi.org/10.3390/cells11030525.

Liu, J., Gao, Y., Gong, F., Hou, F., Zhang, Z., Cheng, X., Du, W., Zhang, L., Wang, J., Xu, J., et al. (2021). The transcriptome and metabolome reveal stress responses in sulfur-fumigated cucumber (*Cucumis sativus* L.). Front. Plant Sci. **12**:778956. https://doi.org/10.3389/fpls.2021.778956.

Liu, M., Diretto, G., Pirrello, J., Roustan, J.P., Li, Z., Giuliano, G., Regad, F., and Bouzayen, M. (2014). The chimeric repressor version of an Ethylene Response Factor (ERF) family member, Sl-ERF.B3, shows contrasting effects on tomato fruit ripening. New Phytol. **203**:206–218. https://doi.org/10.1111/nph.12771.

Liu, T., Liu, Z., Song, C., Hu, Y., Han, Z., She, J., Fan, F., Wang, J., Jin, C., Chang, J., et al. (2012). Chitin-induced dimerization activates a plant immune receptor. Science **336**:1160–1164. https://doi.org/10.1126/science.1218867.

Maroux, S., and Desnuelle, P. (1969). On some autolyzed derivatives of bovine trypsin. Biochim. Biophys. Acta **181**:59–72. https://doi.org/10.1016/0005-2795(69)90227-x.

Masyahit, M., Sijam, K., Awang, Y., and Ghazali, M. (2009). First report on bacterial soft rot disease on pitaya (*Hylocereus* spp.) caused by Enterobacter cloacae in peninsular Malaysia. Int. J. Agric. Biol. **11**:659–666. http://www.fspublishers.org.

Mathiazhagan, M., Chidambara, B., Hunashikatti, L.R., and Ravishankar, K.V. (2021). Genomic approaches for improvement of tropical fruit: Fruit quality, shelf life and nutrient content. Genes **12**:1881. https://doi.org/10.3390/genes12121881.

Ma, Y., and Zhou, X. (2022). Spatially informed cell-type deconvolution for spatial transcriptomics. Nat. Biotechnol. **40**:1349–1359. https://doi.org/10.1038/s41587-022-01273-7.

Mcinnes, L., Healy, J., Saul, N., and Großberger, L. (2018). UMAP: Uniform manifold approximation and projection for dimension reduction. J. Open Source Softw. **3**:861. https://doi.org/10.21105/JOSS.00861.

Mir Drikvand, R., Sohrabi, S.M., Sohrabi, S.S., and Samiei, K. (2024). Molecular identification and characterization of hevein antimicrobial peptide genes in two-row and six-row cultivars of Barley (*Hordeum vulgare* L.). Biochem. Genet. **62**:5092–5114. https://doi.org/10.1007/s10528-024-10695-8.

Mittler, R., Zandalinas, S.I., Fichman, Y., and Van Breusegem, F. (2022). Reactive oxygen species signalling in plant stress responses. Nat. Rev. Mol. Cell Biol. **23**:663–679. https://doi.org/10.1038/s41580-022-00499-2.

Myat, A.A., Zhou, Y., Gao, Y., Zhao, X., Liang, C., Abid, M.A., Wang, P., Akram, U., Abbas, M., Askari, M., et al. (2022). Overexpression of GhKTI12 enhances seed yield and biomass production in *Nicotiana Tabacum*. Genes **13**:426. https://doi.org/10.3390/genes13030426.

Nambeesan, S., Datsenka, T., Ferruzzi, M.G., Malladi, A., Mattoo, A.K., and Handa, A.K. (2010). Overexpression of yeast spermidine synthase impacts ripening, senescence and decay symptoms in tomato. Plant J. **63**:836–847. https://doi.org/10.1111/j.1365-313X.2010.04286.x.

Neubauer, J.D., Lulai, E.C., Thompson, A.L., Suttle, J.C., and Bolton, M.D. (2012). Wounding coordinately induces cell wall protein, cell cycle and pectin methyl esterase genes involved in tuber closing layer and wound periderm development. J. Plant Physiol. **169**:586–595. https://doi.org/10.1016/j.jplph.2011.12.010.

Ngou, B.P.M., Ahn, H.K., Ding, P., and Jones, J.D.G. (2021). Mutual potentiation of plant immunity by cell-surface and intracellular receptors. Nature **592**:110–115. https://doi.org/10.1038/s41586-021-03315-7.

Nobori, T., Monell, A., Lee, T.A., Sakata, Y., Shirahama, S., Zhou, J., Nery, J.R., Mine, A., and Ecker, J.R. (2025). A rare PRIMER cell state in plant immunity. Nature **638**:197–205. https://doi.org/10.1038/s41586-024-08383-z.

Noki, S., de la Torre, B.G., and Albericio, F. (2024). Safety-catch linkers for solid-phase peptide synthesis. Molecules **29**:1429. https://doi.org/10.3390/molecules29071429.

Ortiz-Morea, F.A., Liu, J., Shan, L., and He, P. (2022). Malectin-like receptor kinases as protector deities in plant immunity. Nat. Plants **8**:27–37. https://doi.org/10.1038/s41477-021-01028-3.

Pang, X., Sun, J., Jia, J., Trusov, Y., Chandora, R., Botella, J.R., Li, X., and Gu, S. (2024). Integration of single-cell and spatial RNA sequencing uncovers spatiotemporal transition of fruit senescence trajectory from exocarp to mesocarp in pitaya (*Hylocereus undatus*). Postharvest Biol. Tec. **213**:112954. https://doi.org/10.1016/j.postharvbio.2024.112954.

Pang, X., Zhao, S., Zhang, M., Cai, L., Zhang, Y., and Li, X. (2021). Catechin gallate acts as a key metabolite induced by trypsin in *Hylocereus undatus* during storage indicated by omics. Plant Physiol. Bioch **158**:497–507. https://doi.org/10.1016/j.plaphy.2020.11.036.

Qiu, X., Mao, Q., Tang, Y., Wang, L., Chawla, R., Pliner, H.A., and Trapnell, C. (2017). Reversed graph embedding resolves complex single-cell trajectories. Nat. Methods **14**:979–982. https://doi.org/10.1038/nmeth.4402.

Satija, R., Farrell, J.A., Gennert, D., Schier, A.F., and Regev, A. (2015). Spatial reconstruction of single-cell gene expression data. Nat. Biotechnol. **33**:495–502. https://doi.org/10.1038/nbt.3192.

Szenthe, B., Frost, C., Szilágyi, L., Patthy, A., Naudé, R., and Gráf, L. (2005). Cloning and expression of ostrich trypsinogen: an avian trypsin with a highly sensitive autolysis site. Biochim. Biophys. Acta **1748**:35–42. https://doi.org/10.1016/j.bbapap.2004.12.006.

Si, W., Fang, C., Liu, C., Yin, M., Xu, W., Li, Y., Yan, X., Shen, Y., Cao, J., and Sun, J. (2023). Why is Babesia not killed by artemisinin like Plasmodium? Parasite. Vectors **16**:193. https://doi.org/10.1186/s13071-023-05783-4.

Stuart, T., Butler, A., Hoffman, P., Hafemeister, C., Papalexi, E., Mauck, W.M., Hao, Y., Stoeckius, M., Smibert, P., and Satija, R. (2019). Comprehensive integration of single-cell data. Cell **177**:1888–1902.e21. https://doi.org/10.1016/j.cell.2019.05.031.

Terekhova, M., Swain, A., Bohacova, P., Aladyeva, E., Arthur, L., Laha, A., Mogilenko, D.A., Burdess, S., Sukhov, V., Kleverov, D., et al. (2024). Single-cell atlas of healthy human blood unveils age-related loss of NKG2C+GZMB-CD8+memory T cells and accumulation of type 2 memory T cells. Immunity **57**:188–192. https://doi.org/10.1016/j.immuni.2023.12.014.

Trapnell, C., Cacchiarelli, D., Grimsby, J., Pokharel, P., Li, S., Morse, M., Lennon, N.J., Livak, K.J., Mikkelsen, T.S., and Rinn, J.L. (2014). The dynamics and regulators of cell fate decisions are revealed by pseudotemporal ordering of single cells. Nat. Biotechnol. **32**:381–386. https://doi.org/10.1038/nbt.2859.

Valencia-Botín, A.J., Kokubu, H., and Ortíz-Hernández, Y.D. (2013). A brief overview on pitahaya (*Hylocereus* spp.) diseases. Australas. Plant Pathol. **42**:437–440. https://doi.org/10.1007/s13313-012-0193-8.

Van de Sande, B., Lee, J.S., Mutasa-Gottgens, E., Naughton, B., Bacon, W., Manning, J., Wang, Y., Pollard, J., Mendez, M., Hill, J., et al. (2023). Applications of single-cell RNA sequencing in drug discovery and development. Nat. Rev. Drug Discov. **22**:496–520. https://doi.org/10.1038/s41573-023-00688-4.

Vestling, M.M., Murphy, C.M., and Fenselau, C. (1990). Recognition of trypsin autolysis products by high-performance liquid chromatography and mass spectrometry. Anal. Chem. **62**:2391–2394. https://doi.org/10.1021/ac00220a025.

Wang, H., Li, Y., Wassie, M., Huo, L., and Shi, H. (2024a). Salicylic acid spray delays sand pear fruit senescence during room temperature shelf life by regulating antioxidant capacity and senescence-related genes. Plants **13**:848. https://doi.org/10.3390/plants13060848.

Wang, J., Jia, J., Sun, J., Pang, X., Li, B., Yuan, J., Chen, E., and Li, X. (2023b). Trypsin preservation: CsUGT91C1 regulates trilobatin biosynthesis in *Cucumis sativus* during storage. Plant Growth Regul. **100**:633–646. https://doi.org/10.1007/s10725-023-00962-w.

Wang, J., Tian, P., Sun, J., Li, B., Jia, J., Yuan, J., Li, X., Gu, S., and Pang, X. (2023a). CsMYC2 is involved in the regulation of phenylpropanoid biosynthesis induced by trypsin in cucumber (*Cucumis sativus*) during storage. Plant Physiol. Biochem. **196**:65–74. https://doi.org/10.1016/j.plaphy.2023.01.041.

Wang, Y., Muzammal Aslam, M., Wang, X., Gu, H., Jia, W., Li, W., and Shao, Y. (2024b). Aloe vera and tea polyphenols composite coating delays passion fruit senescence by promoting phenolic and flavonoid accumulation. Food Res. Int. **190**:114594. https://doi.org/10.1016/j.foodres.2024.114594.

Wang, Y., Shan, T., Yuan, Y., and Yue, T. (2016). Overall quality properties of Kiwifruit treated by cinnamaldehyde and citral: Microbial, antioxidant capacity during cold storage. J. Food Sci. **81**:H3043–H3051. https://doi.org/10.1111/1750-3841.13536.

Wu, L.J., Han, C., Wang, H.M., He, Y.C., Lin, H., Wang, L., Chen, C., and Zhiguo, E. (2024). OsbZIP53 negatively regulates immunity response by involving in reactive oxygen species and salicylic acid metabolism in rice. Rice Sci. **31**:190–202. https://doi.org/10.1016/j.rsci.2023.12.002.

Xie, X.L., Yin, X.R., and Chen, K.S. (2016). Roles of APETALA2/ethylene-response factors in regulation of fruit quality. Crit. Rev. Plant Sci. **35**:120–130. https://doi.org/10.1080/07352689.2016.1213119.

Xu, J., Sun, M., Wang, Y., Xie, A., and Gao, J. (2020). Identification of hub genes of mesio temporal lobe epilepsy and prognostic biomarkers of brain low-grade gliomas based on bioinformatics analysis. Cell Transplant. **29**:963689720978722. https://doi.org/10.1177/0963689720978722.

Zhang, J., Wu, Z., Hu, F., Liu, L., Huang, X., Zhao, J., and Wang, H. (2018a). Aberrant seed development in *Litchi chinensis* is associated with the impaired expression of cell wall invertase genes. Hortic. Res. **5**:39. https://doi.org/10.1038/s41438-018-0042-1.

Zhang, X.R., Xu, Y.P., and Cai, X.Z. (2018b). SlCNGC1 and SlCNGC14 suppress *Xanthomonas oryzae* pv. *oryzicola*-induced hypersensitive response and non-host resistance in tomato. Front. Plant Sci. **9**:285. https://doi.org/10.3389/fpls.2018.00285.

Zhang, L., Chen, L., and Dong, H. (2019a). Plant aquaporins in infection by and immunity against pathogens - A critical review. Front. Plant Sci. **10**:632. https://doi.org/10.3389/fpls.2019.00632.

Zhang, T.Z., Li, J.H., Shen, A., and Li, S.Z. (2023). Effect of polymethoxy flavones from *Citrus* peels on antibacterial and fresh-keeping of red grape. Science and Technology of Food Industry **44**:143–150. https://doi.org/10.13386/j.issn1002-0306.2022090038.

Zhang, Z., Luo, D., Zhong, X., Choi, J.H., Ma, Y., Wang, S., Mahrt, E., Guo, W., Stawiski, E.W., Modrusan, Z., et al. (2019b). SCINA: A semi-supervised subtyping algorithm of single cells and bulksamples. Genes **10**:531. https://doi.org/10.3390/genes10070531.

Zhang, T.Q., Chen, Y., and Wang, J.W. (2021). A single-cell analysis of the *Arabidopsis* vegetative shoot apex. Dev. Cell **56**:1056–1074.e8. https://doi.org/10.1016/j.devcel.2021.02.021.

Zhang, Y., Butelli, E., De Stefano, R., Schoonbeek, H.J., Magusin, A., Pagliarani, C., Wellner, N., Hill, L., Orzaez, D., Granell, A., et al. (2013). Anthocyanins double the shelf life of tomatoes by delaying overripening and reducing susceptibility to gray mold. Curr. Biol. **23**:1094–1100. https://doi.org/10.1016/j.cub.2013.04.072.

Zheng, J., Meinhardt, L.W., Goenaga, R., Zhang, D., and Yin, Y. (2021). The chromosome-level genome of dragon fruit reveals whole-genome duplication and chromosomal co-localization of betacyanin biosynthetic genes. Hortic. Res. **8**:63. https://doi.org/10.1038/s41438-021-00501-6.

Zhu, F., Cao, M.Y., Zhang, Q.P., Mohan, R., Schar, J., Mitchell, M., Chen, H., Liu, F., Wang, D., and Fu, Z.Q. (2024). Join the green team: Inducers of plant immunity in the plant disease sustainable control toolbox. J. Adv. Res. **57**:15–42. https://doi.org/10.1016/j.jare.2023.04.016.

Plant Communications, Volume

Supplemental information

Single-cell and spatial transcriptomics reveals the spatiotemporal trajectory of the small peptide TAP4 in delaying postharvest fruit senescence

Xin Li, Yajing Tian, Xu Duan, Mingyue Lv, Xingfeng Shao, Yingying Wei, Patrick Mason, Ruining Guan, Qiyue Wang, Xinyue Pang, Guoqiang Zhang, and Robert J. Henry

Title Page

Single-cell and Spatial Transcriptomics Reveals the Spatiotemporal Trajectory of the Small Peptide TAP4 in Delaying Postharvest Fruit Senescence

Xin Li[1,2,3], Yajing Tian[1], Xu Duan[1], Mingyue Lv[1], Xingfeng Shao[4], Yingying Wei[4], Patrick Mason[2], Ruining Guan[5], Qiyue Wang[5], Xinyue Pang[2,6,*], Guoqiang Zhang[7,8,*], Robert J Henry[2,9*]

[1] College of Food and Bioengineering, Henan University of Science and Technology, Luoyang 471023, China.

[2] Queensland Alliance for Agriculture & Food Innovation, Queensland Biosciences Precinct, The University of Queensland, St Lucia QLD 4072, Australia.

[3] The National and Local Joint Engineering Laboratory of High Efficiency and Superior-Quality Cultivation and Fruit Deep Processing Technology of Characteristic Fruit Trees in South Xinjiang, Tarim University, Alar, 843300, China.

[4] Zhejiang-Malaysia Joint Research Laboratory for Agricultural Product Processing and Nutrition, College of Food Science and Engineering, Ningbo University, Ningbo 315800, China.

[5] School of Pharmacy, Lanzhou University, Lanzhou 730000, China.

[6] College of Medical Technology and Engineering, Henan University of Science and Technology, Luoyang 471023, China.

[7] Wuhu Green Food Industrial Research Institute Co., Ltd, Wuhu 241000, China.

[8] College of Biological and Food Engineering, Anhui Polytechnic University, Wuhu 241000, China.

[9] VinUni Big Data Research Institute, VinUniversity, Hanoi, Vietnam

*Corresponding author. Email: pangxy78@haust.edu.cn (X.Y. Pang); guoqiang2008@163.com (G.Q. Zhang); robert.henry@uq.edu.au (R.J. Henry).

Supporting information

The following materials are available in the online version of this article.

Figure S1. Analysis of the activity of the trypsin autolysis peptides.

Figure S2. Quality control of single-cell data (A) and co-expression patterns of marker genes (B) in the three samples.

Figure 3. RCTD and CARD methods for correlation analysis between stRNA-seq and scRNA-seq.

Figure S4. Expression of marker genes for the five components across three samples and five components.

Figure S5. Pseudotime analysis of cells of CK, Post, and TAP4 samples.

Figure S6. Metabolomics analysis of the pericarp of *H. undatus*.

Figure S7. Effects of silencing key genes on fruit phenotype and related gene expression in *H. undatus*.

Figure S8. Effects of TAP4 treatment on the postharvest microbial diversity of *H. undatus* fruit.

Supplemental Data 1. Activities of trypsin and autolytic peptide TAP4.

Sheet1 Superoxide scavenging activities of trypsin.

Sheet2 Effect of TAP4 on stoma in *H. undatus* fruit during postharvest storage.

Sheet3 Effect of TAP4 on cell size of *H. undatus* fruit during postharvest storage.

Sheet4 Effect of TAP4 on fluorescence intensity detected by RNA-FISH in *H. undatus* fruit during postharvest storage.

Sheet5 Effect of TAP4 on ROS detected by H_2DCFDA in *H. undatus* fruit during postharvest storage.

Sheet6 Effect of TAP4 on DAB staining in *H. undatus* fruit during postharvest storage.

Sheet7 Effect of TAP4 on lesion area of *H. undatus* fruit during postharvest storage.

Sheet8 Effect of TAP4 on callose deposition in *H. undatus* fruit during postharvest storage.

Supplemental Data 2. Information of trypsin autolytic peptides.

Sheet1 Identification of trypsin autolytic peptides by mass spectrometry.

Sheet2 The proportion of various amino acids in the seven key polypeptides.

Supplemental Data 3. Summary of the transcriptomic cell data in *H. undatus* pericarp samples.

Sheet1 Quality control information for the single-cell transcriptomic data.

Sheet2 Quality control information for the spatial transcriptomic data.

Sheet3 Expression of all 27735 genes in CK, Post, and TAP4 samples.

Sheet4 ROGUE value of 9 subcluster cells of EX, ME, and ENF cells.

Supplemental Data 4. Cluster information for 26 cell clusters of *H. undatus* pericarp cells.

Sheet 1 A comparative analysis statistical table of cell clusters presented by sample.

Sheet 2 A comparative analysis statistical table of cell types presented by sample.

Supplemental Data 5. Cluster-enriched gene list and gene descriptions for 26 cell clusters.

Supplemental Data 6. Correlation between 26 cell clusters.

Supplemental Data 7. Expression of pseudotime related immune genes.

Sheet 1 Regulatory patterns of 147 genes across 26 cell clusters.

Sheet 2 Expression levels of 147 genes in the three components of the three samples.

Supplemental Data 8. GRN parameters of pseudotime related genes by Cytoscape.

Sheet1 Parameters of nodes in the GRN.

Sheet2 Top20 nodes in PRGs network ranked by 8 effective methods of cytoHubba.

Sheet3 Information of all top20 nodes of GRN.

Sheet4 Expression of these top nodes in EX, ME, and ENF components in three samples.

Supplemental Data 9. Information of metabolites regulated by TAP4 in *H. undatus* analyzed by the SWATH method for mass spectrometry detection.

Sheet1 Information of top 26 metabolites regulated by TAP4.

Sheet2 Information of 3 betacyanins.

Sheet3 Information of all 3,015 metabolites detected in *H. undatus*.

Supplemental Data 10. Information of co-expression network among transcript factors, genes, and metabolites.

Supplemental Data 11. Information of hub genes shown in the heatmap of pseudotime analysis.

Sheet1 Information of genes in figure 4F.

Sheet2 Information of genes in figure 4G.

Supplemental Data 12. Key resources used in the current work.

Supplemental Data 13. Primer sequences used in this work.

Sheet1 Primers used in VIGS.

Sheet2 Primers used in RT-qPCR.

Sheet3 Primers used in RNA-FISH.

Supplementary Figures:

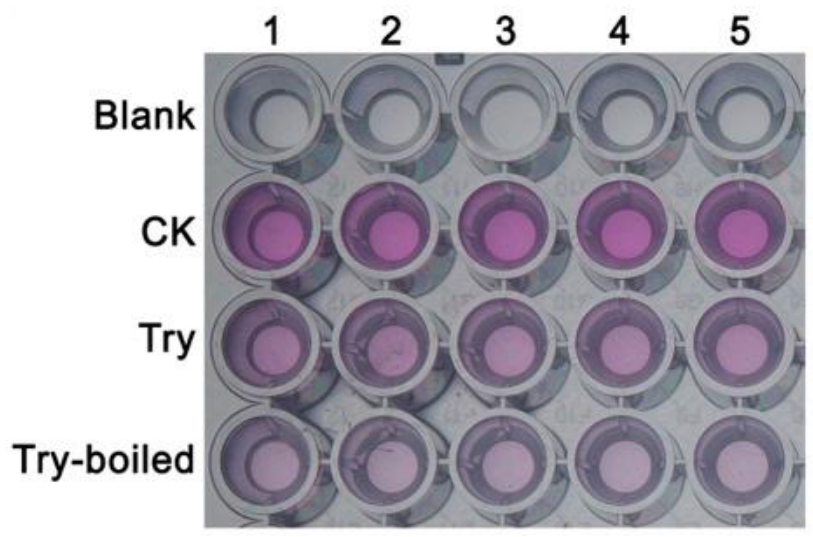

Figure S1: Analysis of the activity of the trypsin autolysis peptides. Color of the wells in the 96-well plate indicates the changes in superoxide anion levels after trypsin inactivation by boiling. The five columns represent five parallel samples for each group.

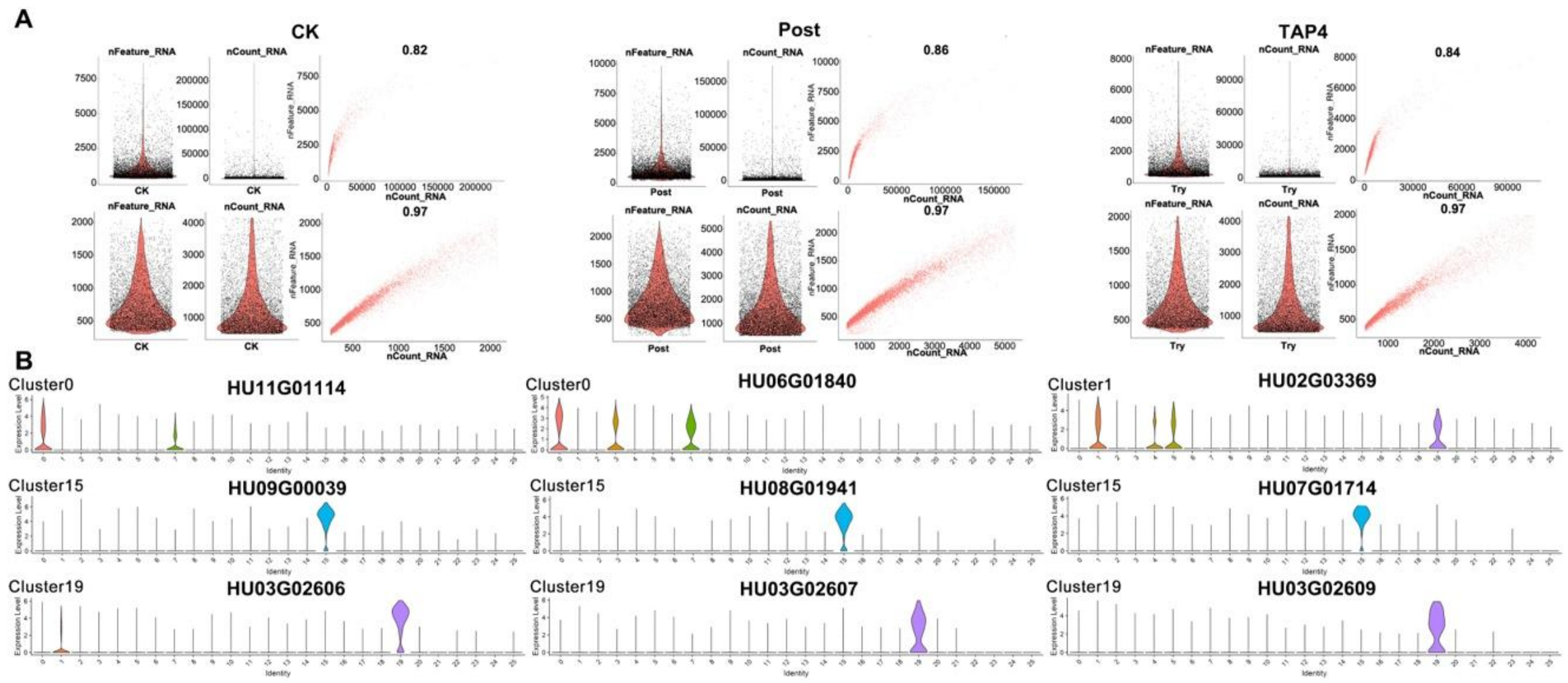

Figure S2: Quality control of single-cell data (A) and co-expression patterns of marker genes (B) in the three samples.

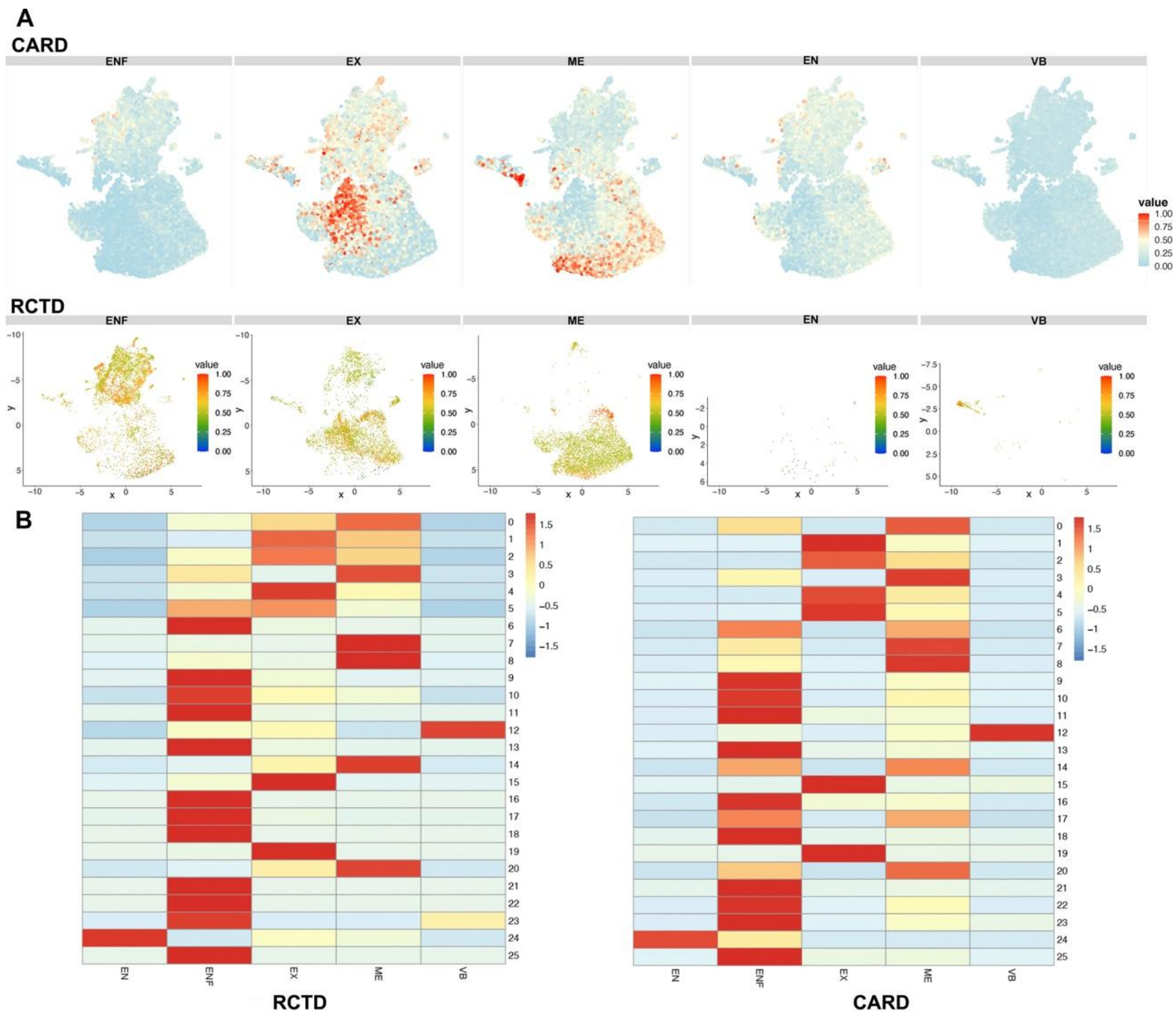

Figure S3: RCTD and CARD methods for correlation analysis between stRNA-seq and scRNA-seq. Heat dot plots in the UMAP (A) and the color bands in the heatmap (B) illustrate five components, respectively. The color gradient from red to blue indicates correlations ranging from high to low.

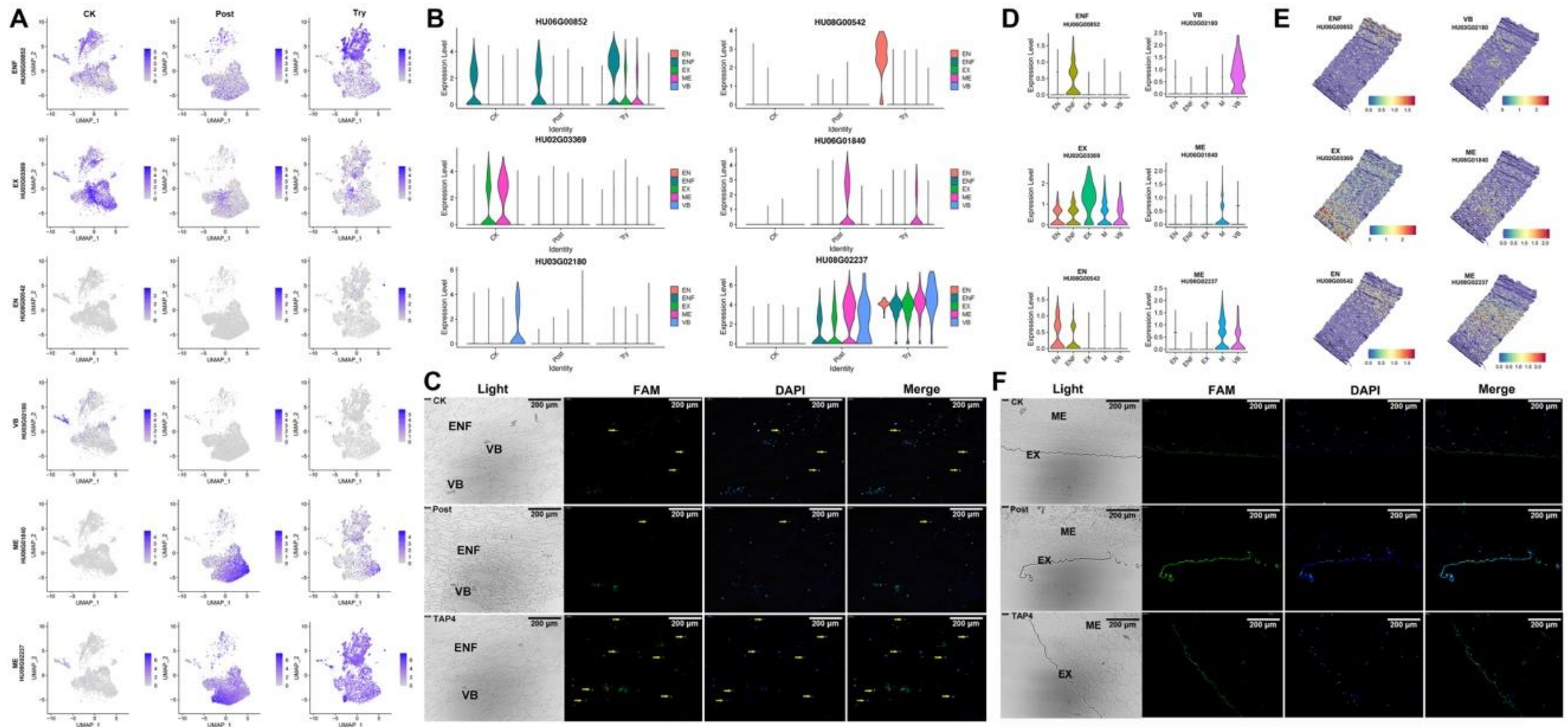

Figure S4: Expression of marker genes for the five components across three samples and five components

(A) The UMAP plots displayed the expression of each gene in the three samples.

(B) The violin plots illustrated the expression of each gene across the five components in the three samples. The three violin plots, from left to right, showed gene expression across the five components and three samples, respectively.

(C and F) RNA-FISH revealed the expression of the marker gene HU06G00852 and HU02G03369 in the endocarp and exocarp of the three samples, respectively.

(D-E) Spatial transcriptomic mapping (D) showing the spatial localization of marker gene expression, along with the corresponding violin plot (E).

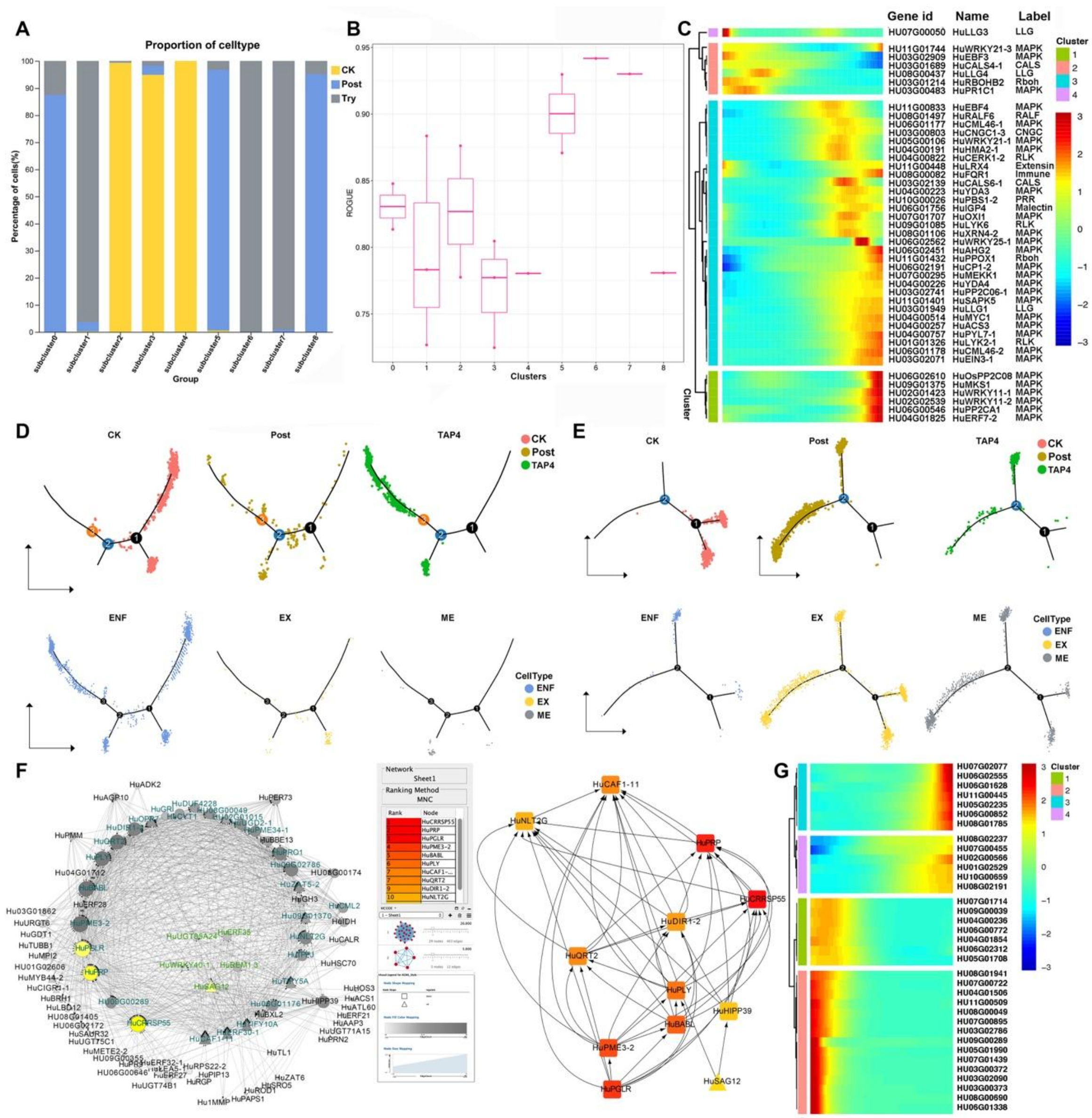

Figure S5: Pseudotime analysis of cells of CK, Post, and TAP4 samples

(A) Bar charts illustrating the statistical proportions of CK, Post, and TAP sample cells within each subcluster of EX, ME, and ENF cells.

(B) ROGUE evaluated the cluster purity of the nine subpopulations. Higher ROGUE values indicate better cell cluster quality. A ROGUE value of 1 represents a completely pure cell population, whereas values approaching 0 indicate highly heterogeneous mixed populations.

(C) Heatmap of expression of immune related genes along the pseudotime progression.

(D-E) Cells from the three samples (up) and three components (bottom) were mapped onto the pseudotime trajectory of cells high related to up- (D) and down-regulated (E) genes by TAP4.

(F) Overall diagram of the GRN (left) and the hierarchical layout of the top 12 nodes calculated by CytoHubba (right) of top 100 genes related to pseudotime from the MXF_1595 cells. The module parameters obtained from the MCODE plugin, the top nodes identified by the CytoHubba plugin, and the network drawing annotations are displayed in the center.

(G) Heatmap of expression of hub genes along the pseudotime progression.

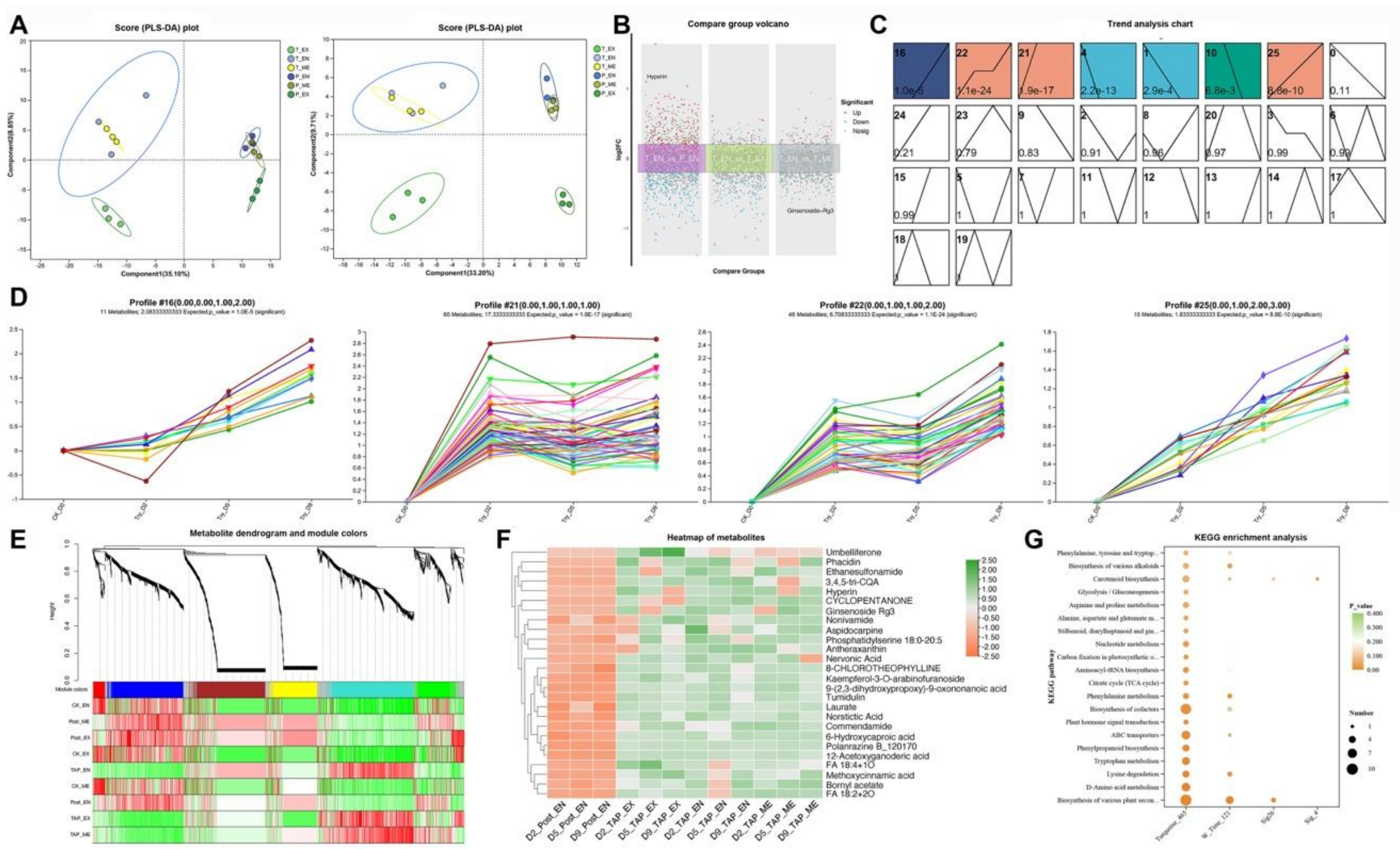

Figure S6: Metabolomics analysis of the pericarp of *H. undatus*

(A) PLS-DA analysis of metabolites identified in three components of Post samples (P_EX, P_EN, and P_ME) and TAP samples (T_EX, T_EN, and T_ME) under positive and negative ion modes, respectively.

(B) A differential pairwise comparison volcano plot displayed the distribution of characteristic metabolites with significant differences among the three groups.

(C) Temporal expression trend analysis of metabolites in each component using the STEM algorithm. Each profile represented a set of metabolites with similar trends, shown as a rectangle. The line within each rectangle depicted the temporal expression trend, while the bottom-left corner displayed the corresponding significance level (p value). Profiles with colored trends indicated significant temporal patterns, where profiles of the same color were grouped into a single cluster based on similar trends. Profiles without color indicated non-significant temporal patterns.

(D) K-means clustering analysis of the temporal expression trends of all metabolites within each profile from panel 6c. The lines represented the changes in expression levels over time.

(E) Cluster results and component heatmap of data samples based on WGCNA analysis.

(F) Hierarchical clustering heatmap showed the abundance of the top 26 metabolites, screened using a Venn diagram in Figure 5B, at three time points during storage.

(G) KEGG enrichment analysis showed the pathways of top 26 metabolites.

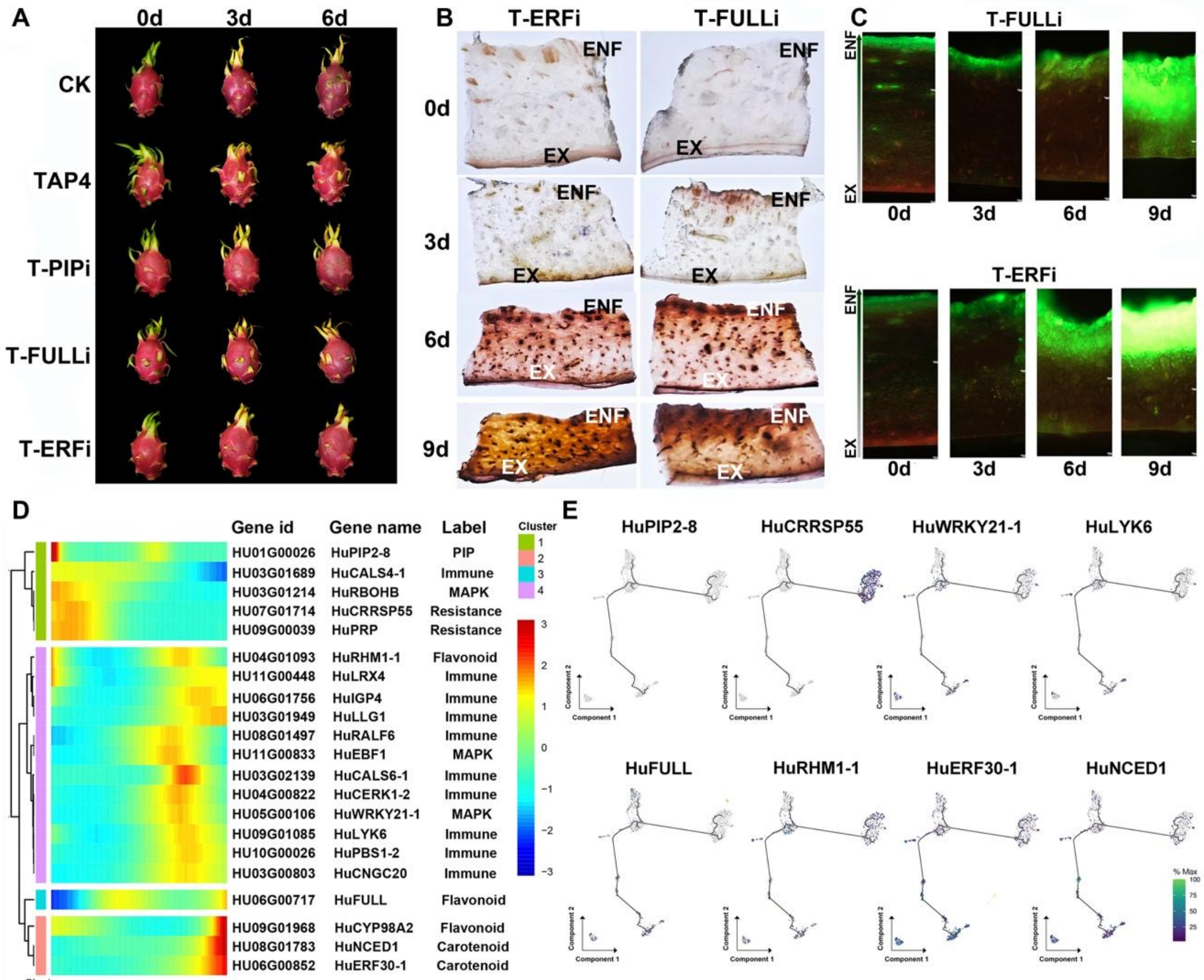

Figure S7: Effects of silencing key genes on fruit phenotype and related gene expression in *H. undatus*

(A-C) Changes in fruit phenotype (A), DAB staining of H_2O_2, and ROS localization shown by H2DCFDA probe (C) after silencing the hub genes

(D) Pseudotime heatmap showing the expression patterns of key genes.

(E) Expression of key genes mapped onto the pseudotime trajectory.

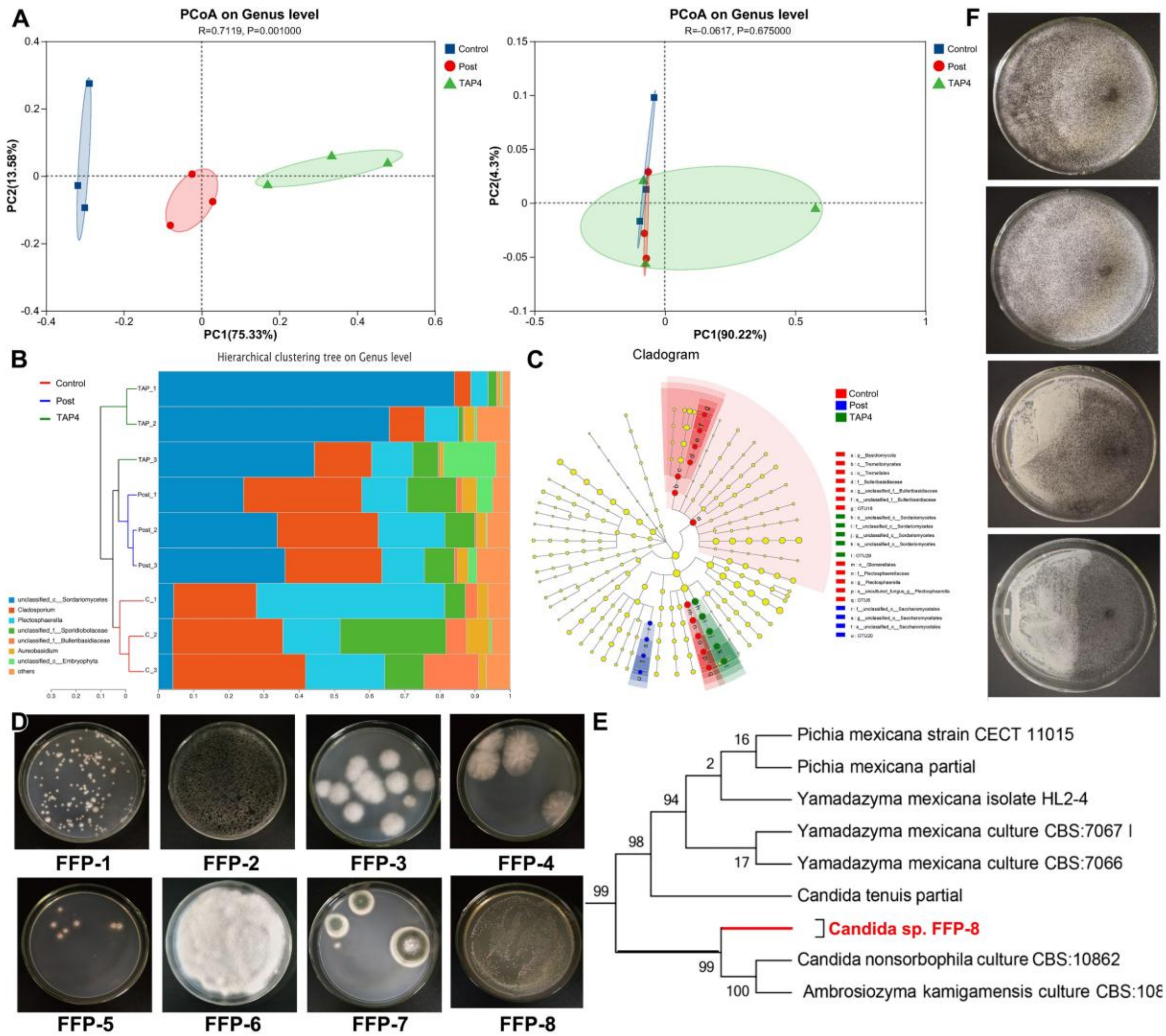

Figure S8: Effects of TAP4 treatment on the postharvest microbial diversity of *H. undatus* fruit

(A) PCoA analysis of fungal (left) and bacterial (right) community composition at the genus level.

(B) Beta diversity analysis of community composition across samples.

(C) LefSe multi-level taxonomic differential analysis from Kingdom to OUT level.

(D) Eight fungal strains (FFP1-8) isolated and purified from fruit lesions.

(E) Phylogenetic tree showing the taxonomic placement of strain FFP-8.

(F) Plate confrontation assays between yeast and mould. From top to bottom: Mould alone, TAP4 + Mould, Candidias + Mould, TAP4 + Candidias + Mould.
